# Nonsynonymous Substitution Rate Heterogeneity in the Peptide-Binding Region Among Different *HLA-DRB1* Lineages in Humans

**DOI:** 10.1534/g3.114.011726

**Published:** 2014-05-02

**Authors:** Yoshiki Yasukochi, Yoko Satta

**Affiliations:** * Molecular and Genetic Epidemiology, Faculty of Medicine, University of Tsukuba, Tsukuba, Ibaraki 305-8575, Japan; † Department of Evolutionary Studies of Biosystems, the Graduate University for Advanced Studies (SOKENDAI), Hayama, Kanagawa 240-0193, Japan

**Keywords:** allelic lineage, balancing selection, HLA, pathogen, peptide-binding region, genetics of immunity, innate immunity, complex genetics, tolerance, complex immunity, infection, resistance

## Abstract

An extraordinary diversity of amino acid sequences in the peptide-binding region (PBR) of human leukocyte antigen [HLA; human major histocompatibility complex (MHC)] molecules has been maintained by balancing selection. The process of accumulation of amino acid diversity in the PBR for six *HLA* genes (*HLA-A*, *B*, *C*, *DRB1*, *DQB1*, and *DPB1*) shows that the number of amino acid substitutions in the PBR among alleles does not linearly correlate with the divergence time of alleles at the six *HLA* loci. At these loci, some pairs of alleles show significantly less nonsynonymous substitutions at the PBR than expected from the divergence time. The same phenomenon was observed not only in the *HLA* but also in the rat *MHC*. To identify the cause for this, *DRB1* sequences, a representative case of a typical nonlinear pattern of substitutions, were examined. When the amino acid substitutions in the PBR were placed with maximum parsimony on a maximum likelihood tree based on the non-PBR substitutions, heterogeneous rates of nonsynonymous substitutions in the PBR were observed on several branches. A computer simulation supported the hypothesis that allelic pairs with low PBR substitution rates were responsible for the stagnation of accumulation of PBR nonsynonymous substitutions. From these observations, we conclude that the nonsynonymous substitution rate at the PBR sites is not constant among the allelic lineages. The deceleration of the rate may be caused by the coexistence of certain pathogens for a substantially long time during HLA evolution.

The human leukocyte antigen (HLA) system, also known as the major histocompatibility complex (MHC) in nonhumans, codes for molecules that bind to both self and nonself peptides and presents them to T lymphocytes. In humans, six classical HLA molecules, three of class I (HLA-A, B, and C) and three of class II (HLA-DR, DQ, and DP), have been identified as important for the initiation of T cell–mediated immune response.

The peptide-binding region (PBR) for each *HLA* is the most polymorphic coding region in the human genome, and this variation is thought to be primarily maintained by balancing selection (Hughes and Nei 1988, 1989; Hughes and Yeager 1998; Klein *et al.* 2007). There are three major (nonmutually exclusive) mechanisms of balancing selection: heterozygote advantage (overdominant selection) (Doherty and Zinkernagel 1975); rare allele advantage (Slade and McCallum 1992); and fluctuating selection (Hill 1991) [see Spurgin and Richardson (2010) for an in-depth review of these hypotheses].

Of the different mechanisms proposed for balancing selection, there are several studies that have identified an important role of the heterozygote advantage in maintaining genetic variation of the HLA. Heterozygote advantage acts when individuals who are heterozygous at a given locus have a higher fitness than individuals who are homozygous because they can bind a larger suite of antigens and confer resistance to a broader range of pathogens (Doherty and Zinkernagel 1975). The first study to provide molecular evidence for overdominant selection was conducted by Hughes and Nei (1989). They found that the rate of nonsynonymous substitutions in the PBR exceeded that of synonymous substitutions in the entire gene. Studies examining human resistance to specific pathogens have also indicated a role for heterozygote advantage. For example, heterozygotes for *HLA* class II loci were more resistant to the hepatitis B virus than homozygotes (Thursz *et al.* 1997). Similarly, later onset and slower progression of acquired immunodeficiency syndrome (AIDS) have been observed in heterozygotes for *HLA* class I loci, rather than in homozygotes (Carrington *et al.* 1999). In addition, a review of MHC studies in free-ranging animal populations highlighted the functional importance of MHC variability in parasite resistance (Sommer 2005). Similar studies have also shown associations between MHC polymorphism and parasite resistance, although some studies did not support the heterozygote advantage hypothesis (Penn *et al.* 2002; Musolf *et al.* 2004; Oliver *et al.* 2009).

In addition to balancing selection via amino acid substitutions in the PBR, evolutionary forces to the selection seem to be in operation, purifying selection due to functional (or structural) constraints. Consequently, nonsynonymous substitutions in the PBR show several characteristics. One is a frequent flip-flop substitution of Val and Gly at site 81 in the *HLA-DRB1* gene. This specific amino acid switching is believed to be due to functional or structural constraints for the peptide-binding groove (Ong *et al.* 1991; Demotz *et al.* 1993; Verreck *et al.* 1996). In addition, the extent of MHC diversity at the PBR appears to be limited, as too much diversity limits T lymphocyte repertoires as a result of extensive negative selection during the T cell development process in the thymus (Vidović and Matzinger 1988; Nowak *et al.* 1992; Woelfing *et al.* 2009).

Despite the unusually large amount of variation found at the MHC PBR, the theory (allelic genealogy) of allelic lineage under symmetric balancing selection (Takahata 1990; Takahata *et al.* 1992) predicts that nucleotide substitutions at the PBR sites are accumulated with the divergence time of the alleles. This is because symmetric balancing selection assumes that large numbers of alleles are equivalent to each other in their fitness, and thus the probability for producing descendants is also equal. Therefore, the genealogical characteristic is quite similar to the neutral case, except for its time scale (Takahata 1990). This time scale is determined by a “scaling factor” that is a function of the mutation rate, selection coefficient, and effective population size. For the molecular clock to operate for nonsynonymous substitutions in the PBR, the selection intensity for all the lineages must be constant. However, despite many studies that examined selective mechanisms of *HLA* diversity, no previous studies have elucidated the temporal aspects of nucleotide substitution patterns at the PBR nonsynonymous sites. To determine the temporal evolutionary trajectory of *HLA* diversity, amino acid substitution patterns at the PBR nonsynonymous sites were examined at six *HLA* loci (*HLA-A*, *B*, *C*, *DRB1*, *DQB1*, and *DPB1*), with special focus on *HLA-DRB1*. Here, we provide evidence for episodic amino acid substitutions in the PBR of humans, in primate evolution, suggesting significant fluctuation of selection intensity of the allelic lineage in *HLA-DRB1*. The extent of amino acid diversity in the PBR is directly linked to how many kinds of pathogens can be recognized by *HLA* molecules. Hence, tracing the transitional change of *HLA* diversity may allow us to describe the relationships between humans and pathogens in particular time periods.

## Materials and Methods

### Collection of nucleotide sequence data for six *HLA* loci

Nucleotide sequences for the six *HLA* loci (*HLA-A*, *B*, *C*, *DRB1*, *DQB1*, and *DPB1*) were obtained from the HLA/IMGT database (http://www.ebi.ac.uk/imgt/hla/) (Robinson *et al.* 2011). The sequences of *HLA-A*, *B*, *C*, and *DRB1* containing the entire coding region (*HLA-A*/*-B*/*-C*, approximately 1100 bp; *HLA-DRB1*, approximately 800 bp) were used in this analysis. Because the number of alleles covering the whole sequence of the coding region in the *HLA-DQB1* and *HLA-DPB1* was limited, slightly shorter sequences were included in the analysis (*HLA-DQB1*, approximately 690 bp; *HLA-DPB1*, approximately 540 bp). Possible recombinant alleles were excluded by using the method described by Satta (1992). This method assumes that the relationship between the number of substitutions in a particular region and that in the entire region is binomially distributed. Additionally, the ratio of the number of these substitutions is proportional to the size in the corresponding regions (see Kusaba *et al.* 1997 for more details). Consequently, the dataset used in this analysis included 50 *HLA-A* alleles, 143 *HLA-B*, 129 *HLA-C*, 56 *HLA-DRB1*, 55 *HLA-DQB1*, and 38 *HLA-DPB1*. *HLA-DRB1* alleles observed in a Japanese population were investigated using the Allele Frequency Net Database (http://www.allelefrequencies.net/) (Gonzalez-Galarza *et al.* 2011).

### Phylogenetic analyses

Multiple sequence alignments of *MHC-DRB1* nucleotide sequences and amino acid translations were performed using MEGA v5.10 (Tamura *et al.* 2011). A phylogenetic tree of nucleotide sequences in *DRB1* non-PBR was constructed based on the maximum-likelihood (ML) method using the Hasegawa-Kishino-Yano (HKY) substitution model (Hasegawa *et al.* 1985) implemented in MEGA. Nearest-neighbor-interchange (NNI) was applied as the ML heuristic method. Bootstrap values were obtained from 1000 replications. Amino acid distances at the PBR between alleles were calculated using the Jones-Taylor-Thornton (JTT) model (Jones *et al.* 1992) as per the ML tree topology for non-PBR using software in the PHYLIP 3.69 package (Felsenstein 2009). In the phylogenetic analysis of 56 *HLA-DRB1* alleles (described above), 6 *HLA-DRB3*, 4 *HLA-DRB4*, 2 *HLA-DRB5*, 11 chimpanzee (*Pan troglodytes*) *Patr-DRB1*, 22 rhesus monkey (*Macaca mulatta*) *Mamu-DRB1*, and 3 crab-eating macaque (*Macaca fascicularis*) *Mafa-DRB1* sequences were also included after possible recombinant alleles were removed using the method by Satta (1992) (see above). Two *HLA-DQB1* sequences (HLA-DQB1*02:01:01 and HLA-DQB1*06:02:01) were used for determining the root of the tree. The dataset of *HLA-DRB3*, *HLA-DRB4*, *HLA-DRB5*, and *HLA-DQB1* allele sequences was obtained from the HLA/IMGT database, whereas the nonhuman primate *DRB1* sequences were obtained from the IPD-MHC NHP database (http://www.ebi.ac.uk/ipd/mhc/nhp/) (Robinson *et al.* 2005).

### Examination for temporal aspect of amino acid substitution pattern in the PBR

Sites 57, 67, and 90 were not identified as the PBR sites of HLA-DRB1 molecule by Brown *et al.* (1993). However, Brown and collaborators have subsequently shown that these sites are involved in peptide-binding pockets that are crucial for peptide capture (Stern *et al.* 1994). In addition, recent assays for the peptide-binding prediction of HLA molecules have re-identified these three sites as peptide-binding residues (Reche and Reinherz 2003). Therefore, they were included as PBR in this analysis, and 27 amino acids in total were regarded as PBR. To examine the nucleotide substitution rate, the mean number of nonsynonymous substitutions in PBR [*K*_B(_*_m_*_)_] among allelic pairs that had the particular number of substitutions (*m* = *K*_S_ + *K*_N_, where *K*_S_ is the number of synonymous substitutions in the entire region and *K*_N_ is the number of nonsynonymous substitutions in non-PBR) at putative neutral sites (synonymous + non-PBR nonsynonymous sites) was examined. The molecular clock for *K*_N_ was tested using the ML method in MEGA.

The expected *K*_B(_*_m_*_)_ value was calculated on the basis of equation 12 described in Takahata *et al.* (1992), and its SE was estimated by maximum variance (Takahata and Tajima 1991). The variables necessary for the equation were selected such that the first several observations showed good agreement with expectations.

### Estimation of the divergence time of *HLA-DRB1* alleles and amino acid substitution rates in the PBR

We calculated the divergence time of allelic pairs on the basis of neutral divergence, *d =* (*K*_S_ + *K*_N_/*f*)/(*L*_S_ + *L*_N_), where *L*_S_ and *L*_N_ are the number of synonymous and non-PBR nonsynonymous sites, respectively. The *K*_N_*/f* value is the number of converted neutral substitutions obtained by dividing by the functional constraint *f*, which is the mean ratio of nonsynonymous to synonymous substitution rates at the non-PBR of all allelic pairs. The divergence time (*T*) of each allele pair is given by the formula *T* = *d*/2*μ*, where *μ* is the neutral substitution rate of 10^−9^ per site per year at the primate MHC loci (Satta *et al.* 1994).

To evaluate the amino acid substitution rate in the PBR for each allelic lineage, the number of PBR amino acid substitutions on each branch of the non-PBR ML tree was compared with the ML estimate of the corresponding branch length. Here, the linear correlation between these two values was normally expected if the molecular clock worked in both PBR and non-PBR. Significant outliers were identified based on 95% confidence interval of the regression line. These outliers were further examined for whether the ML estimate of a branch length was significantly different from zero, based on a confidence interval of the estimates. In addition, internodal branches supported by more than 80% bootstrap values were selected. Finally, we identified allelic lineages or branches with a fast or slow PBR substitution rate compared with the average.

### Computer simulation and prediction of peptide-binding specificity for *HLA-DRB1* alleles

The assumptions underlying the computer simulation were as follows. We supposed that there is a PBR phylogenetic relationship that shows the same topology of the non-PBR tree based on nucleotide sequences of 56 *HLA-DRB1* alleles. According to the expected time length (*T*_i_) for a branch *i*, the number of PBR substitutions (*K*_i_) on the branch follows a Poisson distribution with mean *λ* = *μT*_i_, where *μ* is the substitution rate (=1/unit time). The *T*_i_ value is the number of neutral nucleotide substitutions corresponding to each branch length on the non-PBR tree. The *K*_i_ value on a slow-rate branch was determined by dividing the original *λ* value by 10, whereas that on a fast branch was calculated by multiplying the original *λ* value by 10. In this manner, *K*_i_ values for every branch were determined and the number of substitutions in a pair of alleles was obtained by summing corresponding branches.

The dataset of epitopes or source organisms bound to HLA-DRB1 molecules was obtained from the Immune Epitope Database (IEDB) (http://www.immuneepitope.org) (Vita *et al.* 2010).

## Results

### Variation in amino acid diversity at the PBR

**Figure 1:**
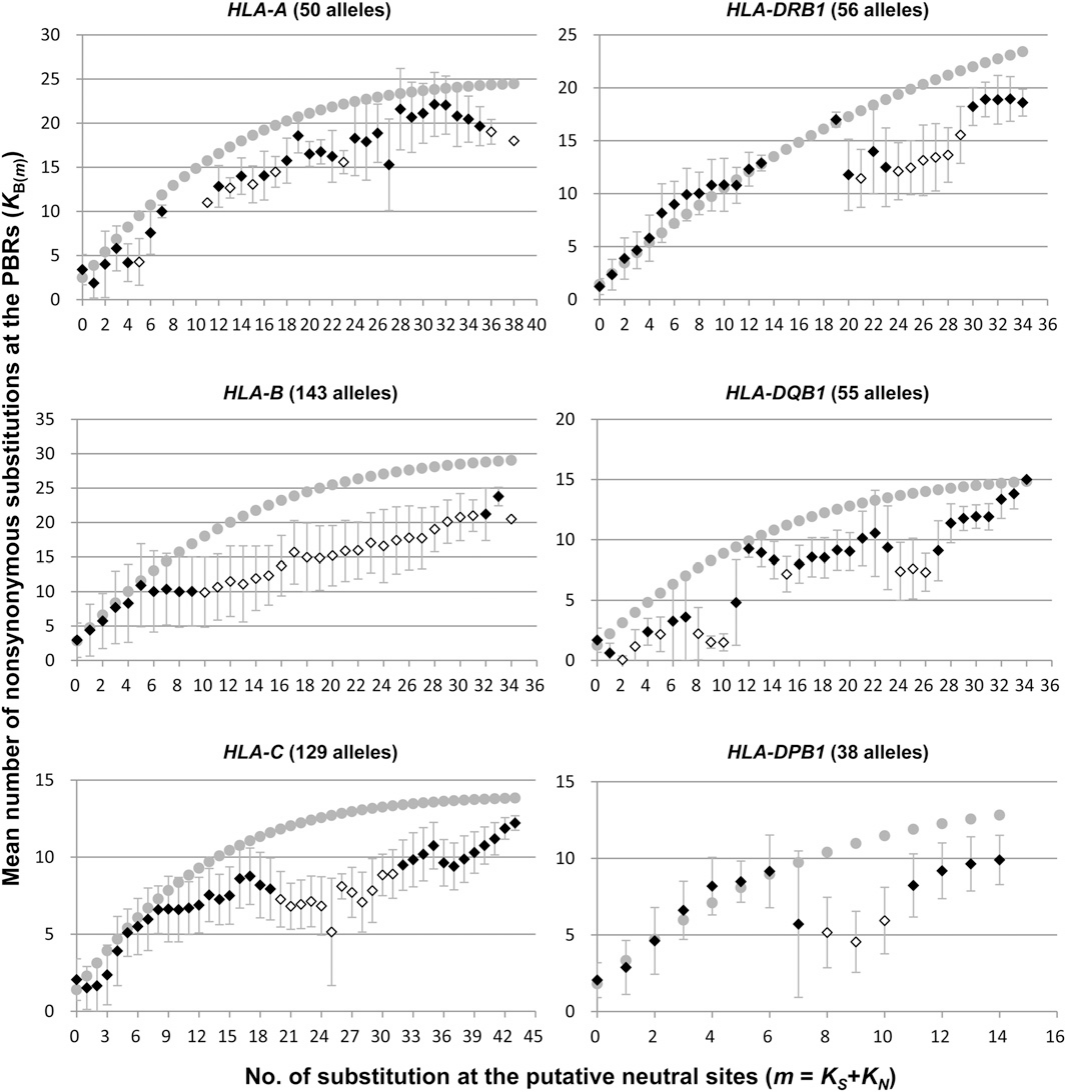
The level of amino acid diversity at the peptide-binding region among *HLA* allele pairs that share the same coalescence time. The ordinate axis indicates the mean number of nonsynonymous substitutions at the peptide-binding region (PBR) among allele pairs [*K*_B(_*_m_*_)_]. The abscissa axis indicates the number of substitutions at putative neutral sites (*m* = *K_S_* + *K_N_*). The gray dot represents the expected *K*_B(_*_m_*_)_ value. The diamond represents the observed *K*_B(_*_m_*_)_ value. The open diamond indicates the statistically significant difference between the observed and expected *K*_B(_*_m_*_)_ values (*P* < 0.05).

For each of the six *HLA* loci, the mean number of nonsynonymous substitutions in the PBR [*K*_B(_*_m_*_)_] among particular allele pairs with putative neutral substitutions of *m* (*m* = *K*_S_ + *K*_N_) was calculated (Figure 1). Since the *K*_N_ is the number of nonsynonymous substitutions at the non-PBR, we performed molecular clock tests for the substitutions. As a result, the homogeneous evolutionary rate of *K*_N_ in each *HLA* locus was statistically supported (*P* < 0.05), with the exception of *HLA-B* and *HLA-C* loci. According to the theory previously described (Takahata *et al.* 1992), the value of *K*_B(_*_m_*_)_ is expected to increase in proportion to *m*, the coalescent time (or divergence time) between alleles, and the value is then saturated with *m* of distantly related allele pairs (Supporting Information, Figure S1). However, the observed *K*_B(_*_m_*_)_ value did not show the linear relationship with *m* for most loci (Figure 1). For the intermediate values of *m*, it appeared to decrease and cease the accumulation of the substitutions. After this plateau, the *K*_B(_*_m_*_)_ value increased again. Although most of the *HLA* genes showed a generally similar pattern of *K*_B(_*_m_*_)_ distribution, their detailed patterns were somewhat different for each locus. For instance, *K*_B(_*_m_*_)_ values in *HLA-DQB1* decreased until *m* = 2, whereas those in *HLA-DPB1* decreased from *m* = 6 to *m* = 9. Interestingly, our preliminary research indicated that the rat (*Rattus norvegicus*) *DRB1* (*RT1-Db1*) also showed a similar pattern of *K*_B(_*_m_*_)_ saturation (Figure S2).

The comparison between the observed and expected *K*_B(_*_m_*_)_ values showed that the observed *K*_B(_*_m_*_)_ was quite lower than expected in all *HLA* loci, and the difference was statistically significant (Z test, *P* < 0.05) (Figure 1). This result indicates that *K*_B(_*_m_*_)_ fluctuation was not likely caused by stochastic error. Instead, nonsynonymous substitutions at the PBR may have been unexpectedly suppressed due to an unknown biological phenomenon.

**Figure 2:**
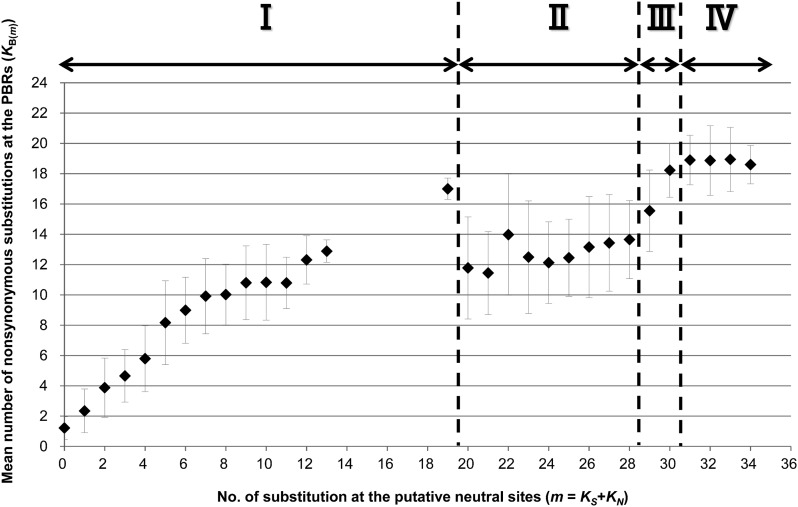
The mean number of nonsynonymous substitutions at the PBR among *HLA-DRB1* allele pairs that share the same coalescence time. The ordinate axis represents the mean number of nonsynonymous substitutions at the PBR among allele pairs [*K*_B(_*_m_*_)_]. The abscissa axis represents the number of substitutions at putative neutral sites (*m* = *K*_S_ + *K*_N_). The Roman numerals indicate the four phases that are classified as per variation in patterns of *K*_B(_*_m_*_)_ values (see text). Error bars indicate the SD from the mean.

An acceleration of *K*_B(_*_m_*_)_ was expected in order to cope with the rapid evolutionary change of pathogens; however, we observed a decline of *K*_B(_*_m_*_)_, and the reason was unexplained. It was intriguing to understand why amino acid substitutions at the PBR were suppressed despite balancing selection. Therefore, we investigated why *K*_B(_*_m_*_)_ did not increase in proportion to the divergence time of alleles (*m*). We explored *HLA-DRB1* alleles, which showed a typical graphical pattern and provided relatively rich experimental data for binding peptides. Depending on the extent of the *K*_B(_*_m_*_)_ increment, we categorized the graph into four phases, I through IV (Figure 2). In phases I and III, *K*_B(_*_m_*_)_ increase was proportional to *m*, the divergence time between alleles, whereas in phases II and IV, *K*_B(_*_m_*_)_ values were more or less constant, irrespective of the *m* values. In phase IV, the accumulation of substitutions might have reached a ceiling because of an enhanced PBR nonsynonymous nucleotide substitution rate due to balancing selection. In phase II, the *K*_B(_*_m_*_)_ values [*m* = 20 to 28, mean *K*_B(_*_m_*_)_ = 12.7] appeared to be extensively decreased as compared to that [*m* = 19, *K*_B(_*_m_*_)_ = 17.0] in phase I, suggesting that PBR amino acid substitutions of alleles in phase II were limited. Table S1 shows the allelic pairs that constituted phase II. When we approximately estimated the divergence time of allele pairs in phase II (see *Materials and Methods*), the divergence time ranged from approximately 18 MYA to 24 MYA, suggesting that those allele pairs have diverged at least before speciation events in the subfamily Homininae (or Hominidae). The *K*_B(_*_m_*_)_ decline was not observed in smaller *m*, suggesting that the decline had not occurred within the human lineage only.

### Phylogenetic analysis for the *DRB1* locus

**Figure 3:**
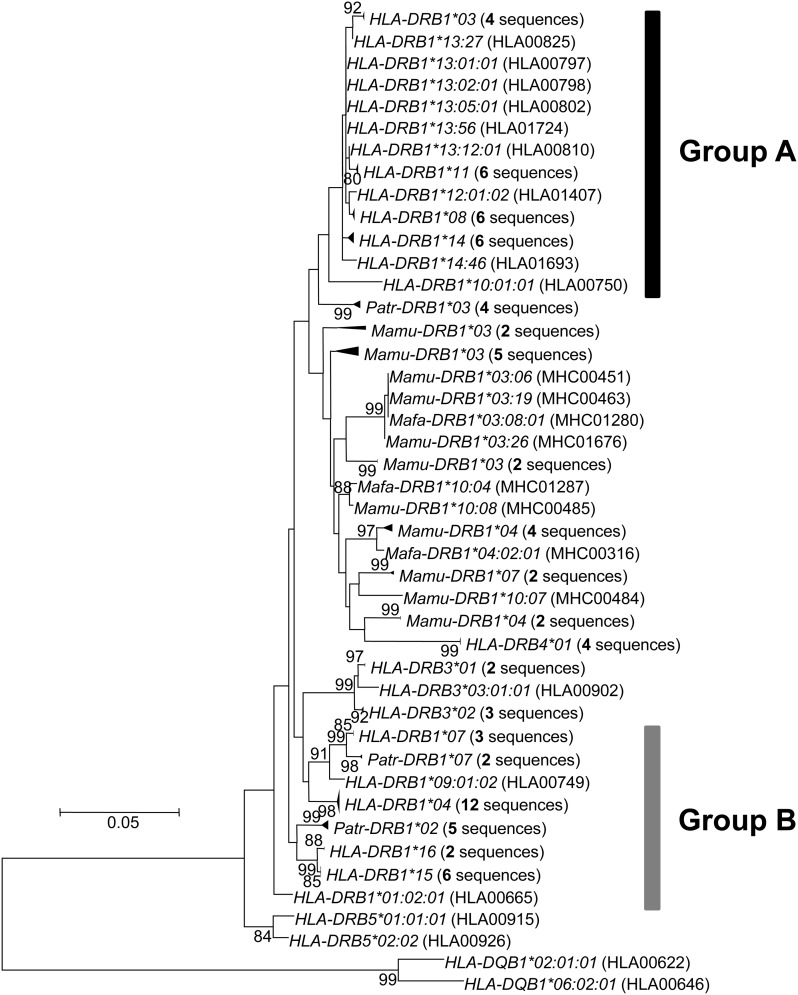
Phylogenetic relationships between *MHC DRB* alleles (including those of humans, chimpanzees, and macaques) as determined by the maximum likelihood (ML) method on the basis of nucleotide sequences (690 bp) in the non-PBR. Only bootstrap values more than 80% are shown here. Two *HLA-DQB1* sequences are used as the out-group. Evolutionary distances were computed using the Hasegawa-Kishino-Yano (HKY) model. Groups A and B indicate two major phylogenetic groups of *HLA-DRB1* alleles. *HLA*, humans; *Patr*, chimpanzees; *Mamu*, rhesus monkeys; *Mafa*, crab-eating macaques. IMGT/HLA and IPD accession numbers are in parentheses.

We constructed an ML tree for *DRB* alleles, including *HLA-DRB1*/*3*/*4*/*5*, *Patr-DRB1*, *Mamu-DRB1*, and *Mafa-DRB1*, based on total substitutions in only non-PBR nucleotide sequences. In the tree, some *HLA-DRB1* alleles formed a monophyletic group, whereas other alleles were polyphyletic with *DRB* alleles from nonhuman primates (Figure 3). This topology was also supported by the neighbor-joining (NJ) tree (Saitou and Nei 1987) method based on both nucleotide and amino acid sequences in the region, although bootstrap values of their groups were not high (Figure S3). Here, we refer to these two groups as group A and group B, respectively (Figure 3). Among the 14 known *HLA* allelic lineages, group A consisted of seven lineages, namely DRB1*03, *08, *10, *11, *12, *13, and *14. The remaining seven lineages, DRB1*01, *02, *04, *07, *09, *15, and *16, were in group B. The allelic lineages HLA-DRB1*01 and *10 of the same *DR1* haplotype were included in different groups, whereas other alleles of the same haplotype belonged to either group A or group B.

When the non-PBR phylogeny (Figure 3) based on total nucleotide substitutions was compared with the tree based on PBR amino acid substitutions, the phylogenetic position of allelic lineages in the non-PBR tree did not correspond to that in the PBR amino acids tree (Figure S4). This means that when members of an allele pair are distantly related to each other in their non-PBR sequences, the amino acids in the PBR are often more closely related (and vice versa). When the allele pairs in phase II were placed on the tip of non-PBR tree, each allele for a particular pair was frequently found (73% of allele pairs in phase II) from either group A or group B.

The constant accumulation of PBR nonsynonymous substitutions appears to be violated in certain allele pairs. The reasons for the observed nonlinearity of *K*_B(_*_m_*_)_ includes one of the following possibilities: (1) saturation of transitions in nucleotide substitution precedes that of transversion, and this difference in saturation timing causes the phenomenon; (2) parallel substitutions in the PBR mask the linear relationship between *K*_B(_*_m_*_)_ and *m*; or (3) slow-down of the PBR nonsynonymous substitution rate in particular allelic lineages skews the constant *K*_B(_*_m_*_)_ accumulation.

### Biased rate of transitions and transversions and parallel substitutions in the PBR among *HLA-DRB1* allele pairs

It is well-known that transitions (Ts) occur at a higher frequency than transversions (Tv) in both animal mitochondrial and nuclear DNA sequences. Because of this bias and the lower number of Ts than Tv states, Ts is likely to reach a plateau earlier than Tv. Thus, there is a possibility that excess of Ts in the *K*_B(_*_m_*_)_ explains the observed accumulation pattern. To examine this possibility, we tested the relationship between numbers of Ts and Tv in the PBR (Figure S5). In phase I, Ts values of 0.7 to 6.5 were observed with Tv of 0.6 to 11.0; in the phase II, Ts of 4.5 to 6.7 were seen with Tv of 7.7 to 11.0; in phase III, Ts of 5.5 to 7.3 were associated with Tv of 10.6 to 14.5; and in phase IV, Ts of 4.6 to 7.1 were found with Tv of 14.0 to 16.6. Although Ts values seem to be saturated when Tv = 14, the corresponding allele pairs were all in phase III or phase IV. Thus, the timing of Ts saturation did not explain the cessation of *K*_B(_*_m_*_)_ accumulation in phase II.

The non-PBR phylogenetic relationship among *HLA-DRB1* sequences was not consistent with the PBR phylogenetic relationship. This is likely due to parallel substitutions or recombination between alleles, and these events probably mask the linear relationship between *K*_B(_*_m_*_)_ and *m*. Because we excluded the possibility of recombinants when filtering alleles used in this analysis (see *Materials and Methods*), the effect of recombination on the *K*_B(_*_m_*_)_ accumulation pattern is quite small. Next, parallel substitutions in the PBR were investigated. An example of a parallel substitution is the substitution between Val and Gly at the site 81 (Gregersen *et al.* 1986), which was frequently observed on several branches in the non-PBR tree. Dimorphism (Val and Gly) at the site has been shown to affect the conformation of the peptide-binding pocket (Ong *et al.* 1991) and allo-recognition of peptides (Demotz *et al.* 1993). This dimorphism appears to be associated with the selection of binding-peptide groups with different motifs (Verreck *et al.* 1996). However, this parallel substitution at site 81 does not explain the phase II substitution pattern in *K*_B(_*_m_*_)_ (see below).

We performed an extensive search for parallel substitutions at the PBR amino acid sites by manually placing the PBR amino acid substitutions on branches in the non-PBR tree (Figure S4). The number of PBR substitutions was estimated by the maximum parsimonious method. Here, the same amino acid substitution on different branches was defined as parallel substitution. The number of such parallel substitutions was counted in each allele pair, not only in phase II but also in other phases. Consequently, it is interesting that a large number of parallel substitutions are observed even in closely related pairs of alleles as frequently as in distantly related allele pairs. Parallel substitutions at not only site 81 but also other PBR sites were widely observed across all phases (I–IV) (Table S2). However, *K*_B(_*_m_*_)_ values in phase I and phase III still increased with *m*, even though parallel substitutions were observed in these phases. Therefore, *K*_B(_*_m_*_)_ saturation in phase II was unlikely to be affected by parallel substitutions.

### Heterogeneity in the PBR substitution rate among allelic lineages

**Figure 4:**
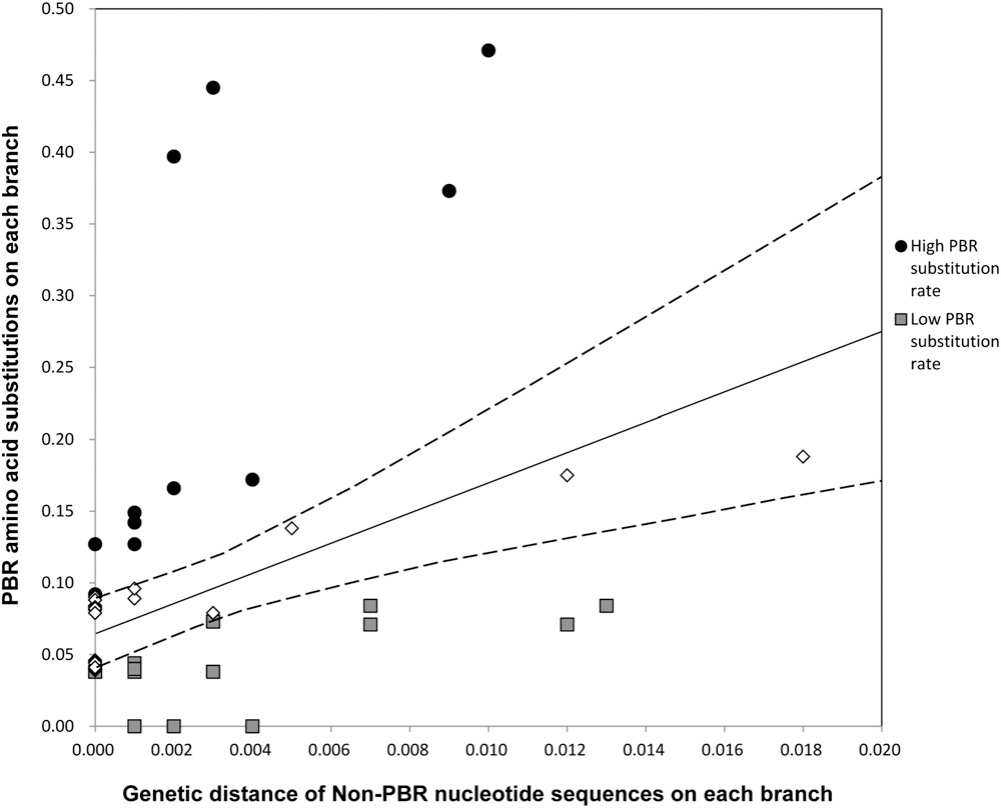
The relationship between the PBR and non-PBR genetic distances on each branch of the phylogenetic tree. The ordinate axis represents amino acid substitutions at the PBR among allele pairs on each branch of the non-PBR ML tree. The substitutions were estimated by the JTT model. The abscissa axis represents nucleotide substitutions at the non-PBR among allele pairs on each branch of the ML tree. The substitutions were estimated by the HKY model. The solid line represents a linear regression. The broken line represents the 95% confidential interval for the regression coefficient.

**Figure 5:**
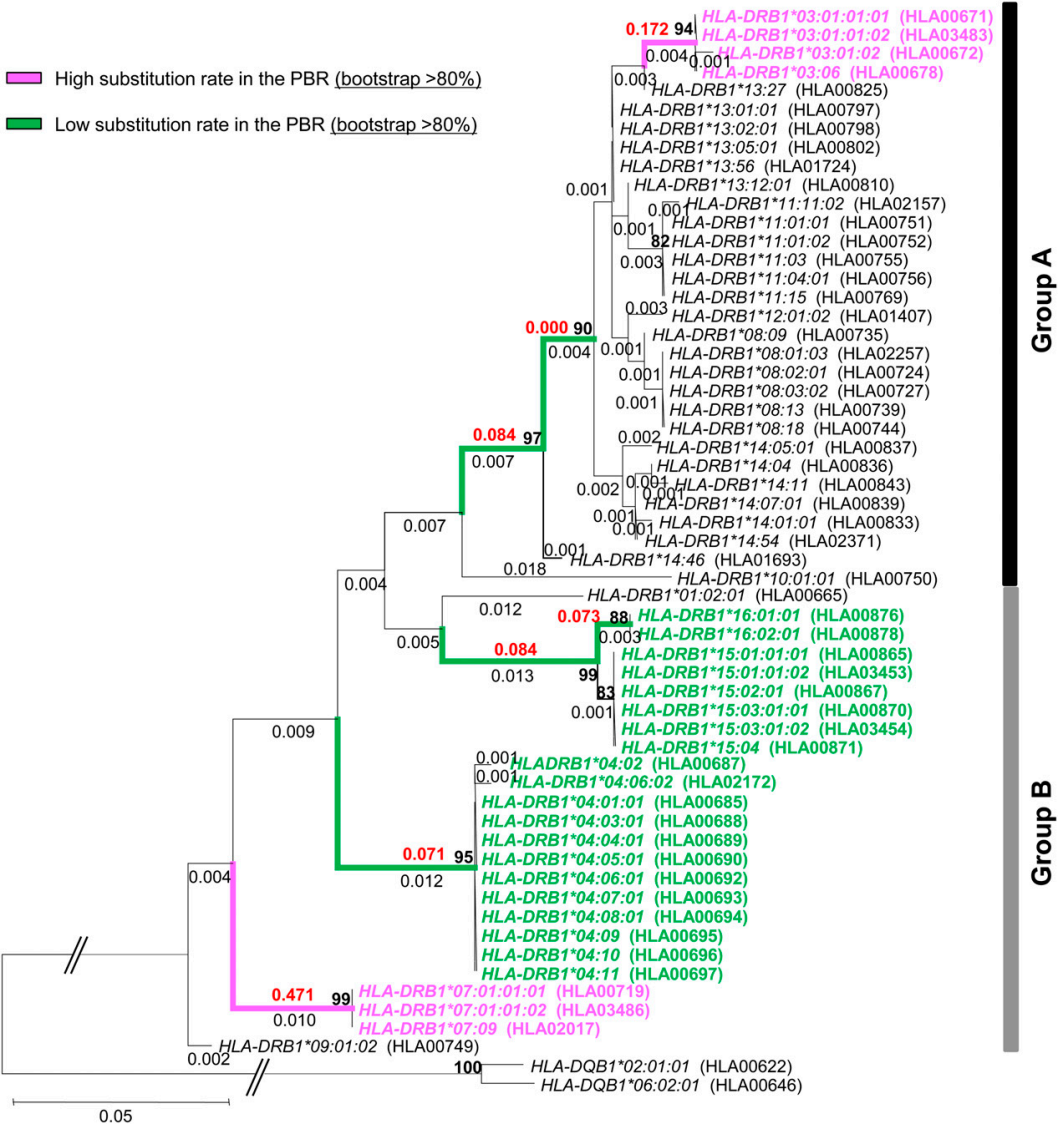
Phylogenetic relationships between *HLA-DRB* alleles as determined by the ML method on the basis of nucleotide sequences (690 bp) in the non-PBR. Only bootstrap values more than 80% are shown here. The values are shown by a bold character. Two *HLA-DQB1* sequences are used as the out-group. Evolutionary distances were computed using the HKY model. Group A and group B indicate two major phylogenetic groups of *HLA-DRB1* alleles. IMGT/HLA accession numbers are in parentheses. The numeric character shown in black on each branch of the tree represents nucleotide substitutions at the non-PBR among allele pairs. The numeric character shown in red on each branch of the tree represents amino acid substitutions estimated by the JTT model at the PBR among allele pairs. Branches and alleles with slow PBR amino acid substitutions are shown in green, whereas those with fast PBR substitutions are shown in pink.

To evaluate the possibility of heterogeneity in the substitution rate, we tested whether the PBR amino acid substitution rate for each allelic lineage was constant by means of the comparison between the number of nonsynonymous substitutions at non-PBR and PBR sites on each branch of the non-PBR tree (see *Materials and Methods*). This analysis identified five and two branches with significantly slow and fast PBR substitution rates, respectively, as compared with the average (Figure 4 and Figure 5). Allelic lineages of HLA-DRB1*04, *15, and *16 had a slow PBR substitution rate, and those of *03, and *07 had a fast rate. As expected, alleles with slow PBR substitution rates were observed frequently (44%) in phase II, whereas in phases I, III, and IV the frequencies of such alleles were 1–6% (Table 1).

**Table 1:** The frequency of alleles with fast or slow PBR substitution rates in phase I to IV

	Phase I		Phase II		Phase III		Phase IV	
	Fast	Slow	Fast	Slow	Fast	Slow	Fast	Slow
Frequency of counts*^a^*^,^ *^b^*	0.10	0.01	0.01	0.44	0.15	0.06	0.47	0.04
Counts*^a^*	107	8	10	757	16	6	94	7
No. of allele pairs*^a^* (×2)*^b^*	537 (1074)		851 (1702)		53 (106)		99 (198)	

a When both alleles in an allelic pair include the allelic lineage with fast or slow PBR substitution rates, the frequency or number is counted twice. When an allelic pair includes both allelic lineages with fast and slow PBR substitution rates, the frequency or number is not counted.

b Denominator is twice the total number of allele pairs in each phase.

**Figure 6:**
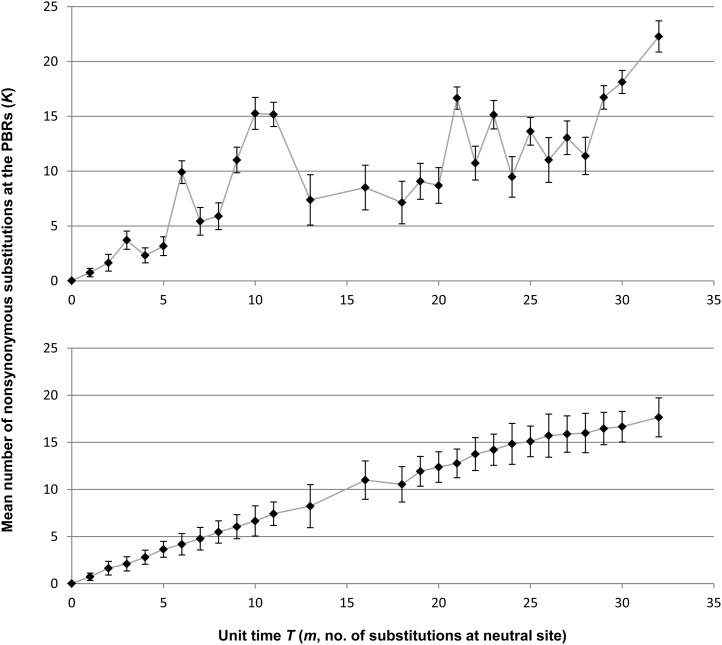
The *K* values of allele pairs that share the same coalescent time *T*. The ordinate axis represents the *K* value. The abscissa axis represents unit time *T* corresponding to the number of substitutions at neutral sites (*m*). The upper graph represents the accumulation of the mean number of nonsynonymous substitutions in the PBR among allele pairs (*K*) when PBR substitution rates of particular branches in a phylogenetic tree are slowed down or enhanced. The bottom graph represents the accumulation of *K* values without reduction or enhancement of PBR substitution rates. Error bars indicate the SD from the mean. The simulations for upper and bottom graphs were repeated 200 times and 100 times, respectively.

A computer simulation was performed to confirm whether the presence of such lineages with slow and fast substitution rates led to the nonlinearity of *K*_B(_*_m_*_)_ accumulation using 56 fictitious alleles that had the same phylogenetic relationship as the non-PBR tree (for details of the simulation methods, see *Materials and Methods*). After the simulation was repeated 200 times, we examined the relationship between the divergence time of alleles and the number of substitutions in the 56 allele pairs (Figure 6). As expected, *K*_B(_*_m_*_)_ values estimated in the computer simulation exhibited a similar pattern as the observation shown in Figure 2. We also performed a similar computer simulation (100 replications) without a reduction or enhancement of the PBR substitution rate. The simulation showed that *K*_B(_*_m_*_)_ values proportionally increased with the divergence time among the alleles (*m*). These results suggest that PBR nonsynonymous substitutions of *HLA-DRB1* alleles were decelerated or enhanced because of the heterogeneity of the PBR substitution rates among the allelic lineages.

## Discussion

### The cessation of accumulation in amino acid variation at the PBR

Contrary to expectations, *HLA* loci showed a *K*_B(_*_m_*_)_ decline in intermediate values of *m*. We estimated the *m* value on the basis of *K*_S_ and *K*_N_ in the analysis. It is possible that the *K*_B(_*_m_*_)_ decline was caused by the heterogeneous evolutionary rate of *K*_N_. Although constant non-PBR nonsynonymous substitution rates in the *HLA-B* and *HLA-C* were not supported by the molecular clock test, class II loci showed molecular clock in non-PBR nonsynonymous substitutions. Therefore, the unequal substitution rate of *K*_N_ values does not necessarily affect the *K*_B(_*_m_*_)_ decline.

The symmetric balancing selection model assumes that all *HLA* alleles are sampled from a panmictic population. To investigate the possibility that the biased sampling of sequences may result in the *K*_B(_*_m_*_)_ accumulation pattern, we examined the relationship between *K*_B(_*_m_*_)_ and *m* using *HLA-DRB1* alleles from a Japanese population, which is a putative panmictic population. The result showed that even if the *DRB1* alleles from a putative panmictic population were used, the pattern of *K*_B(_*_m_*_)_ decline in intermediate values of *m* was still observed (Figure S6). Therefore, *K*_B(_*_m_*_)_ reduction was unlikely to be caused due to the inappropriate sampling of *HLA* alleles in the present study.

Although possible recombinant alleles were removed from the analyses of the present study by Satta’s method (Satta 1992), we further investigated for reciprocal recombination or gene conversion using the GENECONV program (Sawyer 1989) to dismiss the possibility that recombination caused *K*_B(_*_m_*_)_ deceleration. Permutation test for the sequences used in this analysis indicated intragenic recombination between some pairs of alleles. However, the Bonferroni-corrected BLAST-like test showed no significant values. Even though possible recombinants (19 alleles) estimated by both of the permutation tests in GENECONV and Satta’s method were excluded from the examination of the relationship between *K*_B(_*_m_*_)_ and *m*, *K*_B(_*_m_*_)_ deceleration was still observed in 45 nonrecombinant alleles (Figure S7). Therefore, filtering of recombinants in the present study was unlikely to skew the *K*_B(_*_m_*_)_ distribution.

Because *K*_B(_*_m_*_)_ decline was also observed in the *K*_N_ and *K*_S_ comparison (Figure S8), it was interesting to know whether such *K*_N_ saturation affected the *K*_B(_*_m_*_)_ accumulation in proportion to *K*_S_ + *K*_N_. This question was addressed in the following manner. We assumed that *K*_B(_*_m_*_)_ values increase proportionately with *K*_S_, but that the *K*_N_ value is somewhat saturated for intermediate *K*_S_ values (Figure S9, A–C). Next, the *K*_B(_*_m_*_)_ accumulation along with *K*_S_ + *K*_N_ was examined. We observed that even if *K*_N_ did not increase constantly with *K*_S_, a linear relationship was observed between *K*_B(_*_m_*_)_ and *K*_S_ + *K*_N_ (Figure S9D). This result suggests that *K*_N_ saturation did not affect our results, although the reason for *K*_N_ saturation against *K*_S_ remains unknown.

Figure 2 shows that allele pairs with *m* = 14 to 18 were not observed. This was due to a lack of allelic pairs with intermediate values of *K*_N_ (*K*_N_ = 7 and 8; Figure S10A), whereas *K*_S_ values are continuously observed (Figure S10B). The absence of certain allele pairs may be due to gaps in the database information. Many sequences obtained from the database provided only exon 2 sequences, and there is a clear need for submitting complete *HLA-DRB1* coding sequences to the database in the future.

### Phylogenetic relationships among *HLA-DRB1* alleles

The phylogenetic tree for *DRB* alleles of primates suggested that the group A *DRB1* alleles in humans formed a monophyletic group from other *DRB* alleles. However, the remaining *HLA-DRB1* alleles were polyphyletic. HLA-DRB1*01 and *10 belonged to the same DR1 haplotype, but only those alleles were assigned to different groups (group A and group B in Figure 3). Andersson (1998) showed that the *DR1* haplotype with HLA-DRB1*01 or HLA-DRB1*10 is likely derived from the *DR51* haplotype (HLA-DRB1*15 and HLA-DRB1*16) in group B during recent primate evolution. The exon 2 sequence in HLA-DRB1*10 appears to be similar to that of HLA-DRB1*01 (Gongora *et al.* 1997). However, the configuration of repetitive elements in a segment encompassing the promoter region to upstream of exon 2 (including exon 1) showed higher similarity of HLA-DRB1*10 with that of HLA-DRB1*03 in *DR52* haplotype in group A than with HLA-DRB1*01. The assignment of HLA-DRB1*10 to group A in the non-PBR tree may be affected by conversion of exon 1 and surrounding sequences from HLA-DRB1*03 to HLA-DRB1*10, although the exon 1 sequence was quite short. Because the bootstrap value on the node leading to the group A branch was not high (33%), the clustering of group A still remains a matter of debate.

### The cause of stagnation of *K*_B(_*_m_*_)_ accumulation

A comparison of the relationship between non-PBR and PBR genetic distances on each branch of the non-PBR ML tree showed three allelic lineages (HLA-DRB1*04, *15, and *16) with a slow PBR substitution rate and two lineages (HLA-DRB1*03 and *07) with a fast rate. The allelic lineages with the slow substitution rate were frequently included in phase II. This result suggests that a decline of the PBR substitution rate likely caused the stagnation of *K*_B(_*_m_*_)_ accumulation. Interestingly, although the PBR substitution rate was reduced, most *d*_N_/*d*_S_ values of those branches were higher than unity, suggesting that balancing selection is still active.

The computer simulation supported the finding that the slow-down of amino acid substitution rate in the PBR caused the stagnation of *K*_B(_*_m_*_)_ accumulation. The duration of the tentative plateaus was slightly different between the simulation and that observed. The extent of rate acceleration or deceleration was arbitrary in this simulation and branches with fast or slow rates may have been underestimated due to the strict criteria for heterogeneity identification (see *Materials and Methods*). This probably caused the difference in the timing of plateaus.

In the symmetric balancing selection model, nonsynonymous substitutions at the PBR sites proportionally increased with divergence time and eventually reached a plateau (Takahata *et al.* 1992) (Figure S1). However, in this study *K*_B(_*_m_*_)_ values did not constantly increase with the divergence time of alleles. The theory of asymmetric balancing selection may explain this phenomenon because the fitness of heterozygotes or homozygotes is not equivalent as per this theory. However, asymmetric balancing selection does not fit the model of polymorphism for the actual data in the simulation (Takahata and Nei 1990). Thus, asymmetric balancing selection could not explain the skewed *K*_B(_*_m_*_)_ distribution in the present study.

We used PBR sites identified by Brown *et al.* (1993) to estimate *K*_B(_*_m_*_)_ values. When PBR sites identified by recent assays for higher-order structures of the HLA molecule (S. Kusano and S. Yokoyama, personal communication) were used, the *K*_B_ saturation pattern was also observed in phase II. In addition, allele pairs with low PBR substitution rates were also assigned to phase II. The PBR definitions did not affect our results.

### Biological significance of substitution rate heterogeneity

Slow nucleotide substitution rates, in particular allelic lineages (HLA-DRB1*04, *15, and *16), may be necessary to retain the binding affinity for specific peptides. In general, evolutionary rates of hosts and pathogens have been accelerated by virtue of their arms race. However, HLA molecules preferentially target evolutionarily conserved regions that are functionally important sites for pathogens. Among HLA alleles there appears to be a difference of correlation between the peptide-binding affinity and conservation of the targeted regions (Hertz *et al.* 2011). If this is true, then these slower lineages must be highly specific to certain pathogens, which are the source of these specific peptides.

Using the IEDB database, we examined peptides bound by *DRB1* allelic lineage with the slow PBR substitution rate (HLA-DRB1*04, *15, and *16) and pathogens from which the peptides were derived (Table S3). The prediction from the database showed that one pathogen was specifically recognized by the HLA molecule with a fast PBR substitutions rate, whereas 12 pathogens were specifically recognized by HLA molecules with a slow PBR substitution rate. These more slowly evolving HLA molecules recognized viruses and bacteria that cause disease specific to humans. In addition, HLA-DRB1*04:01 and HLA-DRB1*04:07 bound peptides from lactic bacteria, which are also specific to humans. To cope with these specific pathogens, the nucleotide substitution rate at particular *DRB1* alleles had possibly slowed down relative to those at other alleles. One may hypothesize that evolutionary rates of amino acids at sites 9, 11, 13, and 37 in the HLA-DRB1*04 lineage and at sites 11 and 13 in the DRB1*15/*16 lineages were decelerated, respectively, to retain the affinity of the conserved proteomic region in pathogens (green branches in Figure 5 and Figure S4). According to our estimate, the divergence time of allelic pairs in phase II is 18 MYA to 24 MYA. Humans and the pathogens described above might have coexisted for long periods of time, at least before speciation events of the subfamily Homininae.

In summary, we observed the episodic fluctuations of the amino acid substitution rate in the PBR over the course of *HLA* evolution. This observation was not anticipated because we believed that nonsynonymous substitutions in the PBR increased with the divergence time of the alleles until the substitutions reached saturation. In the case of the *DRB1* gene, such fluctuation is probably due to a decrease of amino acid substitution rates at the PBR on the stem of particular allelic lineages. This suggests that a part of the peptide-binding repertoire at the *HLA-DRB1* locus has been limited over long periods of time due to the recognition of certain pathogens.

## Supplementary Material

Supporting Information

## References

[bib1] Andersson G., 1998 Evolution of the human HLA-DR region. Front. Biosci. 3: d739–d745. 9675159 10.2741/a317

[bib2] Brown J. H. Jardetzky T. S. Gorga J. C. Stern L. J. Urban R. G., 1993 Three-dimensional structure of the human class II histocompatibility antigen HLA-DR1. Nature 364: 33–39. 8316295 10.1038/364033a0

[bib3] Carrington M. Nelson G. W. Martin M. P. Kissner T. Vlahov D., 1999 *HLA* and HIV-1: heterozygote advantage and *B*35-Cw*04* disadvantage. Science 283: 1748–1752. 10073943 10.1126/science.283.5408.1748

[bib4] Demotz S. Barbey C. Corradin G. Amoroso A. Lanzavecchia A., 1993 The set of naturally processed peptides displayed by DR molecules is tuned by polymorphism of residue 86. Eur. J. Immunol. 23: 425–432. 7679644 10.1002/eji.1830230219

[bib5] Doherty P. C. Zinkernagel R. M., 1975 Enhanced immunological surveillance in mice heterozygous at the H-2 gene complex. Nature 256: 50–52. 1079575 10.1038/256050a0

[bib6] Felsenstein J., 2009 PHYLIP (Phylogeny Inference Package) ver.3.69. Distributed by the author, Department of Genome Sciences, University of Washington, Seattle, USA.

[bib7] Gongora R. Figueroa F. Klein J., 1997 Complex origin of the HLA-DR10 haplotype. J. Immunol. 159: 6044–6051. 9550403

[bib8] Gonzalez-Galarza F. F. Christmas S. Middleton D. Jones A. R., 2011 Allele frequency net: a database and online repository for immune gene frequencies in worldwide populations. Nucleic Acids Res. 39: D913–D919. 21062830 10.1093/nar/gkq1128 PMC3013710

[bib9] Gregersen P. K. Shen M. Song Q. L. Merryman P. Degar S., 1986 Molecular diversity of HLA-DR4 haplotypes. Proc. Natl. Acad. Sci. USA 83: 2642–2646. 3458223 10.1073/pnas.83.8.2642 PMC323355

[bib10] Hasegawa M. Kishino H. Yano T., 1985 Dating of the human-ape splitting by a molecular clock of mitochondrial DNA. J. Mol. Evol. 22: 160–174. 3934395 10.1007/BF02101694

[bib11] Hertz T. Nolan D. James I. John M. Gaudieri S., 2011 Mapping the landscape of host-pathogen coevolution: HLA class I binding and its relationship with evolutionary conservation in human and viral proteins. J. Virol. 85: 1310–1321. 21084470 10.1128/JVI.01966-10 PMC3020499

[bib12] Hill A. V. S., 1991 HLA associations with malaria in Africa: some implications for MHC evolution, pp. 403–419 in Molecular evolution of the major histocompatibility complex, edited by Klein J. Klein D. Springer, Berlin, Heidelberg.

[bib13] Hughes A. L. Nei M., 1988 Pattern of nucleotide substitution at major histocompatibility complex class I loci reveals overdominant selection. Nature 335: 167–170. 3412472 10.1038/335167a0

[bib14] Hughes A. L. Nei M., 1989 Nucleotide substitution at major histocompatibility complex class II loci: evidence for overdominant selection. Proc. Natl. Acad. Sci. USA 86: 958–962. 2492668 10.1073/pnas.86.3.958 PMC286598

[bib15] Hughes A. L. Yeager M., 1998 Natural selection at major histocompatibility complex loci of vertebrates. Annu. Rev. Genet. 32: 415–435. 9928486 10.1146/annurev.genet.32.1.415

[bib16] Jones D. Taylor W. Thornton J., 1992 The rapid generation of mutation data matrices from protein sequences. Comput. Appl. Biosci. 8: 275–282. 1633570 10.1093/bioinformatics/8.3.275

[bib17] Klein J. Sato A. Nikolaidis N., 2007 MHC, TSP, and the origin of species: from immunogenetics to evolutionary genetics. Annu. Rev. Genet. 41: 281–304. 18076327 10.1146/annurev.genet.41.110306.130137

[bib18] Kusaba M. Nishio T. Satta Y. Hinata K. Ockendon D., 1997 Striking sequence similarity in inter- and intra-specific comparisons of class I *SLG* alleles from *Brassica oleracea* and *Brassica campestris*: implications for the evolution and recognition mechanism. Proc. Natl. Acad. Sci. USA 94: 7673–7678. 9207151 10.1073/pnas.94.14.7673 PMC23881

[bib19] Musolf K. Meyer-Lucht Y. Sommer S., 2004 Evolution of MHC-*DRB* class II polymorphism in the genus *Apodemus* and a comparison of *DRB* sequences within the family Muridae (Mammalia: Rodentia). Immunogenetics 56: 420–426. 15351919 10.1007/s00251-004-0715-9

[bib20] Nowak M. Tarczy-Hornoch K. Austyn J., 1992 The optimal number of major histocompatibility complex molecules in an individual. Proc. Natl. Acad. Sci. USA 89: 10896–10899. 1438295 10.1073/pnas.89.22.10896 PMC50449

[bib21] Oliver M. K. Telfer S. Piertney S. B., 2009 Major histocompatibility complex (MHC) heterozygote superiority to natural multi-parasite infections in the water vole (*Arvicola terrestris*). Proc. Biol. Sci. 276: 1119–1128. 19129114 10.1098/rspb.2008.1525 PMC2679068

[bib22] Ong B. Willcox N. Wordsworth P. Beeson D. Vincent A., 1991 Critical role for the Val/Gly^86^ HLA-DR*β* dimorphism in autoantigen presentation to human T cells. Proc. Natl. Acad. Sci. USA 88: 7343–7347. 1714600 10.1073/pnas.88.16.7343 PMC52291

[bib23] Penn D. J. Damjanovich K. Potts W. K., 2002 MHC heterozygosity confers a selective advantage against multiple-strain infections. Proc. Natl. Acad. Sci. USA 99: 11260–11264. 12177415 10.1073/pnas.162006499 PMC123244

[bib24] Reche P. A. Reinherz E. L., 2003 Sequence variability analysis of human class I and class II MHC molecules: Functional and structural correlates of amino acid polymorphisms. J. Mol. Biol. 331: 623–641. 12899833 10.1016/s0022-2836(03)00750-2

[bib25] Robinson J. Waller M. J. Stoehr P. Marsh S. G. E., 2005 IPD-the Immuno Polymorphism Database. Nucleic Acids Res. 33: D523–D526. 15608253 10.1093/nar/gki032 PMC539986

[bib26] Robinson J. Mistry K. McWilliam H. Lopez R. Parham P., 2011 The IMGT/HLA database. Nucleic Acids Res. 39: D1171–D1176. 21071412 10.1093/nar/gkq998 PMC3013815

[bib27] Saitou N. Nei M., 1987 The neighbor-joining method: a new method for reconstructing phylogenetic trees. Mol. Biol. Evol. 4: 406–425. 3447015 10.1093/oxfordjournals.molbev.a040454

[bib28] Satta, Y., 1992 Balancing selection at HLA loci, pp. 111–131 in *The Proceedings of the 17th Taniguchi Symposium*, edited by N. Takahata. Japan Science Society Press, Tokyo.

[bib29] Satta Y., C. O’hUigin, N. Takahata, and J. Klein., 1994 Intensity of natural selection at the major histocompatibility complex loci. Proc. Natl. Acad. Sci. USA 91: 7184–7188. 8041766 10.1073/pnas.91.15.7184 PMC44363

[bib30] Sawyer S., 1989 Statistical tests for detecting gene conversion. Mol. Biol. Evol. 6: 526–538. 2677599 10.1093/oxfordjournals.molbev.a040567

[bib31] Slade R. W. McCallum H. I., 1992 Overdominant *vs.* frequency-dependent selection at MHC loci. Genetics 132: 861–862. 1468635 10.1093/genetics/132.3.861 PMC1205221

[bib32] Sommer S., 2005 The importance of immune gene variability (MHC) in evolutionary ecology and conservation. Front. Zool. 2: 16. 16242022 10.1186/1742-9994-2-16 PMC1282567

[bib33] Spurgin L. G. Richardson D. S., 2010 How pathogens drive genetic diversity: MHC, mechanisms and misunderstandings. Proc. Biol. Sci. 277: 979–988. 20071384 10.1098/rspb.2009.2084 PMC2842774

[bib34] Stern L. J. Brown J. H. Jardetzky T. S. Gorga J. C. Urban R. G., 1994 Crystal structure of the human class II MHC protein HLA-DR1 complexed with an influenza virus peptide. Nature 368: 215–221. 8145819 10.1038/368215a0

[bib35] Takahata N., 1990 A simple genealogical structure of strongly balanced allelic lines and trans-species evolution of polymorphism. Proc. Natl. Acad. Sci. USA 87: 2419–2423. 2320564 10.1073/pnas.87.7.2419 PMC53700

[bib36] Takahata N. Nei M., 1990 Allelic genealogy under overdominant and frequency-dependent selection and polymorphism of major histocompatibility complex loci. Genetics 124: 967–978. 2323559 10.1093/genetics/124.4.967 PMC1203987

[bib37] Takahata N. Tajima F., 1991 Sampling Errors in Phylogeny. 8: 494–502.

[bib38] Takahata N. Satta Y. Klein J., 1992 Polymorphism and balancing selection at major histocompatibility complex loci. Genetics 130: 925–938. 1582567 10.1093/genetics/130.4.925 PMC1204941

[bib39] Tamura K. Peterson D. Peterson N. Stecher G. Nei M., 2011 MEGA5: molecular evolutionary genetics analysis using maximum likelihood, evolutionary distance, and maximum parsimony methods. Mol. Biol. Evol. 28: 2731–2739. 21546353 10.1093/molbev/msr121 PMC3203626

[bib40] Thursz M. R. Thomas H. C. Greenwood B. M. Hill A. V., 1997 Heterozygote advantage for HLA class-II type in hepatitis B virus infection. Nat. Genet. 17: 11–12. 9288086 10.1038/ng0997-11

[bib41] Verreck F. A. van de Poel A. Drijfhout J. W. Amons R. Coligan J. E., 1996 Natural peptides isolated from Gly86/Val86-containing variants of HLA-DR1, -DR11, -DR13, and -DR52. Immunogenetics 43: 392–397. 8606061 10.1007/BF02199809

[bib42] Vidović D. Matzinger P., 1988 Unresponsiveness to a foreign antigen can be caused by self-tolerance. Nature 336: 222–225. 3143074 10.1038/336222a0

[bib43] Vita R. Zarebski L. Greenbaum J. A. Emami H. Hoof I., 2010 The immune epitope database 2.0. Nucleic Acids Res. 38: D854–D862. 19906713 10.1093/nar/gkp1004 PMC2808938

[bib44] Woelfing B. Traulsen A. Milinski M. Boehm T., 2009 Does intra-individual major histocompatibility complex diversity keep a golden mean? Philos. Trans. R. Soc. Lond. B Biol. Sci. 364: 117–128. 18926972 10.1098/rstb.2008.0174 PMC2666699

