# Supplementary material for: Nonsynonymous Substitution Rate Heterogeneity in the Peptide-Binding Region Among Different HLA-DRB1 Lineages in Humans
Source: G3 (Bethesda). 2014 May 2;4(7):1217–26. doi: 10.1534/g3.114.011726 (PMC4455771; doi:10.1534/g3.114.011726)
Supplement: Supporting Information [file supp_4_7_1217__index.html]

Nonsynonymous Substitution Rate Heterogeneity in the Peptide-Binding Region Among Different HLA-DRB1 Lineages in Humans — Supporting Information 

# Nonsynonymous Substitution Rate Heterogeneity in the Peptide-Binding Region Among Different *HLA-DRB1* Lineages in Humans

## Supporting Information for Yasukochi and Satta, 2014

**Files in this Data Supplement:**

- Supporting Information - Figures S1-S10 and Tables S1-S3 (PDF, 302 KB)
- Figure S1 - Expected distribution of *K*B(*m*) values. (PDF, 155 KB)
- Figure S2 - The relationship of level of amino acid substitutions at the PBR (*K*B(*m*)) and coalescence time (*m* = *K*S) of alleles for 24 rat *RT1-Db1* (*HLA-DRB1* ortholog) alleles. (PDF, 149 KB)
- Figure S3 - Neighbor-joining (NJ) and maximum likelihood (ML) trees based on nucleotide and amino acid sequences in the non-PBRs of *HLA-DRB1* alleles. (PDF, 168 KB)
- Figure S4 - Maximum likelihood tree with the HKY model based on nucleotide sequences (690 bp) in the non-PBRs of *HLA-DRB1* alleles. (PDF, 164 KB)
- Figure S5 - The number of transition and transversion substitutions at the PBR in *HLA-DRB1* alleles. (PDF, 129 KB)
- Figure S6 - The level of amino acid substitutions at the PBR (*K*B(*m*)) among *HLA-DRB1* allele pairs that share the same coalescence time (*m* = *K*S + *K*N) in a Japanese population. (PDF, 151 KB)
- Figure S7 - The level of amino acid substitutions at the PBR (*K*B(*m*)) among *HLA-DRB1* allele pairs that share the same coalescence time (*m* = *K*S + *K*N) in 45 non-recombinant alleles. (PDF, 151 KB)
- Figure S8 - The level of amino acid substitutions at the non-PBR (*K*N(*m*)) among *HLA-DRB1* allele pairs that share the same coalescence time (*m* = *K*S). (PDF, 148 KB)
- Figure S9 - Relationships among *K*S, *K*N and *K*B. (PDF, 143 KB)
- Figure S10 - The mean number of nonsynonymous substitutions at the PBR (*K*B(*m*)) among *HLA-DRB1* allele pairs that share the same *K*N and *K*S values. (PDF, 177 KB)
- Table S1 - The allelic pairs consisting of phase II in the *HLA-DRB1* locus. (PDF, 137 KB)
- Table S2 - Parallel substitution sites in the PBR among allele pairs over all phases. (PDF, 118 KB)
- Table S3 - The specific pathogens bound by HLA-DRB1 allelic lineages only with fast or slow PBR substitution rate. (PDF, 127 KB)
