# Supplementary material for: Nonsynonymous Substitution Rate Heterogeneity in the Peptide-Binding Region Among Different HLA-DRB1 Lineages in Humans
Source: G3 (Bethesda). 2014 May 2;4(7):1217–26. doi: 10.1534/g3.114.011726 (PMC4455771; doi:10.1534/g3.114.011726)
Supplement: Supporting Information [file supp_g3.114.011726_011726SI.pdf]

G3: Genes | Genomes | Genetics

Nonsynonymous Substitution Rate Heterogeneity in the Peptide-Binding Region among Different *HLA-DRB1* Lineages in Humans

Yoshiki Yasukochi[*,1,2], Yoko Satta[§]

*Molecular and Genetic Epidemiology, Faculty of Medicine, University of Tsukuba, Tsukuba, Ibaraki 305-8575, Japan, and [§] Department of Evolutionary Studies of Biosystems, the Graduate University for Advanced Studies (SOKENDAI), Hayama, Kanagawa 240-0193, Japan

[2]Corresponding author: Yoshiki Yasukochi

DOI: 10.1534/g3.114.011726

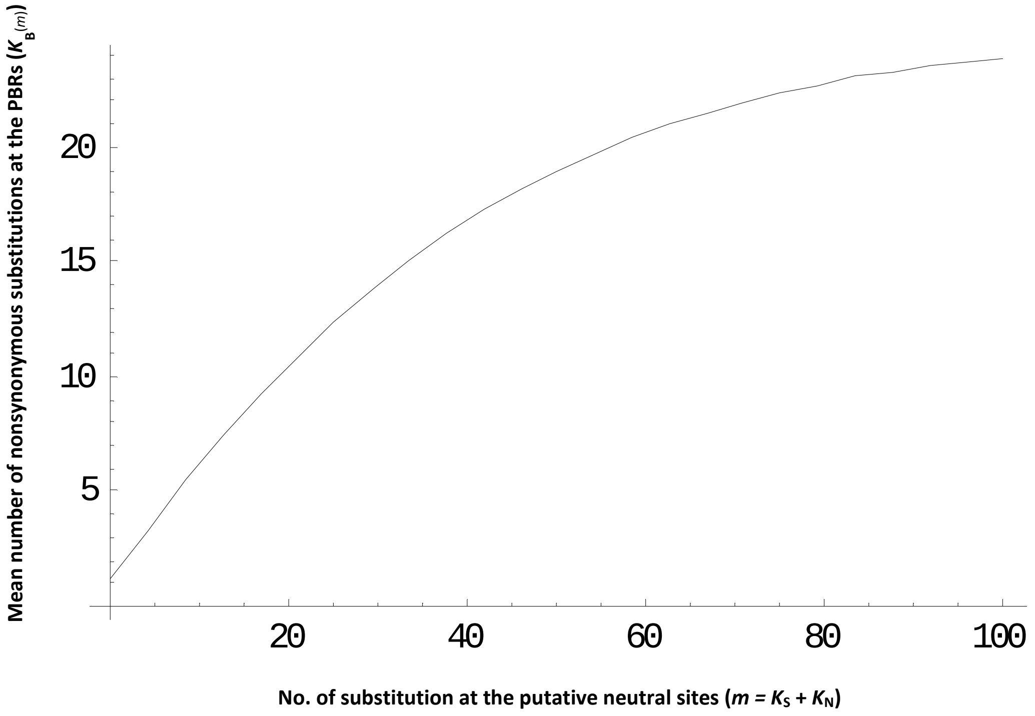

Figure S1 Expected distribution of $K_{B(m)}$ values. The pairwise mean number of nonsynonymous substitutions $K_{B(m)}$ in the PBR conditioned on the number of putative synonymous substitutions *m*. This conditional mean number is calculated on the basis of Equation 12 described in Takahata *et al*. (1992).

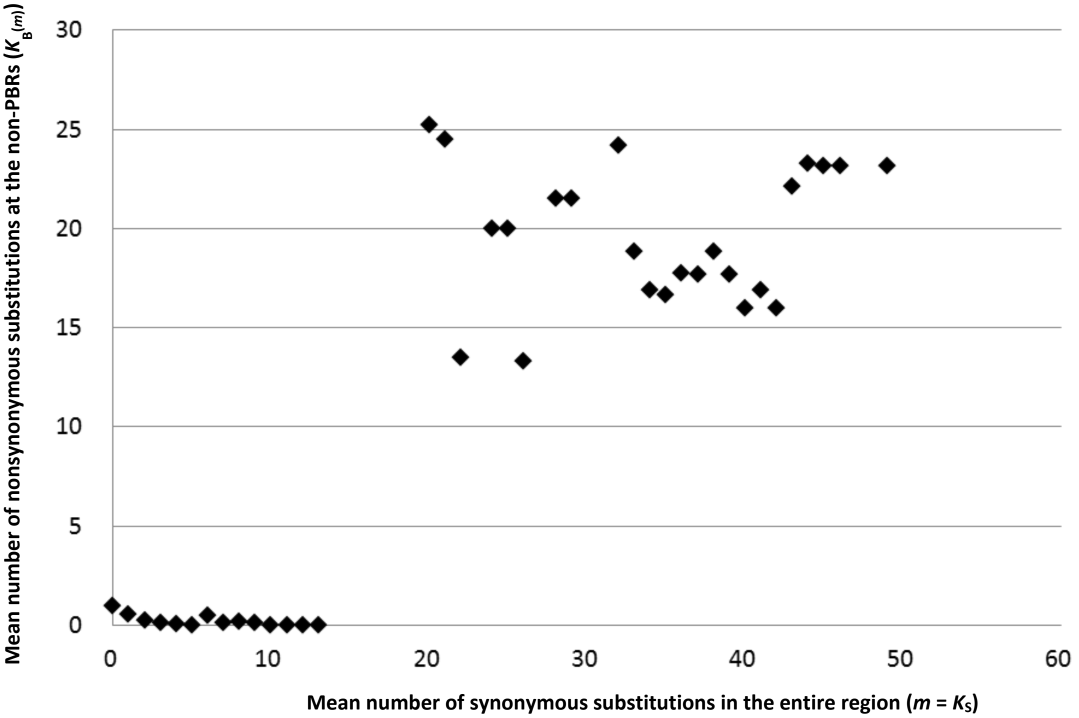

Figure S2 The relationship of level of amino acid substitutions at the PBR ($K_{B(m)}$) and coalescence time ($m = K_S$) of alleles for 24 rat *RT1-Db1* (*HLA-DRB1* ortholog) alleles. The ordinate axis represents the mean number of nonsynonymous substitutions at the PBR among allele pairs ($K_{B(m)}$). The abscissa axis represents the number of synonymous substitutions over the entire region ($m = K_S$).

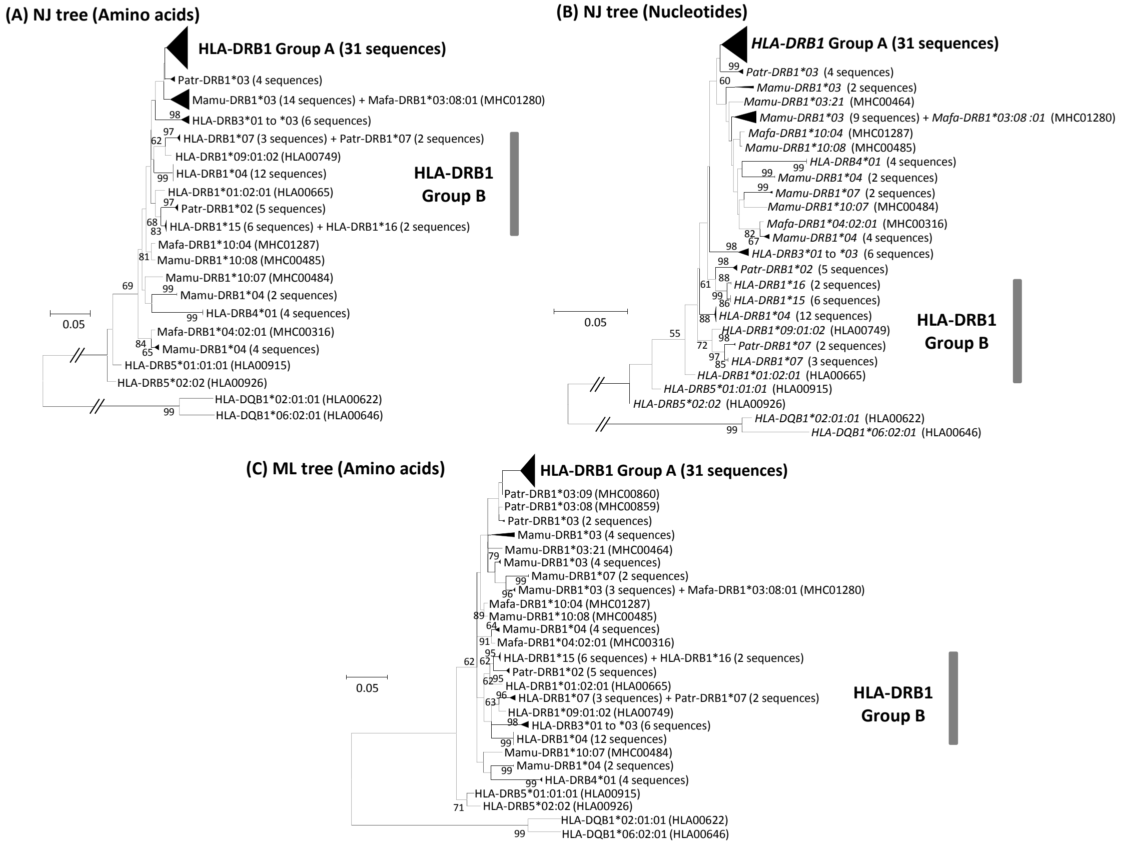

Figure S3 Neighbor-joining (NJ) and maximum likelihood (ML) trees based on nucleotide and amino acid sequences in the non-PBRs of *HLA-DRB1* alleles. (A) NJ tree of amino acid sequences of *DRB1* alleles on the basis of the JTT model with gamma distribution, (B) NJ tree of nucleotide sequences on the basis of the Tamura 3 parameters model with gamma distribution, (C) ML tree of amino acid sequences on the basis of the JTT model. The tree construction and estimation of the best fit substitution model were performed by using the MEGA v5.10 software. Only bootstrap values over 50% are shown in this figure. Two *HLA-DQB1* sequences are used as the outgroup. *HLA*, humans; *Patr*, chimpanzees; *Mamu*, rhesus monkeys; *Mafa*, crab-eating macaques. IMGT/HLA and IPD Accession Numbers are in parentheses.

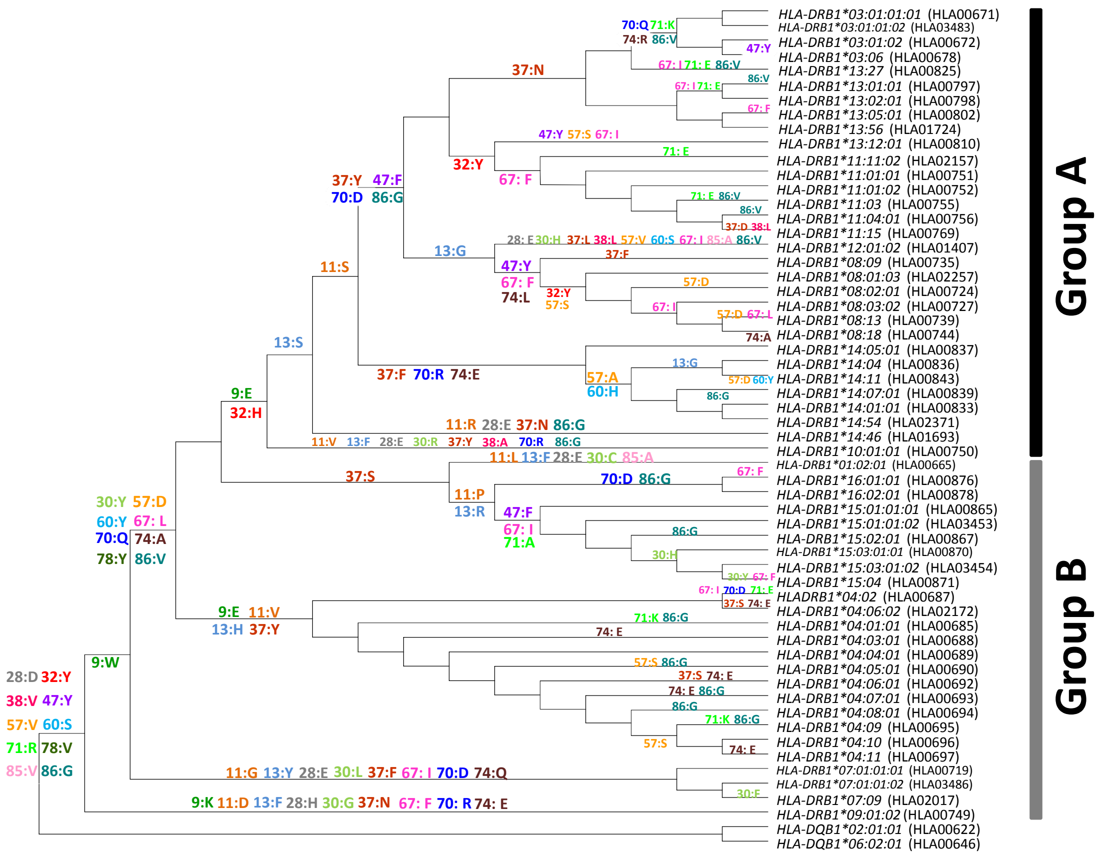

Figure S4 Maximum likelihood tree with the HKY model based on nucleotide sequences (690 bp) in the non-PBRs of *HLA-DRB1* alleles. Characters on branches indicate amino acid at each PBR position. Arrows represent the shared same amino acids from the node to tips. IMGT/HLA Accession Numbers are in parentheses.

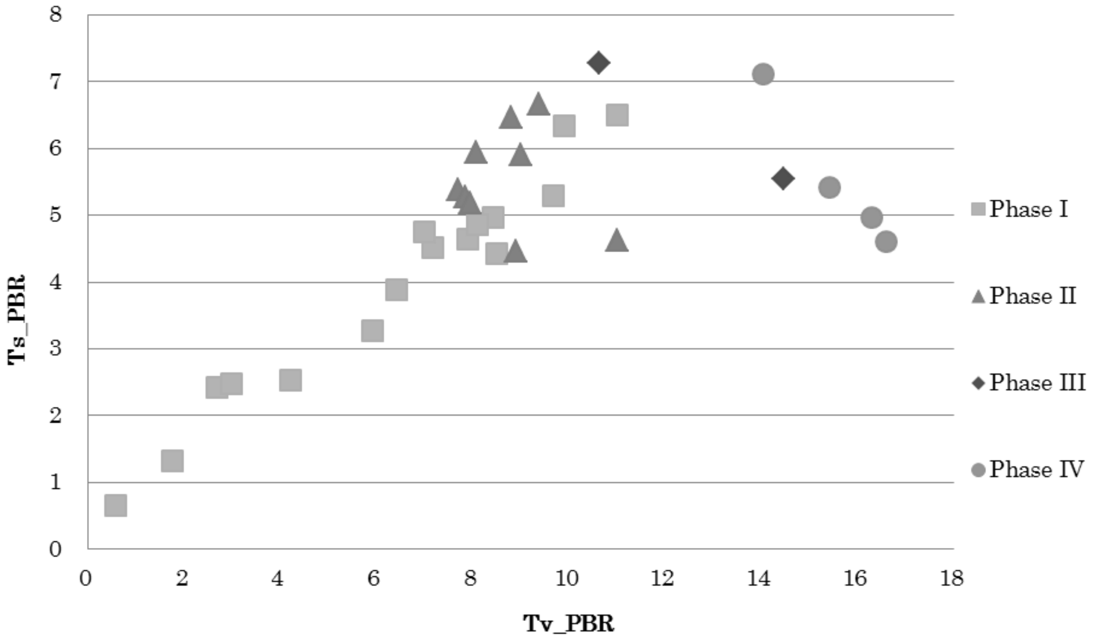

Figure S5 The number of transition and transversion substitutions at the PBR in *HLA-DRB1* alleles. The abscissa axis represents the mean number of transition substitutions at the PBR among allele pairs. The ordinate axis represents the mean number of transversion substitutions at the PBR.

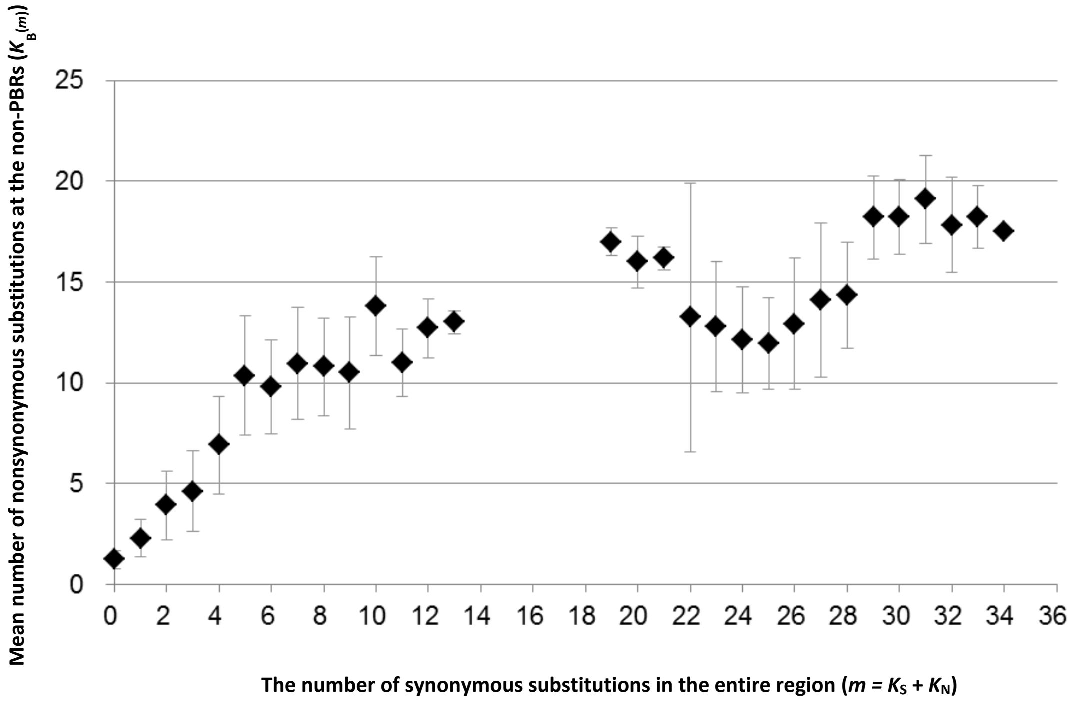

Figure S6 The level of amino acid substitutions at the PBR ($K_{B(m)}$) among *HLA-DRB1* allele pairs that share the same coalescence time ($m = K_S + K_N$) in a Japanese population. The ordinate axis represents the mean number of nonsynonymous substitutions at the non-PBR among allele pairs ($K_{B(m)}$). The abscissa axis represents the number of putative synonymous substitutions ($m = K_S + K_N$). Error bars indicate the standard deviation from the mean. The $K_{B(m)}$ and m values were calculated by using 30 non-recombinant alleles observed in a Japanese population.

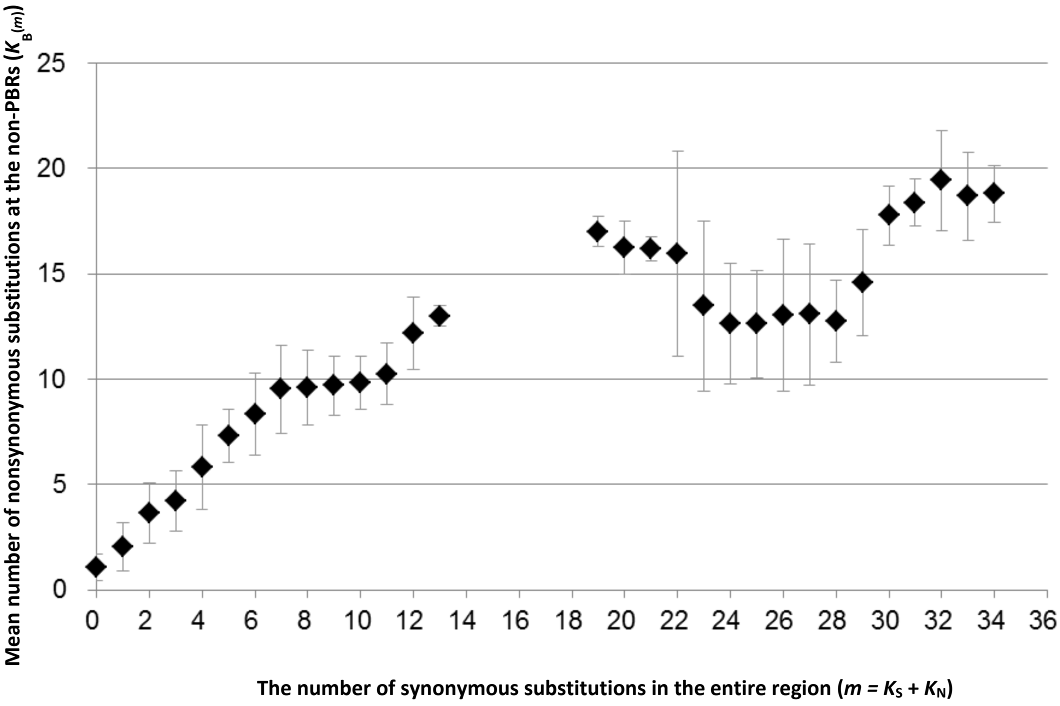

Figure S7 The level of amino acid substitutions at the PBR ($K_{B(m)}$) among *HLA-DRB1* allele pairs that share the same coalescence time ($m = K_S + K_N$) in 45 non-recombinant alleles. The ordinate axis represents the mean number of nonsynonymous substitutions at the non-PBR among allele pairs ($K_{B(m)}$). The abscissa axis represents the number of putative synonymous substitutions ($m = K_S + K_N$). Error bars indicate the standard deviation from the mean. The $K_{B(m)}$ and m values were calculated by using 45 non-recombinant alleles which were not showed as a recombinant by both of the method described in Satta (1992) and GENECONV program.

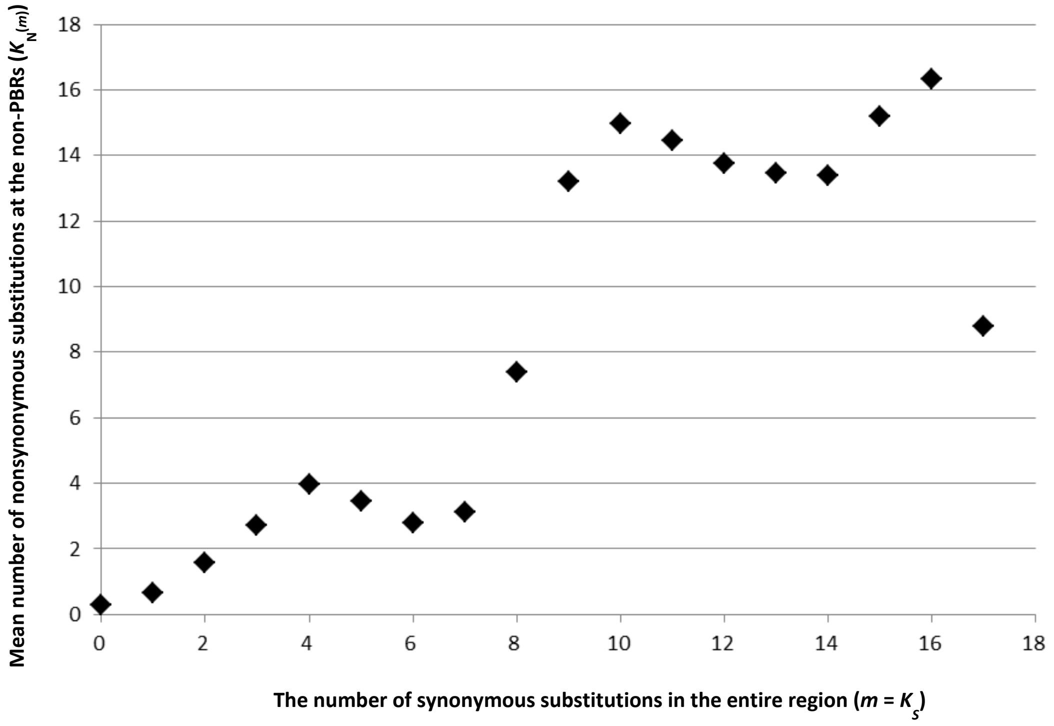

Figure S8 The level of amino acid substitutions at the non-PBR ($K_{N(m)}$) among *HLA-DRB1* allele pairs that share the same coalescence time ($m = K_S$). The ordinate axis represents the mean number of nonsynonymous substitutions at the non-PBR among allele pairs ($K_{N(m)}$). The abscissa axis represents the number of synonymous substitutions over the entire region ($m = K_S$).

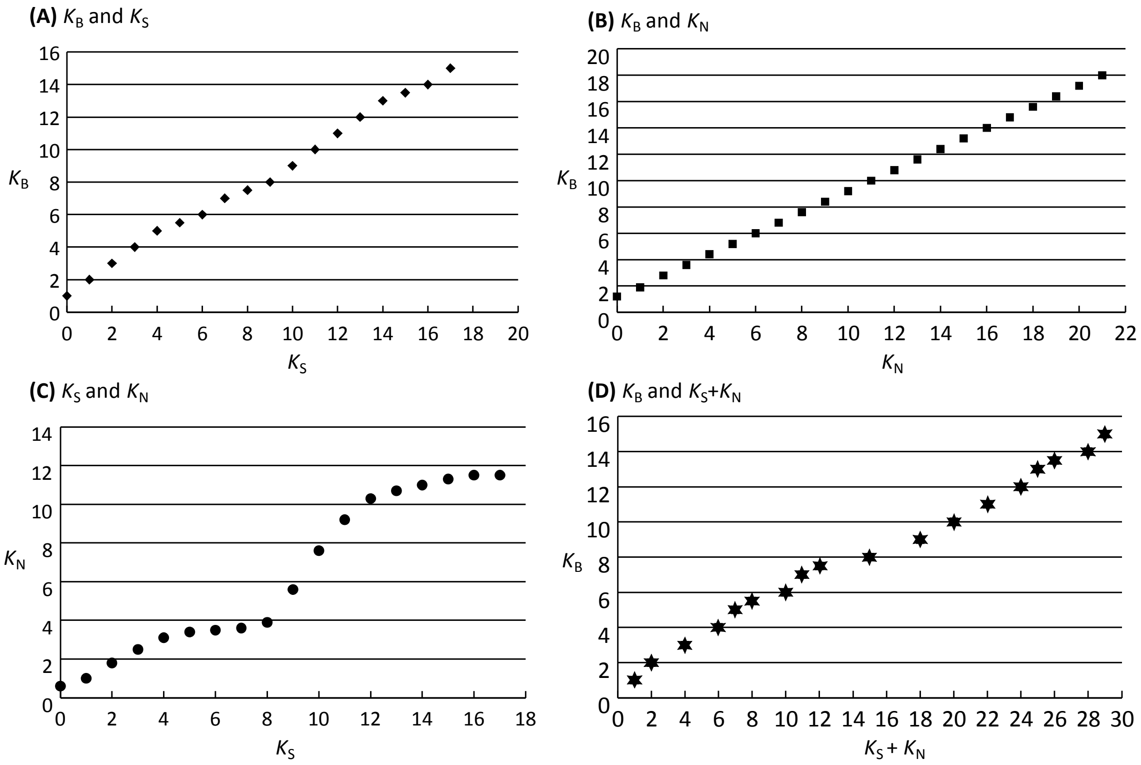

Figure S9 Relationships among K_S, K_N and K_B (A) Relationship between K_B and K_S, (B) Relationship between K_B and K_N, (C) Relationship between K_S and K_N, (D) Relationship between K_B and $K_S + K_N$. Even though K_N does not increase constantly with K_S, a proportional relationship was observed between K_B and $K_S + K_N$.

(A)

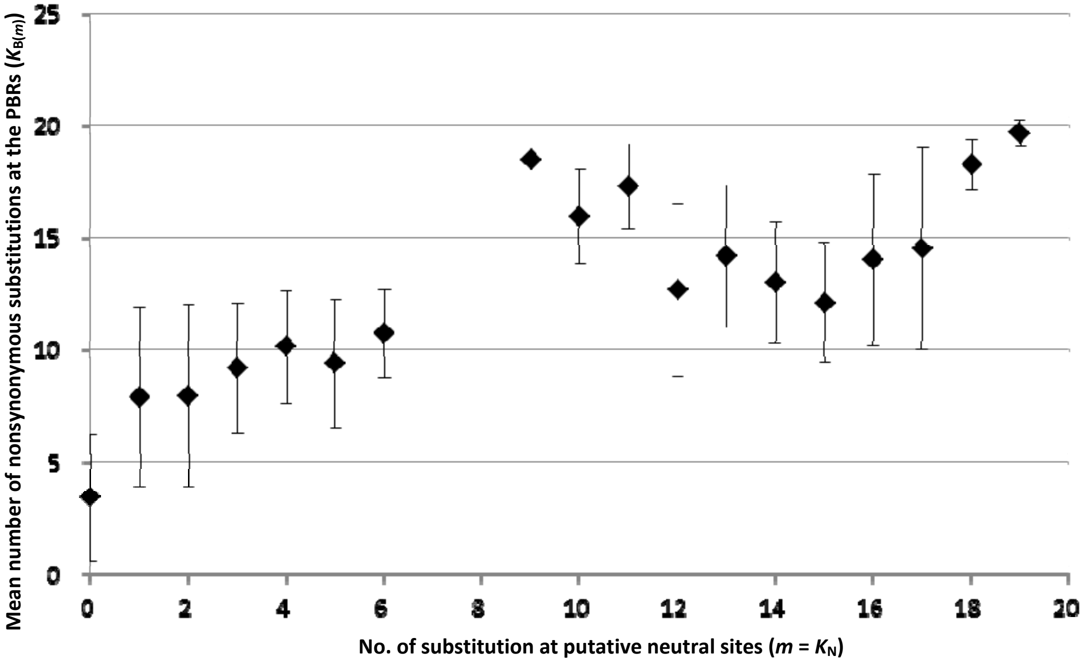

(B)

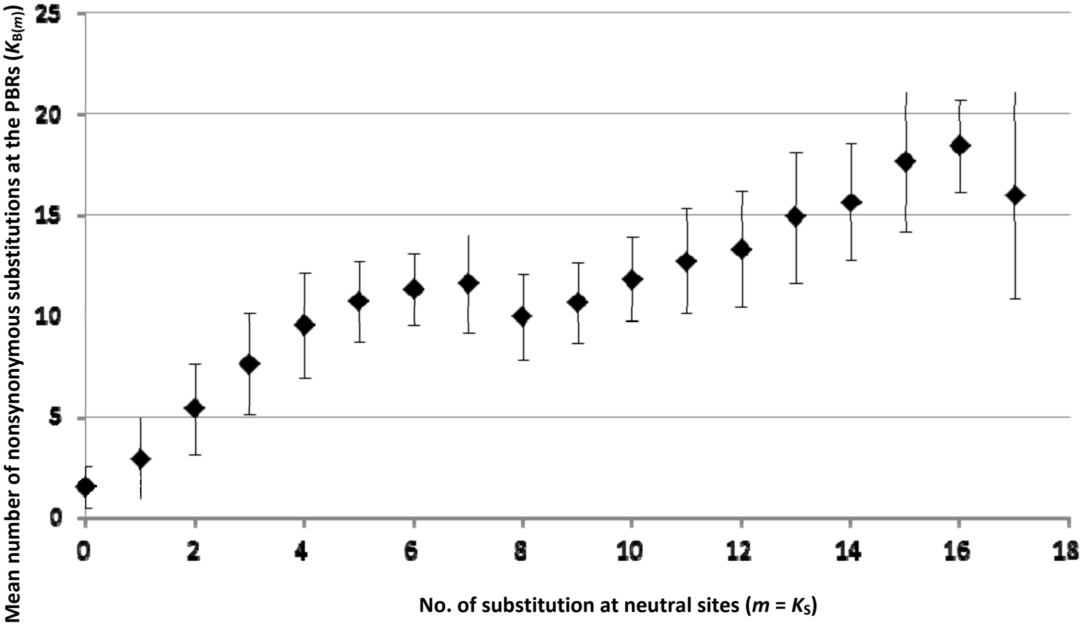

Figure S10 The mean number of nonsynonymous substitutions at the PBR ($K_{B(m)}$) among *HLA-DRB1* allele pairs that share the same K_N and K_S values. The ordinate axis represents the mean number of nonsynonymous substitutions at the PBR ($K_{B(m)}$). Abscissa axes represent K_N values (A) and K_S values (B). Error bars indicate the standard deviation from the mean.

Table S1 The allelic pairs consisting of phase II in the *HLA-DRB1* locus

Allele 1	Allele 2	*m*	K_B
HLA:HLA00685 DRB1*04:01:01	HLA:HLA00749 DRB1*09:01:02	20	17
HLA:HLA00693 DRB1*04:07:01	HLA:HLA00749 DRB1*09:01:02	20	15
HLA:HLA00694 DRB1*04:08:01	HLA:HLA00749 DRB1*09:01:02	20	16
HLA:HLA00696 DRB1*04:10	HLA:HLA00749 DRB1*09:01:02	20	18
HLA:HLA00697 DRB1*04:11	HLA:HLA00749 DRB1*09:01:02	20	17
HLA:HLA00665 DRB1*01:02:01	HLA:HLA00688 DRB1*04:03:01	20	10
HLA:HLA00665 DRB1*01:02:01	HLA:HLA00689 DRB1*04:04:01	20	9
HLA:HLA00665 DRB1*01:02:01	HLA:HLA00692 DRB1*04:06:01	20	9
HLA:HLA00685 DRB1*04:01:01	HLA:HLA01693 DRB1*14:46	20	8
HLA:HLA00693 DRB1*04:07:01	HLA:HLA01693 DRB1*14:46	20	8
HLA:HLA00694 DRB1*04:08:01	HLA:HLA01693 DRB1*14:46	20	7
HLA:HLA00665 DRB1*01:02:01	HLA:HLA00865 DRB1*15:01:01:01	20	11
HLA:HLA00665 DRB1*01:02:01	HLA:HLA03453 DRB1*15:01:01:02	20	11
HLA:HLA00665 DRB1*01:02:01	HLA:HLA00870 DRB1*15:03:01:01	20	12
HLA:HLA00665 DRB1*01:02:01	HLA:HLA03454 DRB1*15:03:01:02	20	12
HLA:HLA00665 DRB1*01:02:01	HLA:HLA00871 DRB1*15:04	20	11
HLA:HLA00688 DRB1*04:03:01	HLA:HLA00749 DRB1*09:01:02	21	16
HLA:HLA00689 DRB1*04:04:01	HLA:HLA00749 DRB1*09:01:02	21	17
HLA:HLA00692 DRB1*04:06:01	HLA:HLA00749 DRB1*09:01:02	21	17
HLA:HLA01693 DRB1*14:46	HLA:HLA00867 DRB1*15:02:01	21	14
HLA:HLA00665 DRB1*01:02:01	HLA:HLA00685 DRB1*04:01:01	21	11
HLA:HLA00665 DRB1*01:02:01	HLA:HLA00687 DRB1*04:02	21	14
HLA:HLA00665 DRB1*01:02:01	HLA:HLA02172 DRB1*04:06:02	21	9
HLA:HLA00665 DRB1*01:02:01	HLA:HLA00693 DRB1*04:07:01	21	11
HLA:HLA00665 DRB1*01:02:01	HLA:HLA00694 DRB1*04:08:01	21	10
HLA:HLA00665 DRB1*01:02:01	HLA:HLA00696 DRB1*04:10	21	11
HLA:HLA00665 DRB1*01:02:01	HLA:HLA00697 DRB1*04:11	21	12
HLA:HLA00688 DRB1*04:03:01	HLA:HLA01693 DRB1*14:46	21	9
HLA:HLA00689 DRB1*04:04:01	HLA:HLA01693 DRB1*14:46	21	8
HLA:HLA00690 DRB1*04:05:01	HLA:HLA01693 DRB1*14:46	21	9
HLA:HLA00692 DRB1*04:06:01	HLA:HLA01693 DRB1*14:46	21	10
HLA:HLA00695 DRB1*04:09	HLA:HLA01693 DRB1*14:46	21	10
HLA:HLA00750 DRB1*10:01:01	HLA:HLA01693 DRB1*14:46	21	8
HLA:HLA00665 DRB1*01:02:01	HLA:HLA00867 DRB1*15:02:01	21	12
HLA:HLA00690 DRB1*04:05:01	HLA:HLA00719 DRB1*07:01:01:01	22	19
HLA:HLA00695 DRB1*04:09	HLA:HLA00719 DRB1*07:01:01:01	22	20
HLA:HLA00690 DRB1*04:05:01	HLA:HLA03486 DRB1*07:01:01:02	22	19
HLA:HLA00695 DRB1*04:09	HLA:HLA03486 DRB1*07:01:01:02	22	20
HLA:HLA00690 DRB1*04:05:01	HLA:HLA02017 DRB1*07:09	22	18
HLA:HLA00695 DRB1*04:09	HLA:HLA02017 DRB1*07:09	22	19
HLA:HLA00665 DRB1*01:02:01	HLA:HLA00749 DRB1*09:01:02	22	19
HLA:HLA02172 DRB1*04:06:02	HLA:HLA00749 DRB1*09:01:02	22	17
HLA:HLA01693 DRB1*14:46	HLA:HLA00865 DRB1*15:01:01:01	22	15
HLA:HLA01693 DRB1*14:46	HLA:HLA03453 DRB1*15:01:01:02	22	15
HLA:HLA01693 DRB1*14:46	HLA:HLA00870 DRB1*15:03:01:01	22	16
HLA:HLA01693 DRB1*14:46	HLA:HLA03454 DRB1*15:03:01:02	22	16
HLA:HLA01693 DRB1*14:46	HLA:HLA00871 DRB1*15:04	22	15
HLA:HLA01693 DRB1*14:46	HLA:HLA00876 DRB1*16:01:01	22	13
HLA:HLA01693 DRB1*14:46	HLA:HLA00878 DRB1*16:02:01	22	12
HLA:HLA00665 DRB1*01:02:01	HLA:HLA00690 DRB1*04:05:01	22	12
HLA:HLA00665 DRB1*01:02:01	HLA:HLA00695 DRB1*04:09	22	13
HLA:HLA00687 DRB1*04:02	HLA:HLA00749 DRB1*09:01:02	22	20
HLA:HLA00687 DRB1*04:02	HLA:HLA00797 DRB1*13:01:01	22	7
HLA:HLA00690 DRB1*04:05:01	HLA:HLA00810 DRB1*13:12:01	22	7
HLA:HLA00695 DRB1*04:09	HLA:HLA00810 DRB1*13:12:01	22	8
HLA:HLA00687 DRB1*04:02	HLA:HLA01693 DRB1*14:46	22	13
HLA:HLA02172 DRB1*04:06:02	HLA:HLA01693 DRB1*14:46	22	10
HLA:HLA00696 DRB1*04:10	HLA:HLA01693 DRB1*14:46	22	10
HLA:HLA00697 DRB1*04:11	HLA:HLA01693 DRB1*14:46	22	11
HLA:HLA00665 DRB1*01:02:01	HLA:HLA00876 DRB1*16:01:01	22	11
HLA:HLA00665 DRB1*01:02:01	HLA:HLA00878 DRB1*16:02:01	22	10
HLA:HLA00685 DRB1*04:01:01	HLA:HLA00719 DRB1*07:01:01:01	23	19
HLA:HLA00693 DRB1*04:07:01	HLA:HLA00719 DRB1*07:01:01:01	23	17
HLA:HLA00694 DRB1*04:08:01	HLA:HLA00719 DRB1*07:01:01:01	23	18
HLA:HLA00696 DRB1*04:10	HLA:HLA00719 DRB1*07:01:01:01	23	20
HLA:HLA00697 DRB1*04:11	HLA:HLA00719 DRB1*07:01:01:01	23	19
HLA:HLA00685 DRB1*04:01:01	HLA:HLA03486 DRB1*07:01:01:02	23	19
HLA:HLA00693 DRB1*04:07:01	HLA:HLA03486 DRB1*07:01:01:02	23	17
HLA:HLA00694 DRB1*04:08:01	HLA:HLA03486 DRB1*07:01:01:02	23	18
HLA:HLA00696 DRB1*04:10	HLA:HLA03486 DRB1*07:01:01:02	23	20
HLA:HLA00697 DRB1*04:11	HLA:HLA03486 DRB1*07:01:01:02	23	19
HLA:HLA00685 DRB1*04:01:01	HLA:HLA02017 DRB1*07:09	23	18
HLA:HLA00693 DRB1*04:07:01	HLA:HLA02017 DRB1*07:09	23	16
HLA:HLA00694 DRB1*04:08:01	HLA:HLA02017 DRB1*07:09	23	17
HLA:HLA00696 DRB1*04:10	HLA:HLA02017 DRB1*07:09	23	19
HLA:HLA00697 DRB1*04:11	HLA:HLA02017 DRB1*07:09	23	18
HLA:HLA00750 DRB1*10:01:01	HLA:HLA00798 DRB1*13:02:01	23	15
HLA:HLA00750 DRB1*10:01:01	HLA:HLA00802 DRB1*13:05:01	23	13
HLA:HLA00750 DRB1*10:01:01	HLA:HLA00810 DRB1*13:12:01	23	14
HLA:HLA00750 DRB1*10:01:01	HLA:HLA01724 DRB1*13:56	23	12
HLA:HLA00798 DRB1*13:02:01	HLA:HLA00876 DRB1*16:01:01	23	13
HLA:HLA00802 DRB1*13:05:01	HLA:HLA00876 DRB1*16:01:01	23	10
HLA:HLA00810 DRB1*13:12:01	HLA:HLA00876 DRB1*16:01:01	23	10
HLA:HLA01724 DRB1*13:56	HLA:HLA00876 DRB1*16:01:01	23	11
HLA:HLA00798 DRB1*13:02:01	HLA:HLA00878 DRB1*16:02:01	23	13
HLA:HLA00802 DRB1*13:05:01	HLA:HLA00878 DRB1*16:02:01	23	11
HLA:HLA00810 DRB1*13:12:01	HLA:HLA00878 DRB1*16:02:01	23	10
HLA:HLA01724 DRB1*13:56	HLA:HLA00878 DRB1*16:02:01	23	10
HLA:HLA00687 DRB1*04:02	HLA:HLA00755 DRB1*11:03	23	6
HLA:HLA00687 DRB1*04:02	HLA:HLA00756 DRB1*11:04:01	23	8
HLA:HLA00685 DRB1*04:01:01	HLA:HLA02157 DRB1*11:11:02	23	9
HLA:HLA00687 DRB1*04:02	HLA:HLA02157 DRB1*11:11:02	23	7
HLA:HLA00693 DRB1*04:07:01	HLA:HLA02157 DRB1*11:11:02	23	11
HLA:HLA00694 DRB1*04:08:01	HLA:HLA02157 DRB1*11:11:02	23	10
HLA:HLA00688 DRB1*04:03:01	HLA:HLA00797 DRB1*13:01:01	23	13
HLA:HLA00689 DRB1*04:04:01	HLA:HLA00797 DRB1*13:01:01	23	12
HLA:HLA00692 DRB1*04:06:01	HLA:HLA00797 DRB1*13:01:01	23	14
HLA:HLA00685 DRB1*04:01:01	HLA:HLA00798 DRB1*13:02:01	23	11
HLA:HLA00687 DRB1*04:02	HLA:HLA00798 DRB1*13:02:01	23	8
HLA:HLA00693 DRB1*04:07:01	HLA:HLA00798 DRB1*13:02:01	23	13
HLA:HLA00694 DRB1*04:08:01	HLA:HLA00798 DRB1*13:02:01	23	12
HLA:HLA00685 DRB1*04:01:01	HLA:HLA00802 DRB1*13:05:01	23	11
HLA:HLA00687 DRB1*04:02	HLA:HLA00802 DRB1*13:05:01	23	11
HLA:HLA00693 DRB1*04:07:01	HLA:HLA00802 DRB1*13:05:01	23	11
HLA:HLA00694 DRB1*04:08:01	HLA:HLA00802 DRB1*13:05:01	23	10
HLA:HLA00685 DRB1*04:01:01	HLA:HLA00810 DRB1*13:12:01	23	10
HLA:HLA00687 DRB1*04:02	HLA:HLA00810 DRB1*13:12:01	23	9
HLA:HLA00693 DRB1*04:07:01	HLA:HLA00810 DRB1*13:12:01	23	10
HLA:HLA00694 DRB1*04:08:01	HLA:HLA00810 DRB1*13:12:01	23	9
HLA:HLA00696 DRB1*04:10	HLA:HLA00810 DRB1*13:12:01	23	8
HLA:HLA00697 DRB1*04:11	HLA:HLA00810 DRB1*13:12:01	23	9
HLA:HLA00685 DRB1*04:01:01	HLA:HLA01724 DRB1*13:56	23	10
HLA:HLA00687 DRB1*04:02	HLA:HLA01724 DRB1*13:56	23	11
HLA:HLA00693 DRB1*04:07:01	HLA:HLA01724 DRB1*13:56	23	10
HLA:HLA00694 DRB1*04:08:01	HLA:HLA01724 DRB1*13:56	23	9
HLA:HLA00688 DRB1*04:03:01	HLA:HLA00719 DRB1*07:01:01:01	24	18
HLA:HLA00689 DRB1*04:04:01	HLA:HLA00719 DRB1*07:01:01:01	24	19
HLA:HLA00692 DRB1*04:06:01	HLA:HLA00719 DRB1*07:01:01:01	24	18
HLA:HLA00688 DRB1*04:03:01	HLA:HLA03486 DRB1*07:01:01:02	24	18
HLA:HLA00689 DRB1*04:04:01	HLA:HLA03486 DRB1*07:01:01:02	24	19
HLA:HLA00692 DRB1*04:06:01	HLA:HLA03486 DRB1*07:01:01:02	24	18
HLA:HLA00688 DRB1*04:03:01	HLA:HLA02017 DRB1*07:09	24	17
HLA:HLA00689 DRB1*04:04:01	HLA:HLA02017 DRB1*07:09	24	18
HLA:HLA00692 DRB1*04:06:01	HLA:HLA02017 DRB1*07:09	24	17
HLA:HLA00724 DRB1*08:02:01	HLA:HLA00750 DRB1*10:01:01	24	15
HLA:HLA00739 DRB1*08:13	HLA:HLA00750 DRB1*10:01:01	24	14
HLA:HLA00750 DRB1*10:01:01	HLA:HLA00751 DRB1*11:01:01	24	13
HLA:HLA00750 DRB1*10:01:01	HLA:HLA00769 DRB1*11:15	24	15
HLA:HLA00750 DRB1*10:01:01	HLA:HLA00797 DRB1*13:01:01	24	16
HLA:HLA00696 DRB1*04:10	HLA:HLA00865 DRB1*15:01:01:01	24	14
HLA:HLA00697 DRB1*04:11	HLA:HLA00865 DRB1*15:01:01:01	24	15
HLA:HLA00797 DRB1*13:01:01	HLA:HLA00865 DRB1*15:01:01:01	24	12
HLA:HLA00696 DRB1*04:10	HLA:HLA03453 DRB1*15:01:01:02	24	14
HLA:HLA00697 DRB1*04:11	HLA:HLA03453 DRB1*15:01:01:02	24	15
HLA:HLA00797 DRB1*13:01:01	HLA:HLA03453 DRB1*15:01:01:02	24	12
HLA:HLA00690 DRB1*04:05:01	HLA:HLA00867 DRB1*15:02:01	24	14
HLA:HLA00695 DRB1*04:09	HLA:HLA00867 DRB1*15:02:01	24	14
HLA:HLA00798 DRB1*13:02:01	HLA:HLA00867 DRB1*15:02:01	24	12
HLA:HLA00802 DRB1*13:05:01	HLA:HLA00867 DRB1*15:02:01	24	14
HLA:HLA00810 DRB1*13:12:01	HLA:HLA00867 DRB1*15:02:01	24	14
HLA:HLA01724 DRB1*13:56	HLA:HLA00867 DRB1*15:02:01	24	14
HLA:HLA00696 DRB1*04:10	HLA:HLA00870 DRB1*15:03:01:01	24	15
HLA:HLA00697 DRB1*04:11	HLA:HLA00870 DRB1*15:03:01:01	24	16
HLA:HLA00797 DRB1*13:01:01	HLA:HLA00870 DRB1*15:03:01:01	24	13
HLA:HLA00696 DRB1*04:10	HLA:HLA03454 DRB1*15:03:01:02	24	15
HLA:HLA00697 DRB1*04:11	HLA:HLA03454 DRB1*15:03:01:02	24	16
HLA:HLA00797 DRB1*13:01:01	HLA:HLA03454 DRB1*15:03:01:02	24	13
HLA:HLA00696 DRB1*04:10	HLA:HLA00871 DRB1*15:04	24	14
HLA:HLA00697 DRB1*04:11	HLA:HLA00871 DRB1*15:04	24	15
HLA:HLA00797 DRB1*13:01:01	HLA:HLA00871 DRB1*15:04	24	13
HLA:HLA02157 DRB1*11:11:02	HLA:HLA00876 DRB1*16:01:01	24	10
HLA:HLA00797 DRB1*13:01:01	HLA:HLA00876 DRB1*16:01:01	24	14
HLA:HLA02157 DRB1*11:11:02	HLA:HLA00878 DRB1*16:02:01	24	11
HLA:HLA00797 DRB1*13:01:01	HLA:HLA00878 DRB1*16:02:01	24	14
HLA:HLA00690 DRB1*04:05:01	HLA:HLA02257 DRB1*08:01:03	24	9
HLA:HLA00695 DRB1*04:09	HLA:HLA02257 DRB1*08:01:03	24	10
HLA:HLA00685 DRB1*04:01:01	HLA:HLA00724 DRB1*08:02:01	24	10
HLA:HLA00687 DRB1*04:02	HLA:HLA00724 DRB1*08:02:01	24	10
HLA:HLA00693 DRB1*04:07:01	HLA:HLA00724 DRB1*08:02:01	24	9
HLA:HLA00694 DRB1*04:08:01	HLA:HLA00724 DRB1*08:02:01	24	9
HLA:HLA00690 DRB1*04:05:01	HLA:HLA00727 DRB1*08:03:02	24	9
HLA:HLA00695 DRB1*04:09	HLA:HLA00727 DRB1*08:03:02	24	10
HLA:HLA00685 DRB1*04:01:01	HLA:HLA00739 DRB1*08:13	24	9
HLA:HLA00687 DRB1*04:02	HLA:HLA00739 DRB1*08:13	24	10
HLA:HLA00693 DRB1*04:07:01	HLA:HLA00739 DRB1*08:13	24	8
HLA:HLA00694 DRB1*04:08:01	HLA:HLA00739 DRB1*08:13	24	8
HLA:HLA00690 DRB1*04:05:01	HLA:HLA00744 DRB1*08:18	24	7
HLA:HLA00695 DRB1*04:09	HLA:HLA00744 DRB1*08:18	24	8
HLA:HLA00665 DRB1*01:02:01	HLA:HLA00750 DRB1*10:01:01	24	10
HLA:HLA00685 DRB1*04:01:01	HLA:HLA00750 DRB1*10:01:01	24	9
HLA:HLA00693 DRB1*04:07:01	HLA:HLA00750 DRB1*10:01:01	24	9
HLA:HLA00694 DRB1*04:08:01	HLA:HLA00750 DRB1*10:01:01	24	8
HLA:HLA00685 DRB1*04:01:01	HLA:HLA00751 DRB1*11:01:01	24	9
HLA:HLA00687 DRB1*04:02	HLA:HLA00751 DRB1*11:01:01	24	9
HLA:HLA00693 DRB1*04:07:01	HLA:HLA00751 DRB1*11:01:01	24	9
HLA:HLA00694 DRB1*04:08:01	HLA:HLA00751 DRB1*11:01:01	24	8
HLA:HLA00688 DRB1*04:03:01	HLA:HLA00755 DRB1*11:03	24	11
HLA:HLA00689 DRB1*04:04:01	HLA:HLA00755 DRB1*11:03	24	10
HLA:HLA00692 DRB1*04:06:01	HLA:HLA00755 DRB1*11:03	24	12
HLA:HLA00688 DRB1*04:03:01	HLA:HLA00756 DRB1*11:04:01	24	9
HLA:HLA00689 DRB1*04:04:01	HLA:HLA00756 DRB1*11:04:01	24	8
HLA:HLA00692 DRB1*04:06:01	HLA:HLA00756 DRB1*11:04:01	24	10
HLA:HLA00688 DRB1*04:03:01	HLA:HLA02157 DRB1*11:11:02	24	12
HLA:HLA00689 DRB1*04:04:01	HLA:HLA02157 DRB1*11:11:02	24	11
HLA:HLA00690 DRB1*04:05:01	HLA:HLA02157 DRB1*11:11:02	24	12
HLA:HLA00692 DRB1*04:06:01	HLA:HLA02157 DRB1*11:11:02	24	13
HLA:HLA00695 DRB1*04:09	HLA:HLA02157 DRB1*11:11:02	24	11
HLA:HLA00685 DRB1*04:01:01	HLA:HLA00769 DRB1*11:15	24	11
HLA:HLA00687 DRB1*04:02	HLA:HLA00769 DRB1*11:15	24	11
HLA:HLA00693 DRB1*04:07:01	HLA:HLA00769 DRB1*11:15	24	11
HLA:HLA00694 DRB1*04:08:01	HLA:HLA00769 DRB1*11:15	24	10
HLA:HLA00685 DRB1*04:01:01	HLA:HLA00797 DRB1*13:01:01	24	12
HLA:HLA02172 DRB1*04:06:02	HLA:HLA00797 DRB1*13:01:01	24	14
HLA:HLA00693 DRB1*04:07:01	HLA:HLA00797 DRB1*13:01:01	24	14

Allele 1	Allele 2	*m*	K_B
HLA:HLA00694 DRB1*04:08:01	HLA:HLA00797 DRB1*13:01:01	24	13
HLA:HLA00696 DRB1*04:10	HLA:HLA00797 DRB1*13:01:01	24	14
HLA:HLA00697 DRB1*04:11	HLA:HLA00797 DRB1*13:01:01	24	15
HLA:HLA00688 DRB1*04:03:01	HLA:HLA00798 DRB1*13:02:01	24	14
HLA:HLA00689 DRB1*04:04:01	HLA:HLA00798 DRB1*13:02:01	24	13
HLA:HLA00690 DRB1*04:05:01	HLA:HLA00798 DRB1*13:02:01	24	14
HLA:HLA00692 DRB1*04:06:01	HLA:HLA00798 DRB1*13:02:01	24	15
HLA:HLA00695 DRB1*04:09	HLA:HLA00798 DRB1*13:02:01	24	13
HLA:HLA00688 DRB1*04:03:01	HLA:HLA00802 DRB1*13:05:01	24	12
HLA:HLA00689 DRB1*04:04:01	HLA:HLA00802 DRB1*13:05:01	24	11
HLA:HLA00690 DRB1*04:05:01	HLA:HLA00802 DRB1*13:05:01	24	12
HLA:HLA00692 DRB1*04:06:01	HLA:HLA00802 DRB1*13:05:01	24	13
HLA:HLA00695 DRB1*04:09	HLA:HLA00802 DRB1*13:05:01	24	13
HLA:HLA00688 DRB1*04:03:01	HLA:HLA00810 DRB1*13:12:01	24	11
HLA:HLA00689 DRB1*04:04:01	HLA:HLA00810 DRB1*13:12:01	24	10
HLA:HLA00692 DRB1*04:06:01	HLA:HLA00810 DRB1*13:12:01	24	12
HLA:HLA00687 DRB1*04:02	HLA:HLA00825 DRB1*13:27	24	7
HLA:HLA00688 DRB1*04:03:01	HLA:HLA01724 DRB1*13:56	24	11
HLA:HLA00689 DRB1*04:04:01	HLA:HLA01724 DRB1*13:56	24	10
HLA:HLA00690 DRB1*04:05:01	HLA:HLA01724 DRB1*13:56	24	11
HLA:HLA00692 DRB1*04:06:01	HLA:HLA01724 DRB1*13:56	24	12
HLA:HLA00695 DRB1*04:09	HLA:HLA01724 DRB1*13:56	24	12
HLA:HLA00750 DRB1*10:01:01	HLA:HLA00839 DRB1*14:07:01	24	11
HLA:HLA00687 DRB1*04:02	HLA:HLA00719 DRB1*07:01:01:01	25	18
HLA:HLA02172 DRB1*04:06:02	HLA:HLA00719 DRB1*07:01:01:01	25	18
HLA:HLA00687 DRB1*04:02	HLA:HLA03486 DRB1*07:01:01:02	25	18
HLA:HLA02172 DRB1*04:06:02	HLA:HLA03486 DRB1*07:01:01:02	25	18
HLA:HLA00687 DRB1*04:02	HLA:HLA02017 DRB1*07:09	25	17
HLA:HLA02172 DRB1*04:06:02	HLA:HLA02017 DRB1*07:09	25	17
HLA:HLA02257 DRB1*08:01:03	HLA:HLA00750 DRB1*10:01:01	25	17
HLA:HLA00727 DRB1*08:03:02	HLA:HLA00750 DRB1*10:01:01	25	17
HLA:HLA00735 DRB1*08:09	HLA:HLA00750 DRB1*10:01:01	25	15
HLA:HLA00744 DRB1*08:18	HLA:HLA00750 DRB1*10:01:01	25	15
HLA:HLA00750 DRB1*10:01:01	HLA:HLA00752 DRB1*11:01:02	25	13
HLA:HLA00750 DRB1*10:01:01	HLA:HLA00755 DRB1*11:03	25	16
HLA:HLA00750 DRB1*10:01:01	HLA:HLA00756 DRB1*11:04:01	25	14
HLA:HLA00750 DRB1*10:01:01	HLA:HLA02157 DRB1*11:11:02	25	15
HLA:HLA00688 DRB1*04:03:01	HLA:HLA00865 DRB1*15:01:01:01	25	13
HLA:HLA00689 DRB1*04:04:01	HLA:HLA00865 DRB1*15:01:01:01	25	12
HLA:HLA00690 DRB1*04:05:01	HLA:HLA00865 DRB1*15:01:01:01	25	15
HLA:HLA00692 DRB1*04:06:01	HLA:HLA00865 DRB1*15:01:01:01	25	12
HLA:HLA00695 DRB1*04:09	HLA:HLA00865 DRB1*15:01:01:01	25	15
HLA:HLA00798 DRB1*13:02:01	HLA:HLA00865 DRB1*15:01:01:01	25	13
HLA:HLA00802 DRB1*13:05:01	HLA:HLA00865 DRB1*15:01:01:01	25	15
HLA:HLA00810 DRB1*13:12:01	HLA:HLA00865 DRB1*15:01:01:01	25	15
HLA:HLA01724 DRB1*13:56	HLA:HLA00865 DRB1*15:01:01:01	25	15
HLA:HLA00837 DRB1*14:05:01	HLA:HLA00865 DRB1*15:01:01:01	25	14
HLA:HLA02371 DRB1*14:54	HLA:HLA00865 DRB1*15:01:01:01	25	16
HLA:HLA00688 DRB1*04:03:01	HLA:HLA03453 DRB1*15:01:01:02	25	13
HLA:HLA00689 DRB1*04:04:01	HLA:HLA03453 DRB1*15:01:01:02	25	12
HLA:HLA00690 DRB1*04:05:01	HLA:HLA03453 DRB1*15:01:01:02	25	15
HLA:HLA00692 DRB1*04:06:01	HLA:HLA03453 DRB1*15:01:01:02	25	12
HLA:HLA00695 DRB1*04:09	HLA:HLA03453 DRB1*15:01:01:02	25	15
HLA:HLA00798 DRB1*13:02:01	HLA:HLA03453 DRB1*15:01:01:02	25	13
HLA:HLA00802 DRB1*13:05:01	HLA:HLA03453 DRB1*15:01:01:02	25	15
HLA:HLA00810 DRB1*13:12:01	HLA:HLA03453 DRB1*15:01:01:02	25	15
HLA:HLA01724 DRB1*13:56	HLA:HLA03453 DRB1*15:01:01:02	25	15
HLA:HLA00837 DRB1*14:05:01	HLA:HLA03453 DRB1*15:01:01:02	25	14
HLA:HLA02371 DRB1*14:54	HLA:HLA03453 DRB1*15:01:01:02	25	16
HLA:HLA00685 DRB1*04:01:01	HLA:HLA00867 DRB1*15:02:01	25	12
HLA:HLA00693 DRB1*04:07:01	HLA:HLA00867 DRB1*15:02:01	25	13
HLA:HLA00694 DRB1*04:08:01	HLA:HLA00867 DRB1*15:02:01	25	12
HLA:HLA00696 DRB1*04:10	HLA:HLA00867 DRB1*15:02:01	25	15
HLA:HLA00697 DRB1*04:11	HLA:HLA00867 DRB1*15:02:01	25	16
HLA:HLA00719 DRB1*07:01:01:01	HLA:HLA00867 DRB1*15:02:01	25	21
HLA:HLA03486 DRB1*07:01:01:02	HLA:HLA00867 DRB1*15:02:01	25	21
HLA:HLA02017 DRB1*07:09	HLA:HLA00867 DRB1*15:02:01	25	20
HLA:HLA02157 DRB1*11:11:02	HLA:HLA00867 DRB1*15:02:01	25	11
HLA:HLA00797 DRB1*13:01:01	HLA:HLA00867 DRB1*15:02:01	25	13
HLA:HLA00839 DRB1*14:07:01	HLA:HLA00867 DRB1*15:02:01	25	16
HLA:HLA00688 DRB1*04:03:01	HLA:HLA00870 DRB1*15:03:01:01	25	14
HLA:HLA00689 DRB1*04:04:01	HLA:HLA00870 DRB1*15:03:01:01	25	13
HLA:HLA00690 DRB1*04:05:01	HLA:HLA00870 DRB1*15:03:01:01	25	16
HLA:HLA00692 DRB1*04:06:01	HLA:HLA00870 DRB1*15:03:01:01	25	13
HLA:HLA00695 DRB1*04:09	HLA:HLA00870 DRB1*15:03:01:01	25	16
HLA:HLA00798 DRB1*13:02:01	HLA:HLA00870 DRB1*15:03:01:01	25	14
HLA:HLA00802 DRB1*13:05:01	HLA:HLA00870 DRB1*15:03:01:01	25	16
HLA:HLA00810 DRB1*13:12:01	HLA:HLA00870 DRB1*15:03:01:01	25	16
HLA:HLA01724 DRB1*13:56	HLA:HLA00870 DRB1*15:03:01:01	25	16
HLA:HLA00837 DRB1*14:05:01	HLA:HLA00870 DRB1*15:03:01:01	25	15
HLA:HLA02371 DRB1*14:54	HLA:HLA00870 DRB1*15:03:01:01	25	17
HLA:HLA00688 DRB1*04:03:01	HLA:HLA03454 DRB1*15:03:01:02	25	14
HLA:HLA00689 DRB1*04:04:01	HLA:HLA03454 DRB1*15:03:01:02	25	13
HLA:HLA00690 DRB1*04:05:01	HLA:HLA03454 DRB1*15:03:01:02	25	16
HLA:HLA00692 DRB1*04:06:01	HLA:HLA03454 DRB1*15:03:01:02	25	13
HLA:HLA00695 DRB1*04:09	HLA:HLA03454 DRB1*15:03:01:02	25	16
HLA:HLA00798 DRB1*13:02:01	HLA:HLA03454 DRB1*15:03:01:02	25	14
HLA:HLA00802 DRB1*13:05:01	HLA:HLA03454 DRB1*15:03:01:02	25	16
HLA:HLA00810 DRB1*13:12:01	HLA:HLA03454 DRB1*15:03:01:02	25	16
HLA:HLA01724 DRB1*13:56	HLA:HLA03454 DRB1*15:03:01:02	25	16
HLA:HLA00837 DRB1*14:05:01	HLA:HLA03454 DRB1*15:03:01:02	25	15
HLA:HLA02371 DRB1*14:54	HLA:HLA03454 DRB1*15:03:01:02	25	17
HLA:HLA00688 DRB1*04:03:01	HLA:HLA00871 DRB1*15:04	25	13
HLA:HLA00689 DRB1*04:04:01	HLA:HLA00871 DRB1*15:04	25	12
HLA:HLA00690 DRB1*04:05:01	HLA:HLA00871 DRB1*15:04	25	15
HLA:HLA00692 DRB1*04:06:01	HLA:HLA00871 DRB1*15:04	25	12
HLA:HLA00695 DRB1*04:09	HLA:HLA00871 DRB1*15:04	25	15
HLA:HLA00798 DRB1*13:02:01	HLA:HLA00871 DRB1*15:04	25	14
HLA:HLA00802 DRB1*13:05:01	HLA:HLA00871 DRB1*15:04	25	14
HLA:HLA00810 DRB1*13:12:01	HLA:HLA00871 DRB1*15:04	25	16
HLA:HLA01724 DRB1*13:56	HLA:HLA00871 DRB1*15:04	25	15
HLA:HLA00837 DRB1*14:05:01	HLA:HLA00871 DRB1*15:04	25	14
HLA:HLA02371 DRB1*14:54	HLA:HLA00871 DRB1*15:04	25	16
HLA:HLA00690 DRB1*04:05:01	HLA:HLA00876 DRB1*16:01:01	25	13
HLA:HLA00695 DRB1*04:09	HLA:HLA00876 DRB1*16:01:01	25	14
HLA:HLA02257 DRB1*08:01:03	HLA:HLA00876 DRB1*16:01:01	25	10
HLA:HLA00727 DRB1*08:03:02	HLA:HLA00876 DRB1*16:01:01	25	11
HLA:HLA00735 DRB1*08:09	HLA:HLA00876 DRB1*16:01:01	25	9
HLA:HLA00744 DRB1*08:18	HLA:HLA00876 DRB1*16:01:01	25	9
HLA:HLA00751 DRB1*11:01:01	HLA:HLA00876 DRB1*16:01:01	25	8
HLA:HLA00769 DRB1*11:15	HLA:HLA00876 DRB1*16:01:01	25	10
HLA:HLA00690 DRB1*04:05:01	HLA:HLA00878 DRB1*16:02:01	25	12
HLA:HLA00695 DRB1*04:09	HLA:HLA00878 DRB1*16:02:01	25	13
HLA:HLA02257 DRB1*08:01:03	HLA:HLA00878 DRB1*16:02:01	25	11
HLA:HLA00727 DRB1*08:03:02	HLA:HLA00878 DRB1*16:02:01	25	11
HLA:HLA00735 DRB1*08:09	HLA:HLA00878 DRB1*16:02:01	25	10
HLA:HLA00744 DRB1*08:18	HLA:HLA00878 DRB1*16:02:01	25	9
HLA:HLA00751 DRB1*11:01:01	HLA:HLA00878 DRB1*16:02:01	25	9
HLA:HLA00769 DRB1*11:15	HLA:HLA00878 DRB1*16:02:01	25	11
HLA:HLA00685 DRB1*04:01:01	HLA:HLA02257 DRB1*08:01:03	25	12
HLA:HLA00687 DRB1*04:02	HLA:HLA02257 DRB1*08:01:03	25	12
HLA:HLA00693 DRB1*04:07:01	HLA:HLA02257 DRB1*08:01:03	25	11
HLA:HLA00694 DRB1*04:08:01	HLA:HLA02257 DRB1*08:01:03	25	11
HLA:HLA00696 DRB1*04:10	HLA:HLA02257 DRB1*08:01:03	25	10
HLA:HLA00697 DRB1*04:11	HLA:HLA02257 DRB1*08:01:03	25	10
HLA:HLA00688 DRB1*04:03:01	HLA:HLA00724 DRB1*08:02:01	25	10
HLA:HLA00689 DRB1*04:04:01	HLA:HLA00724 DRB1*08:02:01	25	10
HLA:HLA00690 DRB1*04:05:01	HLA:HLA00724 DRB1*08:02:01	25	11
HLA:HLA00692 DRB1*04:06:01	HLA:HLA00724 DRB1*08:02:01	25	11
HLA:HLA00695 DRB1*04:09	HLA:HLA00724 DRB1*08:02:01	25	12
HLA:HLA00685 DRB1*04:01:01	HLA:HLA00727 DRB1*08:03:02	25	12
HLA:HLA00687 DRB1*04:02	HLA:HLA00727 DRB1*08:03:02	25	11
HLA:HLA00693 DRB1*04:07:01	HLA:HLA00727 DRB1*08:03:02	25	11
HLA:HLA00694 DRB1*04:08:01	HLA:HLA00727 DRB1*08:03:02	25	11
HLA:HLA00696 DRB1*04:10	HLA:HLA00727 DRB1*08:03:02	25	10
HLA:HLA00697 DRB1*04:11	HLA:HLA00727 DRB1*08:03:02	25	10
HLA:HLA00685 DRB1*04:01:01	HLA:HLA00735 DRB1*08:09	25	12
HLA:HLA00687 DRB1*04:02	HLA:HLA00735 DRB1*08:09	25	12
HLA:HLA00693 DRB1*04:07:01	HLA:HLA00735 DRB1*08:09	25	11
HLA:HLA00694 DRB1*04:08:01	HLA:HLA00735 DRB1*08:09	25	11
HLA:HLA00688 DRB1*04:03:01	HLA:HLA00739 DRB1*08:13	25	9
HLA:HLA00689 DRB1*04:04:01	HLA:HLA00739 DRB1*08:13	25	9
HLA:HLA00690 DRB1*04:05:01	HLA:HLA00739 DRB1*08:13	25	10
HLA:HLA00692 DRB1*04:06:01	HLA:HLA00739 DRB1*08:13	25	10
HLA:HLA00695 DRB1*04:09	HLA:HLA00739 DRB1*08:13	25	11
HLA:HLA00685 DRB1*04:01:01	HLA:HLA00744 DRB1*08:18	25	10
HLA:HLA00687 DRB1*04:02	HLA:HLA00744 DRB1*08:18	25	9
HLA:HLA00693 DRB1*04:07:01	HLA:HLA00744 DRB1*08:18	25	10
HLA:HLA00694 DRB1*04:08:01	HLA:HLA00744 DRB1*08:18	25	9
HLA:HLA00696 DRB1*04:10	HLA:HLA00744 DRB1*08:18	25	8
HLA:HLA00697 DRB1*04:11	HLA:HLA00744 DRB1*08:18	25	9
HLA:HLA00688 DRB1*04:03:01	HLA:HLA00750 DRB1*10:01:01	25	10
HLA:HLA00689 DRB1*04:04:01	HLA:HLA00750 DRB1*10:01:01	25	9
HLA:HLA00690 DRB1*04:05:01	HLA:HLA00750 DRB1*10:01:01	25	10
HLA:HLA00692 DRB1*04:06:01	HLA:HLA00750 DRB1*10:01:01	25	11
HLA:HLA00695 DRB1*04:09	HLA:HLA00750 DRB1*10:01:01	25	11
HLA:HLA00688 DRB1*04:03:01	HLA:HLA00751 DRB1*11:01:01	25	10
HLA:HLA00689 DRB1*04:04:01	HLA:HLA00751 DRB1*11:01:01	25	9
HLA:HLA00690 DRB1*04:05:01	HLA:HLA00751 DRB1*11:01:01	25	10
HLA:HLA00692 DRB1*04:06:01	HLA:HLA00751 DRB1*11:01:01	25	11
HLA:HLA00695 DRB1*04:09	HLA:HLA00751 DRB1*11:01:01	25	11
HLA:HLA00685 DRB1*04:01:01	HLA:HLA00752 DRB1*11:01:02	25	9
HLA:HLA00687 DRB1*04:02	HLA:HLA00752 DRB1*11:01:02	25	9
HLA:HLA00693 DRB1*04:07:01	HLA:HLA00752 DRB1*11:01:02	25	9
HLA:HLA00694 DRB1*04:08:01	HLA:HLA00752 DRB1*11:01:02	25	8
HLA:HLA00685 DRB1*04:01:01	HLA:HLA00755 DRB1*11:03	25	10
HLA:HLA02172 DRB1*04:06:02	HLA:HLA00755 DRB1*11:03	25	12
HLA:HLA00693 DRB1*04:07:01	HLA:HLA00755 DRB1*11:03	25	12
HLA:HLA00694 DRB1*04:08:01	HLA:HLA00755 DRB1*11:03	25	11
HLA:HLA00696 DRB1*04:10	HLA:HLA00755 DRB1*11:03	25	12
HLA:HLA00697 DRB1*04:11	HLA:HLA00755 DRB1*11:03	25	13
HLA:HLA00685 DRB1*04:01:01	HLA:HLA00756 DRB1*11:04:01	25	10
HLA:HLA02172 DRB1*04:06:02	HLA:HLA00756 DRB1*11:04:01	25	10
HLA:HLA00693 DRB1*04:07:01	HLA:HLA00756 DRB1*11:04:01	25	10
HLA:HLA00694 DRB1*04:08:01	HLA:HLA00756 DRB1*11:04:01	25	9
HLA:HLA00696 DRB1*04:10	HLA:HLA00756 DRB1*11:04:01	25	10
HLA:HLA00697 DRB1*04:11	HLA:HLA00756 DRB1*11:04:01	25	11
HLA:HLA02172 DRB1*04:06:02	HLA:HLA02157 DRB1*11:11:02	25	13
HLA:HLA00696 DRB1*04:10	HLA:HLA02157 DRB1*11:11:02	25	13
HLA:HLA00697 DRB1*04:11	HLA:HLA02157 DRB1*11:11:02	25	14
HLA:HLA00688 DRB1*04:03:01	HLA:HLA00769 DRB1*11:15	25	12
HLA:HLA00689 DRB1*04:04:01	HLA:HLA00769 DRB1*11:15	25	11
HLA:HLA00690 DRB1*04:05:01	HLA:HLA00769 DRB1*11:15	25	12
HLA:HLA00692 DRB1*04:06:01	HLA:HLA00769 DRB1*11:15	25	13
HLA:HLA00695 DRB1*04:09	HLA:HLA00769 DRB1*11:15	25	13
HLA:HLA00690 DRB1*04:05:01	HLA:HLA00797 DRB1*13:01:01	25	15
HLA:HLA00695 DRB1*04:09	HLA:HLA00797 DRB1*13:01:01	25	14
HLA:HLA02172 DRB1*04:06:02	HLA:HLA00798 DRB1*13:02:01	25	15
HLA:HLA00696 DRB1*04:10	HLA:HLA00798 DRB1*13:02:01	25	15
HLA:HLA00697 DRB1*04:11	HLA:HLA00798 DRB1*13:02:01	25	16
HLA:HLA02172 DRB1*04:06:02	HLA:HLA00802 DRB1*13:05:01	25	13
HLA:HLA00696 DRB1*04:10	HLA:HLA00802 DRB1*13:05:01	25	13
HLA:HLA00697 DRB1*04:11	HLA:HLA00802 DRB1*13:05:01	25	14
HLA:HLA02172 DRB1*04:06:02	HLA:HLA00810 DRB1*13:12:01	25	12
HLA:HLA00688 DRB1*04:03:01	HLA:HLA00825 DRB1*13:27	25	13
HLA:HLA00689 DRB1*04:04:01	HLA:HLA00825 DRB1*13:27	25	12

Allele 1	Allele 2	m	K_B
HLA:HLA00692 DRB1*04:06:01	HLA:HLA00825 DRB1*13:27	25	14
HLA:HLA02172 DRB1*04:06:02	HLA:HLA01724 DRB1*13:56	25	12
HLA:HLA00696 DRB1*04:10	HLA:HLA01724 DRB1*13:56	25	12
HLA:HLA00697 DRB1*04:11	HLA:HLA01724 DRB1*13:56	25	13
HLA:HLA00685 DRB1*04:01:01	HLA:HLA00839 DRB1*14:07:01	25	11
HLA:HLA00693 DRB1*04:07:01	HLA:HLA00839 DRB1*14:07:01	25	9
HLA:HLA00694 DRB1*04:08:01	HLA:HLA00839 DRB1*14:07:01	25	10
HLA:HLA00665 DRB1*01:02:01	HLA:HLA01693 DRB1*14:46	25	10
HLA:HLA00688 DRB1*04:03:01	HLA:HLA02371 DRB1*14:54	25	9
HLA:HLA00689 DRB1*04:04:01	HLA:HLA02371 DRB1*14:54	25	10
HLA:HLA00692 DRB1*04:06:01	HLA:HLA02371 DRB1*14:54	25	9
HLA:HLA00750 DRB1*10:01:01	HLA:HLA02371 DRB1*14:54	25	12
HLA:HLA00749 DRB1*09:01:02	HLA:HLA00750 DRB1*10:01:01	26	15
HLA:HLA00750 DRB1*10:01:01	HLA:HLA01407 DRB1*12:01:02	26	18
HLA:HLA00750 DRB1*10:01:01	HLA:HLA00825 DRB1*13:27	26	16
HLA:HLA00749 DRB1*09:01:02	HLA:HLA01693 DRB1*14:46	26	17
HLA:HLA00685 DRB1*04:01:01	HLA:HLA00865 DRB1*15:01:01:01	26	13
HLA:HLA00687 DRB1*04:02	HLA:HLA00865 DRB1*15:01:01:01	26	12
HLA:HLA02172 DRB1*04:06:02	HLA:HLA00865 DRB1*15:01:01:01	26	12
HLA:HLA00693 DRB1*04:07:01	HLA:HLA00865 DRB1*15:01:01:01	26	14
HLA:HLA00694 DRB1*04:08:01	HLA:HLA00865 DRB1*15:01:01:01	26	13
HLA:HLA00719 DRB1*07:01:01:01	HLA:HLA00865 DRB1*15:01:01:01	26	22
HLA:HLA03486 DRB1*07:01:01:02	HLA:HLA00865 DRB1*15:01:01:01	26	22
HLA:HLA02017 DRB1*07:09	HLA:HLA00865 DRB1*15:01:01:01	26	21
HLA:HLA00755 DRB1*11:03	HLA:HLA00865 DRB1*15:01:01:01	26	11
HLA:HLA00756 DRB1*11:04:01	HLA:HLA00865 DRB1*15:01:01:01	26	12
HLA:HLA02157 DRB1*11:11:02	HLA:HLA00865 DRB1*15:01:01:01	26	12
HLA:HLA01407 DRB1*12:01:02	HLA:HLA00865 DRB1*15:01:01:01	26	18
HLA:HLA00825 DRB1*13:27	HLA:HLA00865 DRB1*15:01:01:01	26	12
HLA:HLA00833 DRB1*14:01:01	HLA:HLA00865 DRB1*15:01:01:01	26	16
HLA:HLA00836 DRB1*14:04	HLA:HLA00865 DRB1*15:01:01:01	26	15
HLA:HLA00839 DRB1*14:07:01	HLA:HLA00865 DRB1*15:01:01:01	26	17
HLA:HLA00685 DRB1*04:01:01	HLA:HLA03453 DRB1*15:01:01:02	26	13
HLA:HLA00687 DRB1*04:02	HLA:HLA03453 DRB1*15:01:01:02	26	12
HLA:HLA02172 DRB1*04:06:02	HLA:HLA03453 DRB1*15:01:01:02	26	12
HLA:HLA00693 DRB1*04:07:01	HLA:HLA03453 DRB1*15:01:01:02	26	14
HLA:HLA00694 DRB1*04:08:01	HLA:HLA03453 DRB1*15:01:01:02	26	13
HLA:HLA00719 DRB1*07:01:01:01	HLA:HLA03453 DRB1*15:01:01:02	26	22
HLA:HLA03486 DRB1*07:01:01:02	HLA:HLA03453 DRB1*15:01:01:02	26	22
HLA:HLA02017 DRB1*07:09	HLA:HLA03453 DRB1*15:01:01:02	26	21
HLA:HLA00755 DRB1*11:03	HLA:HLA03453 DRB1*15:01:01:02	26	11
HLA:HLA00756 DRB1*11:04:01	HLA:HLA03453 DRB1*15:01:01:02	26	12
HLA:HLA02157 DRB1*11:11:02	HLA:HLA03453 DRB1*15:01:01:02	26	12
HLA:HLA01407 DRB1*12:01:02	HLA:HLA03453 DRB1*15:01:01:02	26	18
HLA:HLA00825 DRB1*13:27	HLA:HLA03453 DRB1*15:01:01:02	26	12
HLA:HLA00833 DRB1*14:01:01	HLA:HLA03453 DRB1*15:01:01:02	26	16
HLA:HLA00836 DRB1*14:04	HLA:HLA03453 DRB1*15:01:01:02	26	15
HLA:HLA00839 DRB1*14:07:01	HLA:HLA03453 DRB1*15:01:01:02	26	17
HLA:HLA00688 DRB1*04:03:01	HLA:HLA00867 DRB1*15:02:01	26	14
HLA:HLA00689 DRB1*04:04:01	HLA:HLA00867 DRB1*15:02:01	26	13
HLA:HLA00692 DRB1*04:06:01	HLA:HLA00867 DRB1*15:02:01	26	13
HLA:HLA02257 DRB1*08:01:03	HLA:HLA00867 DRB1*15:02:01	26	16
HLA:HLA00727 DRB1*08:03:02	HLA:HLA00867 DRB1*15:02:01	26	15
HLA:HLA00735 DRB1*08:09	HLA:HLA00867 DRB1*15:02:01	26	15
HLA:HLA00744 DRB1*08:18	HLA:HLA00867 DRB1*15:02:01	26	13
HLA:HLA00749 DRB1*09:01:02	HLA:HLA00867 DRB1*15:02:01	26	23
HLA:HLA00751 DRB1*11:01:01	HLA:HLA00867 DRB1*15:02:01	26	12
HLA:HLA00769 DRB1*11:15	HLA:HLA00867 DRB1*15:02:01	26	14
HLA:HLA00837 DRB1*14:05:01	HLA:HLA00867 DRB1*15:02:01	26	15
HLA:HLA02371 DRB1*14:54	HLA:HLA00867 DRB1*15:02:01	26	17
HLA:HLA00685 DRB1*04:01:01	HLA:HLA00870 DRB1*15:03:01:01	26	14
HLA:HLA00687 DRB1*04:02	HLA:HLA00870 DRB1*15:03:01:01	26	13
HLA:HLA02172 DRB1*04:06:02	HLA:HLA00870 DRB1*15:03:01:01	26	13
HLA:HLA00693 DRB1*04:07:01	HLA:HLA00870 DRB1*15:03:01:01	26	15
HLA:HLA00694 DRB1*04:08:01	HLA:HLA00870 DRB1*15:03:01:01	26	14
HLA:HLA00719 DRB1*07:01:01:01	HLA:HLA00870 DRB1*15:03:01:01	26	21
HLA:HLA03486 DRB1*07:01:01:02	HLA:HLA00870 DRB1*15:03:01:01	26	21
HLA:HLA02017 DRB1*07:09	HLA:HLA00870 DRB1*15:03:01:01	26	22
HLA:HLA00755 DRB1*11:03	HLA:HLA00870 DRB1*15:03:01:01	26	12
HLA:HLA00756 DRB1*11:04:01	HLA:HLA00870 DRB1*15:03:01:01	26	13
HLA:HLA02157 DRB1*11:11:02	HLA:HLA00870 DRB1*15:03:01:01	26	13
HLA:HLA01407 DRB1*12:01:02	HLA:HLA00870 DRB1*15:03:01:01	26	17
HLA:HLA00825 DRB1*13:27	HLA:HLA00870 DRB1*15:03:01:01	26	13
HLA:HLA00833 DRB1*14:01:01	HLA:HLA00870 DRB1*15:03:01:01	26	17
HLA:HLA00836 DRB1*14:04	HLA:HLA00870 DRB1*15:03:01:01	26	16
HLA:HLA00839 DRB1*14:07:01	HLA:HLA00870 DRB1*15:03:01:01	26	18
HLA:HLA00685 DRB1*04:01:01	HLA:HLA03454 DRB1*15:03:01:02	26	14
HLA:HLA00687 DRB1*04:02	HLA:HLA03454 DRB1*15:03:01:02	26	13
HLA:HLA02172 DRB1*04:06:02	HLA:HLA03454 DRB1*15:03:01:02	26	13
HLA:HLA00693 DRB1*04:07:01	HLA:HLA03454 DRB1*15:03:01:02	26	15
HLA:HLA00694 DRB1*04:08:01	HLA:HLA03454 DRB1*15:03:01:02	26	14
HLA:HLA00719 DRB1*07:01:01:01	HLA:HLA03454 DRB1*15:03:01:02	26	21
HLA:HLA03486 DRB1*07:01:01:02	HLA:HLA03454 DRB1*15:03:01:02	26	21
HLA:HLA02017 DRB1*07:09	HLA:HLA03454 DRB1*15:03:01:02	26	22
HLA:HLA00755 DRB1*11:03	HLA:HLA03454 DRB1*15:03:01:02	26	12
HLA:HLA00756 DRB1*11:04:01	HLA:HLA03454 DRB1*15:03:01:02	26	13
HLA:HLA02157 DRB1*11:11:02	HLA:HLA03454 DRB1*15:03:01:02	26	13
HLA:HLA01407 DRB1*12:01:02	HLA:HLA03454 DRB1*15:03:01:02	26	17
HLA:HLA00825 DRB1*13:27	HLA:HLA03454 DRB1*15:03:01:02	26	13
HLA:HLA00833 DRB1*14:01:01	HLA:HLA03454 DRB1*15:03:01:02	26	17
HLA:HLA00836 DRB1*14:04	HLA:HLA03454 DRB1*15:03:01:02	26	16
HLA:HLA00839 DRB1*14:07:01	HLA:HLA03454 DRB1*15:03:01:02	26	18
HLA:HLA00685 DRB1*04:01:01	HLA:HLA00871 DRB1*15:04	26	13
HLA:HLA00687 DRB1*04:02	HLA:HLA00871 DRB1*15:04	26	13
HLA:HLA02172 DRB1*04:06:02	HLA:HLA00871 DRB1*15:04	26	12
HLA:HLA00693 DRB1*04:07:01	HLA:HLA00871 DRB1*15:04	26	14
HLA:HLA00694 DRB1*04:08:01	HLA:HLA00871 DRB1*15:04	26	13
HLA:HLA00719 DRB1*07:01:01:01	HLA:HLA00871 DRB1*15:04	26	23
HLA:HLA03486 DRB1*07:01:01:02	HLA:HLA00871 DRB1*15:04	26	23
HLA:HLA02017 DRB1*07:09	HLA:HLA00871 DRB1*15:04	26	22
HLA:HLA00755 DRB1*11:03	HLA:HLA00871 DRB1*15:04	26	10
HLA:HLA00756 DRB1*11:04:01	HLA:HLA00871 DRB1*15:04	26	11
HLA:HLA02157 DRB1*11:11:02	HLA:HLA00871 DRB1*15:04	26	11
HLA:HLA01407 DRB1*12:01:02	HLA:HLA00871 DRB1*15:04	26	19
HLA:HLA00825 DRB1*13:27	HLA:HLA00871 DRB1*15:04	26	13
HLA:HLA00833 DRB1*14:01:01	HLA:HLA00871 DRB1*15:04	26	16
HLA:HLA00836 DRB1*14:04	HLA:HLA00871 DRB1*15:04	26	15
HLA:HLA00839 DRB1*14:07:01	HLA:HLA00871 DRB1*15:04	26	17
HLA:HLA00685 DRB1*04:01:01	HLA:HLA00876 DRB1*16:01:01	26	12
HLA:HLA00687 DRB1*04:02	HLA:HLA00876 DRB1*16:01:01	26	12
HLA:HLA00693 DRB1*04:07:01	HLA:HLA00876 DRB1*16:01:01	26	12
HLA:HLA00694 DRB1*04:08:01	HLA:HLA00876 DRB1*16:01:01	26	11
HLA:HLA00696 DRB1*04:10	HLA:HLA00876 DRB1*16:01:01	26	14
HLA:HLA00697 DRB1*04:11	HLA:HLA00876 DRB1*16:01:01	26	15
HLA:HLA00719 DRB1*07:01:01:01	HLA:HLA00876 DRB1*16:01:01	26	17
HLA:HLA03486 DRB1*07:01:01:02	HLA:HLA00876 DRB1*16:01:01	26	17
HLA:HLA02017 DRB1*07:09	HLA:HLA00876 DRB1*16:01:01	26	16
HLA:HLA00724 DRB1*08:02:01	HLA:HLA00876 DRB1*16:01:01	26	8
HLA:HLA00739 DRB1*08:13	HLA:HLA00876 DRB1*16:01:01	26	9
HLA:HLA00752 DRB1*11:01:02	HLA:HLA00876 DRB1*16:01:01	26	8
HLA:HLA00755 DRB1*11:03	HLA:HLA00876 DRB1*16:01:01	26	11
HLA:HLA00756 DRB1*11:04:01	HLA:HLA00876 DRB1*16:01:01	26	9
HLA:HLA01407 DRB1*12:01:02	HLA:HLA00876 DRB1*16:01:01	26	17
HLA:HLA00825 DRB1*13:27	HLA:HLA00876 DRB1*16:01:01	26	14
HLA:HLA00685 DRB1*04:01:01	HLA:HLA00878 DRB1*16:02:01	26	11
HLA:HLA00687 DRB1*04:02	HLA:HLA00878 DRB1*16:02:01	26	12
HLA:HLA00693 DRB1*04:07:01	HLA:HLA00878 DRB1*16:02:01	26	11
HLA:HLA00694 DRB1*04:08:01	HLA:HLA00878 DRB1*16:02:01	26	10
HLA:HLA00696 DRB1*04:10	HLA:HLA00878 DRB1*16:02:01	26	13
HLA:HLA00697 DRB1*04:11	HLA:HLA00878 DRB1*16:02:01	26	14
HLA:HLA00719 DRB1*07:01:01:01	HLA:HLA00878 DRB1*16:02:01	26	17
HLA:HLA03486 DRB1*07:01:01:02	HLA:HLA00878 DRB1*16:02:01	26	17
HLA:HLA02017 DRB1*07:09	HLA:HLA00878 DRB1*16:02:01	26	16
HLA:HLA00724 DRB1*08:02:01	HLA:HLA00878 DRB1*16:02:01	26	9
HLA:HLA00739 DRB1*08:13	HLA:HLA00878 DRB1*16:02:01	26	8
HLA:HLA00752 DRB1*11:01:02	HLA:HLA00878 DRB1*16:02:01	26	9
HLA:HLA00755 DRB1*11:03	HLA:HLA00878 DRB1*16:02:01	26	12
HLA:HLA00756 DRB1*11:04:01	HLA:HLA00878 DRB1*16:02:01	26	10
HLA:HLA01407 DRB1*12:01:02	HLA:HLA00878 DRB1*16:02:01	26	17
HLA:HLA00825 DRB1*13:27	HLA:HLA00878 DRB1*16:02:01	26	14
HLA:HLA00671 DRB1*03:01:01:01	HLA:HLA00688 DRB1*04:03:01	26	10
HLA:HLA03483 DRB1*03:01:01:02	HLA:HLA00688 DRB1*04:03:01	26	10
HLA:HLA00678 DRB1*03:06	HLA:HLA00688 DRB1*04:03:01	26	9
HLA:HLA00671 DRB1*03:01:01:01	HLA:HLA00689 DRB1*04:04:01	26	10
HLA:HLA03483 DRB1*03:01:01:02	HLA:HLA00689 DRB1*04:04:01	26	10
HLA:HLA00678 DRB1*03:06	HLA:HLA00689 DRB1*04:04:01	26	9
HLA:HLA00671 DRB1*03:01:01:01	HLA:HLA00692 DRB1*04:06:01	26	11
HLA:HLA03483 DRB1*03:01:01:02	HLA:HLA00692 DRB1*04:06:01	26	11
HLA:HLA00678 DRB1*03:06	HLA:HLA00692 DRB1*04:06:01	26	10
HLA:HLA00688 DRB1*04:03:01	HLA:HLA02257 DRB1*08:01:03	26	12
HLA:HLA00689 DRB1*04:04:01	HLA:HLA02257 DRB1*08:01:03	26	12
HLA:HLA00692 DRB1*04:06:01	HLA:HLA02257 DRB1*08:01:03	26	13
HLA:HLA02172 DRB1*04:06:02	HLA:HLA00724 DRB1*08:02:01	26	11
HLA:HLA00696 DRB1*04:10	HLA:HLA00724 DRB1*08:02:01	26	12
HLA:HLA00697 DRB1*04:11	HLA:HLA00724 DRB1*08:02:01	26	12
HLA:HLA00688 DRB1*04:03:01	HLA:HLA00727 DRB1*08:03:02	26	12
HLA:HLA00689 DRB1*04:04:01	HLA:HLA00727 DRB1*08:03:02	26	12
HLA:HLA00692 DRB1*04:06:01	HLA:HLA00727 DRB1*08:03:02	26	13
HLA:HLA00688 DRB1*04:03:01	HLA:HLA00735 DRB1*08:09	26	12
HLA:HLA00689 DRB1*04:04:01	HLA:HLA00735 DRB1*08:09	26	12
HLA:HLA00690 DRB1*04:05:01	HLA:HLA00735 DRB1*08:09	26	13
HLA:HLA00692 DRB1*04:06:01	HLA:HLA00735 DRB1*08:09	26	12
HLA:HLA00695 DRB1*04:09	HLA:HLA00735 DRB1*08:09	26	14
HLA:HLA02172 DRB1*04:06:02	HLA:HLA00739 DRB1*08:13	26	10
HLA:HLA00696 DRB1*04:10	HLA:HLA00739 DRB1*08:13	26	11
HLA:HLA00697 DRB1*04:11	HLA:HLA00739 DRB1*08:13	26	11
HLA:HLA00688 DRB1*04:03:01	HLA:HLA00744 DRB1*08:18	26	11
HLA:HLA00689 DRB1*04:04:01	HLA:HLA00744 DRB1*08:18	26	10
HLA:HLA00692 DRB1*04:06:01	HLA:HLA00744 DRB1*08:18	26	12
HLA:HLA00671 DRB1*03:01:01:01	HLA:HLA00750 DRB1*10:01:01	26	14
HLA:HLA03483 DRB1*03:01:01:02	HLA:HLA00750 DRB1*10:01:01	26	14
HLA:HLA00678 DRB1*03:06	HLA:HLA00750 DRB1*10:01:01	26	13
HLA:HLA02172 DRB1*04:06:02	HLA:HLA00750 DRB1*10:01:01	26	11
HLA:HLA00696 DRB1*04:10	HLA:HLA00750 DRB1*10:01:01	26	11
HLA:HLA00697 DRB1*04:11	HLA:HLA00750 DRB1*10:01:01	26	12
HLA:HLA02172 DRB1*04:06:02	HLA:HLA00751 DRB1*11:01:01	26	11
HLA:HLA00696 DRB1*04:10	HLA:HLA00751 DRB1*11:01:01	26	11
HLA:HLA00697 DRB1*04:11	HLA:HLA00751 DRB1*11:01:01	26	12
HLA:HLA00688 DRB1*04:03:01	HLA:HLA00752 DRB1*11:01:02	26	10
HLA:HLA00689 DRB1*04:04:01	HLA:HLA00752 DRB1*11:01:02	26	9
HLA:HLA00690 DRB1*04:05:01	HLA:HLA00752 DRB1*11:01:02	26	10
HLA:HLA00692 DRB1*04:06:01	HLA:HLA00752 DRB1*11:01:02	26	11
HLA:HLA00695 DRB1*04:09	HLA:HLA00752 DRB1*11:01:02	26	11
HLA:HLA00690 DRB1*04:05:01	HLA:HLA00755 DRB1*11:03	26	13
HLA:HLA00695 DRB1*04:09	HLA:HLA00755 DRB1*11:03	26	12
HLA:HLA00690 DRB1*04:05:01	HLA:HLA00756 DRB1*11:04:01	26	11
HLA:HLA00695 DRB1*04:09	HLA:HLA00756 DRB1*11:04:01	26	12
HLA:HLA02172 DRB1*04:06:02	HLA:HLA00769 DRB1*11:15	26	13
HLA:HLA00696 DRB1*04:10	HLA:HLA00769 DRB1*11:15	26	13
HLA:HLA00697 DRB1*04:11	HLA:HLA00769 DRB1*11:15	26	14
HLA:HLA00687 DRB1*04:02	HLA:HLA01407 DRB1*12:01:02	26	16
HLA:HLA00696 DRB1*04:10	HLA:HLA01407 DRB1*12:01:02	26	18
HLA:HLA00697 DRB1*04:11	HLA:HLA01407 DRB1*12:01:02	26	19
HLA:HLA00665 DRB1*01:02:01	HLA:HLA00797 DRB1*13:01:01	26	17
HLA:HLA00685 DRB1*04:01:01	HLA:HLA00825 DRB1*13:27	26	12
HLA:HLA02172 DRB1*04:06:02	HLA:HLA00825 DRB1*13:27	26	14

Allele 1	Allele 2	*m*	K_B
HLA:HLA00693 DRB1*04:07:01	HLA:HLA00825 DRB1*13:27	26	14
HLA:HLA00694 DRB1*04:08:01	HLA:HLA00825 DRB1*13:27	26	13
HLA:HLA00696 DRB1*04:10	HLA:HLA00825 DRB1*13:27	26	14
HLA:HLA00697 DRB1*04:11	HLA:HLA00825 DRB1*13:27	26	15
HLA:HLA00688 DRB1*04:03:01	HLA:HLA00833 DRB1*14:01:01	26	9
HLA:HLA00689 DRB1*04:04:01	HLA:HLA00833 DRB1*14:01:01	26	10
HLA:HLA00692 DRB1*04:06:01	HLA:HLA00833 DRB1*14:01:01	26	9
HLA:HLA00750 DRB1*10:01:01	HLA:HLA00833 DRB1*14:01:01	26	12
HLA:HLA00688 DRB1*04:03:01	HLA:HLA00836 DRB1*14:04	26	9
HLA:HLA00689 DRB1*04:04:01	HLA:HLA00836 DRB1*14:04	26	10
HLA:HLA00692 DRB1*04:06:01	HLA:HLA00836 DRB1*14:04	26	9
HLA:HLA00750 DRB1*10:01:01	HLA:HLA00836 DRB1*14:04	26	13
HLA:HLA00688 DRB1*04:03:01	HLA:HLA00837 DRB1*14:05:01	26	7
HLA:HLA00689 DRB1*04:04:01	HLA:HLA00837 DRB1*14:05:01	26	8
HLA:HLA00692 DRB1*04:06:01	HLA:HLA00837 DRB1*14:05:01	26	7
HLA:HLA00750 DRB1*10:01:01	HLA:HLA00837 DRB1*14:05:01	26	10
HLA:HLA00688 DRB1*04:03:01	HLA:HLA00839 DRB1*14:07:01	26	10
HLA:HLA00689 DRB1*04:04:01	HLA:HLA00839 DRB1*14:07:01	26	11
HLA:HLA00690 DRB1*04:05:01	HLA:HLA00839 DRB1*14:07:01	26	11
HLA:HLA00692 DRB1*04:06:01	HLA:HLA00839 DRB1*14:07:01	26	10
HLA:HLA00695 DRB1*04:09	HLA:HLA00839 DRB1*14:07:01	26	12
HLA:HLA00665 DRB1*01:02:01	HLA:HLA02371 DRB1*14:54	26	14
HLA:HLA00685 DRB1*04:01:01	HLA:HLA02371 DRB1*14:54	26	12
HLA:HLA02172 DRB1*04:06:02	HLA:HLA02371 DRB1*14:54	26	9
HLA:HLA00693 DRB1*04:07:01	HLA:HLA02371 DRB1*14:54	26	10
HLA:HLA00694 DRB1*04:08:01	HLA:HLA02371 DRB1*14:54	26	11
HLA:HLA00696 DRB1*04:10	HLA:HLA02371 DRB1*14:54	26	11
HLA:HLA00697 DRB1*04:11	HLA:HLA02371 DRB1*14:54	26	10
HLA:HLA00839 DRB1*14:07:01	HLA:HLA00876 DRB1*16:01:01	26	14
HLA:HLA00839 DRB1*14:07:01	HLA:HLA00878 DRB1*16:02:01	26	13
HLA:HLA00687 DRB1*04:02	HLA:HLA00750 DRB1*10:01:01	27	14
HLA:HLA00687 DRB1*04:02	HLA:HLA02371 DRB1*14:54	27	15
HLA:HLA00671 DRB1*03:01:01:01	HLA:HLA00865 DRB1*15:01:01:01	27	14
HLA:HLA03483 DRB1*03:01:01:02	HLA:HLA00865 DRB1*15:01:01:01	27	14
HLA:HLA00678 DRB1*03:06	HLA:HLA00865 DRB1*15:01:01:01	27	15
HLA:HLA02257 DRB1*08:01:03	HLA:HLA00865 DRB1*15:01:01:01	27	17
HLA:HLA00727 DRB1*08:03:02	HLA:HLA00865 DRB1*15:01:01:01	27	16
HLA:HLA00735 DRB1*08:09	HLA:HLA00865 DRB1*15:01:01:01	27	16
HLA:HLA00744 DRB1*08:18	HLA:HLA00865 DRB1*15:01:01:01	27	14
HLA:HLA00749 DRB1*09:01:02	HLA:HLA00865 DRB1*15:01:01:01	27	24
HLA:HLA00751 DRB1*11:01:01	HLA:HLA00865 DRB1*15:01:01:01	27	13
HLA:HLA00769 DRB1*11:15	HLA:HLA00865 DRB1*15:01:01:01	27	15
HLA:HLA00843 DRB1*14:11	HLA:HLA00865 DRB1*15:01:01:01	27	13
HLA:HLA00671 DRB1*03:01:01:01	HLA:HLA03453 DRB1*15:01:01:02	27	14
HLA:HLA03483 DRB1*03:01:01:02	HLA:HLA03453 DRB1*15:01:01:02	27	14
HLA:HLA00678 DRB1*03:06	HLA:HLA03453 DRB1*15:01:01:02	27	15
HLA:HLA02257 DRB1*08:01:03	HLA:HLA03453 DRB1*15:01:01:02	27	17
HLA:HLA00727 DRB1*08:03:02	HLA:HLA03453 DRB1*15:01:01:02	27	16
HLA:HLA00735 DRB1*08:09	HLA:HLA03453 DRB1*15:01:01:02	27	16
HLA:HLA00744 DRB1*08:18	HLA:HLA03453 DRB1*15:01:01:02	27	14
HLA:HLA00749 DRB1*09:01:02	HLA:HLA03453 DRB1*15:01:01:02	27	24
HLA:HLA00751 DRB1*11:01:01	HLA:HLA03453 DRB1*15:01:01:02	27	13
HLA:HLA00769 DRB1*11:15	HLA:HLA03453 DRB1*15:01:01:02	27	15
HLA:HLA00843 DRB1*14:11	HLA:HLA03453 DRB1*15:01:01:02	27	13
HLA:HLA00687 DRB1*04:02	HLA:HLA00867 DRB1*15:02:01	27	13
HLA:HLA02172 DRB1*04:06:02	HLA:HLA00867 DRB1*15:02:01	27	13
HLA:HLA00724 DRB1*08:02:01	HLA:HLA00867 DRB1*15:02:01	27	14
HLA:HLA00739 DRB1*08:13	HLA:HLA00867 DRB1*15:02:01	27	14
HLA:HLA00752 DRB1*11:01:02	HLA:HLA00867 DRB1*15:02:01	27	12
HLA:HLA00755 DRB1*11:03	HLA:HLA00867 DRB1*15:02:01	27	12
HLA:HLA00756 DRB1*11:04:01	HLA:HLA00867 DRB1*15:02:01	27	13
HLA:HLA01407 DRB1*12:01:02	HLA:HLA00867 DRB1*15:02:01	27	19
HLA:HLA00825 DRB1*13:27	HLA:HLA00867 DRB1*15:02:01	27	13
HLA:HLA00833 DRB1*14:01:01	HLA:HLA00867 DRB1*15:02:01	27	17
HLA:HLA00836 DRB1*14:04	HLA:HLA00867 DRB1*15:02:01	27	16
HLA:HLA00671 DRB1*03:01:01:01	HLA:HLA00870 DRB1*15:03:01:01	27	15
HLA:HLA03483 DRB1*03:01:01:02	HLA:HLA00870 DRB1*15:03:01:01	27	15
HLA:HLA00678 DRB1*03:06	HLA:HLA00870 DRB1*15:03:01:01	27	16
HLA:HLA02257 DRB1*08:01:03	HLA:HLA00870 DRB1*15:03:01:01	27	18
HLA:HLA00727 DRB1*08:03:02	HLA:HLA00870 DRB1*15:03:01:01	27	17
HLA:HLA00735 DRB1*08:09	HLA:HLA00870 DRB1*15:03:01:01	27	17
HLA:HLA00744 DRB1*08:18	HLA:HLA00870 DRB1*15:03:01:01	27	15
HLA:HLA00749 DRB1*09:01:02	HLA:HLA00870 DRB1*15:03:01:01	27	24
HLA:HLA00751 DRB1*11:01:01	HLA:HLA00870 DRB1*15:03:01:01	27	14
HLA:HLA00769 DRB1*11:15	HLA:HLA00870 DRB1*15:03:01:01	27	16
HLA:HLA00843 DRB1*14:11	HLA:HLA00870 DRB1*15:03:01:01	27	14
HLA:HLA00671 DRB1*03:01:01:01	HLA:HLA03454 DRB1*15:03:01:02	27	15
HLA:HLA03483 DRB1*03:01:01:02	HLA:HLA03454 DRB1*15:03:01:02	27	15
HLA:HLA00678 DRB1*03:06	HLA:HLA03454 DRB1*15:03:01:02	27	16
HLA:HLA02257 DRB1*08:01:03	HLA:HLA03454 DRB1*15:03:01:02	27	18
HLA:HLA00727 DRB1*08:03:02	HLA:HLA03454 DRB1*15:03:01:02	27	17
HLA:HLA00735 DRB1*08:09	HLA:HLA03454 DRB1*15:03:01:02	27	17
HLA:HLA00744 DRB1*08:18	HLA:HLA03454 DRB1*15:03:01:02	27	15
HLA:HLA00749 DRB1*09:01:02	HLA:HLA03454 DRB1*15:03:01:02	27	24
HLA:HLA00751 DRB1*11:01:01	HLA:HLA03454 DRB1*15:03:01:02	27	14
HLA:HLA00769 DRB1*11:15	HLA:HLA03454 DRB1*15:03:01:02	27	16
HLA:HLA00843 DRB1*14:11	HLA:HLA03454 DRB1*15:03:01:02	27	14
HLA:HLA00671 DRB1*03:01:01:01	HLA:HLA00871 DRB1*15:04	27	14
HLA:HLA03483 DRB1*03:01:01:02	HLA:HLA00871 DRB1*15:04	27	14
HLA:HLA00678 DRB1*03:06	HLA:HLA00871 DRB1*15:04	27	15
HLA:HLA02257 DRB1*08:01:03	HLA:HLA00871 DRB1*15:04	27	16
HLA:HLA00727 DRB1*08:03:02	HLA:HLA00871 DRB1*15:04	27	17
HLA:HLA00735 DRB1*08:09	HLA:HLA00871 DRB1*15:04	27	15
HLA:HLA00744 DRB1*08:18	HLA:HLA00871 DRB1*15:04	27	15
HLA:HLA00749 DRB1*09:01:02	HLA:HLA00871 DRB1*15:04	27	23
HLA:HLA00751 DRB1*11:01:01	HLA:HLA00871 DRB1*15:04	27	12
HLA:HLA00769 DRB1*11:15	HLA:HLA00871 DRB1*15:04	27	14
HLA:HLA00843 DRB1*14:11	HLA:HLA00871 DRB1*15:04	27	13
HLA:HLA00688 DRB1*04:03:01	HLA:HLA00876 DRB1*16:01:01	27	13
HLA:HLA00689 DRB1*04:04:01	HLA:HLA00876 DRB1*16:01:01	27	12
HLA:HLA00692 DRB1*04:06:01	HLA:HLA00876 DRB1*16:01:01	27	12
HLA:HLA00688 DRB1*04:03:01	HLA:HLA00878 DRB1*16:02:01	27	12
HLA:HLA00689 DRB1*04:04:01	HLA:HLA00878 DRB1*16:02:01	27	11
HLA:HLA00692 DRB1*04:06:01	HLA:HLA00878 DRB1*16:02:01	27	11
HLA:HLA00671 DRB1*03:01:01:01	HLA:HLA00685 DRB1*04:01:01	27	10
HLA:HLA03483 DRB1*03:01:01:02	HLA:HLA00685 DRB1*04:01:01	27	10
HLA:HLA00678 DRB1*03:06	HLA:HLA00685 DRB1*04:01:01	27	9
HLA:HLA00671 DRB1*03:01:01:01	HLA:HLA00687 DRB1*04:02	27	13
HLA:HLA03483 DRB1*03:01:01:02	HLA:HLA00687 DRB1*04:02	27	13
HLA:HLA00678 DRB1*03:06	HLA:HLA00687 DRB1*04:02	27	12
HLA:HLA00672 DRB1*03:01:02	HLA:HLA00688 DRB1*04:03:01	27	10
HLA:HLA00672 DRB1*03:01:02	HLA:HLA00689 DRB1*04:04:01	27	10
HLA:HLA00672 DRB1*03:01:02	HLA:HLA00692 DRB1*04:06:01	27	11
HLA:HLA00671 DRB1*03:01:01:01	HLA:HLA02172 DRB1*04:06:02	27	11
HLA:HLA03483 DRB1*03:01:01:02	HLA:HLA02172 DRB1*04:06:02	27	11
HLA:HLA00678 DRB1*03:06	HLA:HLA02172 DRB1*04:06:02	27	10
HLA:HLA00671 DRB1*03:01:01:01	HLA:HLA00693 DRB1*04:07:01	27	11
HLA:HLA03483 DRB1*03:01:01:02	HLA:HLA00693 DRB1*04:07:01	27	11
HLA:HLA00678 DRB1*03:06	HLA:HLA00693 DRB1*04:07:01	27	10
HLA:HLA00671 DRB1*03:01:01:01	HLA:HLA00694 DRB1*04:08:01	27	11
HLA:HLA03483 DRB1*03:01:01:02	HLA:HLA00694 DRB1*04:08:01	27	11
HLA:HLA00678 DRB1*03:06	HLA:HLA00694 DRB1*04:08:01	27	10
HLA:HLA00671 DRB1*03:01:01:01	HLA:HLA00696 DRB1*04:10	27	12
HLA:HLA03483 DRB1*03:01:01:02	HLA:HLA00696 DRB1*04:10	27	12
HLA:HLA00678 DRB1*03:06	HLA:HLA00696 DRB1*04:10	27	11
HLA:HLA00671 DRB1*03:01:01:01	HLA:HLA00697 DRB1*04:11	27	12
HLA:HLA03483 DRB1*03:01:01:02	HLA:HLA00697 DRB1*04:11	27	12
HLA:HLA00678 DRB1*03:06	HLA:HLA00697 DRB1*04:11	27	11
HLA:HLA02172 DRB1*04:06:02	HLA:HLA02257 DRB1*08:01:03	27	13
HLA:HLA02172 DRB1*04:06:02	HLA:HLA00727 DRB1*08:03:02	27	13
HLA:HLA02172 DRB1*04:06:02	HLA:HLA00735 DRB1*08:09	27	12
HLA:HLA00696 DRB1*04:10	HLA:HLA00735 DRB1*08:09	27	14
HLA:HLA00697 DRB1*04:11	HLA:HLA00735 DRB1*08:09	27	14
HLA:HLA02172 DRB1*04:06:02	HLA:HLA00744 DRB1*08:18	27	12
HLA:HLA00672 DRB1*03:01:02	HLA:HLA00750 DRB1*10:01:01	27	14
HLA:HLA02172 DRB1*04:06:02	HLA:HLA00752 DRB1*11:01:02	27	11
HLA:HLA00696 DRB1*04:10	HLA:HLA00752 DRB1*11:01:02	27	11
HLA:HLA00697 DRB1*04:11	HLA:HLA00752 DRB1*11:01:02	27	12
HLA:HLA00665 DRB1*01:02:01	HLA:HLA00755 DRB1*11:03	27	15
HLA:HLA00665 DRB1*01:02:01	HLA:HLA00756 DRB1*11:04:01	27	13
HLA:HLA00665 DRB1*01:02:01	HLA:HLA02157 DRB1*11:11:02	27	16
HLA:HLA00688 DRB1*04:03:01	HLA:HLA01407 DRB1*12:01:02	27	18
HLA:HLA00689 DRB1*04:04:01	HLA:HLA01407 DRB1*12:01:02	27	17
HLA:HLA00690 DRB1*04:05:01	HLA:HLA01407 DRB1*12:01:02	27	19
HLA:HLA00692 DRB1*04:06:01	HLA:HLA01407 DRB1*12:01:02	27	18
HLA:HLA00695 DRB1*04:09	HLA:HLA01407 DRB1*12:01:02	27	20
HLA:HLA00665 DRB1*01:02:01	HLA:HLA00798 DRB1*13:02:01	27	18
HLA:HLA00665 DRB1*01:02:01	HLA:HLA00802 DRB1*13:05:01	27	16
HLA:HLA00665 DRB1*01:02:01	HLA:HLA00810 DRB1*13:12:01	27	15
HLA:HLA00749 DRB1*09:01:02	HLA:HLA00810 DRB1*13:12:01	27	18
HLA:HLA00690 DRB1*04:05:01	HLA:HLA00825 DRB1*13:27	27	15
HLA:HLA00695 DRB1*04:09	HLA:HLA00825 DRB1*13:27	27	14
HLA:HLA00665 DRB1*01:02:01	HLA:HLA01724 DRB1*13:56	27	15
HLA:HLA00665 DRB1*01:02:01	HLA:HLA00833 DRB1*14:01:01	27	14
HLA:HLA00685 DRB1*04:01:01	HLA:HLA00833 DRB1*14:01:01	27	12
HLA:HLA02172 DRB1*04:06:02	HLA:HLA00833 DRB1*14:01:01	27	9
HLA:HLA00693 DRB1*04:07:01	HLA:HLA00833 DRB1*14:01:01	27	10
HLA:HLA00694 DRB1*04:08:01	HLA:HLA00833 DRB1*14:01:01	27	11
HLA:HLA00696 DRB1*04:10	HLA:HLA00833 DRB1*14:01:01	27	11
HLA:HLA00697 DRB1*04:11	HLA:HLA00833 DRB1*14:01:01	27	10
HLA:HLA00665 DRB1*01:02:01	HLA:HLA00836 DRB1*14:04	27	15
HLA:HLA00685 DRB1*04:01:01	HLA:HLA00836 DRB1*14:04	27	12
HLA:HLA02172 DRB1*04:06:02	HLA:HLA00836 DRB1*14:04	27	9
HLA:HLA00693 DRB1*04:07:01	HLA:HLA00836 DRB1*14:04	27	10
HLA:HLA00694 DRB1*04:08:01	HLA:HLA00836 DRB1*14:04	27	11
HLA:HLA00696 DRB1*04:10	HLA:HLA00836 DRB1*14:04	27	11
HLA:HLA00697 DRB1*04:11	HLA:HLA00836 DRB1*14:04	27	10
HLA:HLA00665 DRB1*01:02:01	HLA:HLA00837 DRB1*14:05:01	27	12
HLA:HLA00685 DRB1*04:01:01	HLA:HLA00837 DRB1*14:05:01	27	10
HLA:HLA02172 DRB1*04:06:02	HLA:HLA00837 DRB1*14:05:01	27	7
HLA:HLA00693 DRB1*04:07:01	HLA:HLA00837 DRB1*14:05:01	27	8
HLA:HLA00694 DRB1*04:08:01	HLA:HLA00837 DRB1*14:05:01	27	9
HLA:HLA00696 DRB1*04:10	HLA:HLA00837 DRB1*14:05:01	27	10
HLA:HLA00697 DRB1*04:11	HLA:HLA00837 DRB1*14:05:01	27	9
HLA:HLA00665 DRB1*01:02:01	HLA:HLA00839 DRB1*14:07:01	27	15
HLA:HLA02172 DRB1*04:06:02	HLA:HLA00839 DRB1*14:07:01	27	10
HLA:HLA00696 DRB1*04:10	HLA:HLA00839 DRB1*14:07:01	27	12
HLA:HLA00697 DRB1*04:11	HLA:HLA00839 DRB1*14:07:01	27	11
HLA:HLA00688 DRB1*04:03:01	HLA:HLA00843 DRB1*14:11	27	7
HLA:HLA00689 DRB1*04:04:01	HLA:HLA00843 DRB1*14:11	27	8
HLA:HLA00692 DRB1*04:06:01	HLA:HLA00843 DRB1*14:11	27	7
HLA:HLA00750 DRB1*10:01:01	HLA:HLA00843 DRB1*14:11	27	11
HLA:HLA00690 DRB1*04:05:01	HLA:HLA02371 DRB1*14:54	27	12
HLA:HLA00695 DRB1*04:09	HLA:HLA02371 DRB1*14:54	27	13
HLA:HLA00749 DRB1*09:01:02	HLA:HLA00876 DRB1*16:01:01	27	20
HLA:HLA00837 DRB1*14:05:01	HLA:HLA00876 DRB1*16:01:01	27	13
HLA:HLA02371 DRB1*14:54	HLA:HLA00876 DRB1*16:01:01	27	15
HLA:HLA00749 DRB1*09:01:02	HLA:HLA00878 DRB1*16:02:01	27	21
HLA:HLA00837 DRB1*14:05:01	HLA:HLA00878 DRB1*16:02:01	27	12
HLA:HLA02371 DRB1*14:54	HLA:HLA00878 DRB1*16:02:01	27	14
HLA:HLA00665 DRB1*01:02:01	HLA:HLA00719 DRB1*07:01:01:01	28	18
HLA:HLA00665 DRB1*01:02:01	HLA:HLA03486 DRB1*07:01:01:02	28	18
HLA:HLA00665 DRB1*01:02:01	HLA:HLA02017 DRB1*07:09	28	17
HLA:HLA00687 DRB1*04:02	HLA:HLA00833 DRB1*14:01:01	28	15
HLA:HLA00687 DRB1*04:02	HLA:HLA00836 DRB1*14:04	28	15
HLA:HLA00687 DRB1*04:02	HLA:HLA00837 DRB1*14:05:01	28	13

Allele 1	Allele 2	m	K_B
HLA:HLA00687 DRB1*04:02	HLA:HLA00839 DRB1*14:07:01	28	16
HLA:HLA00719 DRB1*07:01:01:01	HLA:HLA01693 DRB1*14:46	28	19
HLA:HLA03486 DRB1*07:01:01:02	HLA:HLA01693 DRB1*14:46	28	19
HLA:HLA02017 DRB1*07:09	HLA:HLA01693 DRB1*14:46	28	18
HLA:HLA00672 DRB1*03:01:02	HLA:HLA00865 DRB1*15:01:01:01	28	14
HLA:HLA00724 DRB1*08:02:01	HLA:HLA00865 DRB1*15:01:01:01	28	15
HLA:HLA00739 DRB1*08:13	HLA:HLA00865 DRB1*15:01:01:01	28	15
HLA:HLA00752 DRB1*11:01:02	HLA:HLA00865 DRB1*15:01:01:01	28	13
HLA:HLA00672 DRB1*03:01:02	HLA:HLA03453 DRB1*15:01:01:02	28	14
HLA:HLA00724 DRB1*08:02:01	HLA:HLA03453 DRB1*15:01:01:02	28	15
HLA:HLA00739 DRB1*08:13	HLA:HLA03453 DRB1*15:01:01:02	28	15
HLA:HLA00752 DRB1*11:01:02	HLA:HLA03453 DRB1*15:01:01:02	28	13
HLA:HLA00671 DRB1*03:01:01:01	HLA:HLA00867 DRB1*15:02:01	28	15
HLA:HLA03483 DRB1*03:01:01:02	HLA:HLA00867 DRB1*15:02:01	28	15
HLA:HLA00678 DRB1*03:06	HLA:HLA00867 DRB1*15:02:01	28	16
HLA:HLA00843 DRB1*14:11	HLA:HLA00867 DRB1*15:02:01	28	14
HLA:HLA00672 DRB1*03:01:02	HLA:HLA00870 DRB1*15:03:01:01	28	15
HLA:HLA00724 DRB1*08:02:01	HLA:HLA00870 DRB1*15:03:01:01	28	16
HLA:HLA00739 DRB1*08:13	HLA:HLA00870 DRB1*15:03:01:01	28	16
HLA:HLA00752 DRB1*11:01:02	HLA:HLA00870 DRB1*15:03:01:01	28	14
HLA:HLA00672 DRB1*03:01:02	HLA:HLA03454 DRB1*15:03:01:02	28	15
HLA:HLA00724 DRB1*08:02:01	HLA:HLA03454 DRB1*15:03:01:02	28	16
HLA:HLA00739 DRB1*08:13	HLA:HLA03454 DRB1*15:03:01:02	28	16
HLA:HLA00752 DRB1*11:01:02	HLA:HLA03454 DRB1*15:03:01:02	28	14
HLA:HLA00672 DRB1*03:01:02	HLA:HLA00871 DRB1*15:04	28	14
HLA:HLA00724 DRB1*08:02:01	HLA:HLA00871 DRB1*15:04	28	14
HLA:HLA00739 DRB1*08:13	HLA:HLA00871 DRB1*15:04	28	15
HLA:HLA00752 DRB1*11:01:02	HLA:HLA00871 DRB1*15:04	28	12
HLA:HLA02172 DRB1*04:06:02	HLA:HLA00876 DRB1*16:01:01	28	12
HLA:HLA02172 DRB1*04:06:02	HLA:HLA00878 DRB1*16:02:01	28	11
HLA:HLA00672 DRB1*03:01:02	HLA:HLA00685 DRB1*04:01:01	28	10
HLA:HLA00672 DRB1*03:01:02	HLA:HLA00687 DRB1*04:02	28	13
HLA:HLA00671 DRB1*03:01:01:01	HLA:HLA00690 DRB1*04:05:01	28	13
HLA:HLA03483 DRB1*03:01:01:02	HLA:HLA00690 DRB1*04:05:01	28	13
HLA:HLA00678 DRB1*03:06	HLA:HLA00690 DRB1*04:05:01	28	12
HLA:HLA00672 DRB1*03:01:02	HLA:HLA02172 DRB1*04:06:02	28	11
HLA:HLA00672 DRB1*03:01:02	HLA:HLA00693 DRB1*04:07:01	28	11
HLA:HLA00672 DRB1*03:01:02	HLA:HLA00694 DRB1*04:08:01	28	11
HLA:HLA00671 DRB1*03:01:01:01	HLA:HLA00695 DRB1*04:09	28	12
HLA:HLA03483 DRB1*03:01:01:02	HLA:HLA00695 DRB1*04:09	28	12
HLA:HLA00678 DRB1*03:06	HLA:HLA00695 DRB1*04:09	28	11
HLA:HLA00672 DRB1*03:01:02	HLA:HLA00696 DRB1*04:10	28	12
HLA:HLA00672 DRB1*03:01:02	HLA:HLA00697 DRB1*04:11	28	12
HLA:HLA00665 DRB1*01:02:01	HLA:HLA00724 DRB1*08:02:01	28	16
HLA:HLA00665 DRB1*01:02:01	HLA:HLA00739 DRB1*08:13	28	15
HLA:HLA00665 DRB1*01:02:01	HLA:HLA00751 DRB1*11:01:01	28	14
HLA:HLA00665 DRB1*01:02:01	HLA:HLA00769 DRB1*11:15	28	16
HLA:HLA00685 DRB1*04:01:01	HLA:HLA01407 DRB1*12:01:02	28	19
HLA:HLA02172 DRB1*04:06:02	HLA:HLA01407 DRB1*12:01:02	28	18
HLA:HLA00693 DRB1*04:07:01	HLA:HLA01407 DRB1*12:01:02	28	19
HLA:HLA00694 DRB1*04:08:01	HLA:HLA01407 DRB1*12:01:02	28	18
HLA:HLA00665 DRB1*01:02:01	HLA:HLA00825 DRB1*13:27	28	17
HLA:HLA00690 DRB1*04:05:01	HLA:HLA00833 DRB1*14:01:01	28	12
HLA:HLA00695 DRB1*04:09	HLA:HLA00833 DRB1*14:01:01	28	13
HLA:HLA00690 DRB1*04:05:01	HLA:HLA00836 DRB1*14:04	28	12
HLA:HLA00695 DRB1*04:09	HLA:HLA00836 DRB1*14:04	28	13
HLA:HLA00690 DRB1*04:05:01	HLA:HLA00837 DRB1*14:05:01	28	11
HLA:HLA00695 DRB1*04:09	HLA:HLA00837 DRB1*14:05:01	28	12
HLA:HLA00665 DRB1*01:02:01	HLA:HLA00843 DRB1*14:11	28	13
HLA:HLA00685 DRB1*04:01:01	HLA:HLA00843 DRB1*14:11	28	10
HLA:HLA02172 DRB1*04:06:02	HLA:HLA00843 DRB1*14:11	28	7
HLA:HLA00693 DRB1*04:07:01	HLA:HLA00843 DRB1*14:11	28	8
HLA:HLA00694 DRB1*04:08:01	HLA:HLA00843 DRB1*14:11	28	9
HLA:HLA00696 DRB1*04:10	HLA:HLA00843 DRB1*14:11	28	10
HLA:HLA00697 DRB1*04:11	HLA:HLA00843 DRB1*14:11	28	9
HLA:HLA00833 DRB1*14:01:01	HLA:HLA00876 DRB1*16:01:01	28	15
HLA:HLA00836 DRB1*14:04	HLA:HLA00876 DRB1*16:01:01	28	14
HLA:HLA00833 DRB1*14:01:01	HLA:HLA00878 DRB1*16:02:01	28	14
HLA:HLA00836 DRB1*14:04	HLA:HLA00878 DRB1*16:02:01	28	13

Table S2 Parallel substitution sites in the PBR among allele pairs over all phases

			24 sites in the PBR (Brown *et al*. 1993) and 3 sites in peptide-binding pockets (Stern *et al*. 1994)																										
	m	No. of allele pairs	9	11	13	28	30	32	37	38	47	56	57	60	61	65	67	68	70	71	74	78	81	82	85	86	88	89	90
**Phase I**	0	32																											
	1	58																											
	2	40																											
	3	32																											
	4	39																											
	5	30																											
	6	45																											
	7	58																											
	8	64																											
	9	50																											
	10	30																											
	11	24																											
	12	24																											
	13	9																											
	14																												
	15																												
	16																												
	17																												
	18																												
	19	2																											
**Phase II**	20	16																											
	21	18																											
	22	27																											
	23	54																											
	24	102																											
	25	185																											
	26	214																											
	27	160																											
	28	75																											
**Phase III**	29	29																											
	30	24																											
**Phase IV**	31	25																											
	32	31																											
	33	28																											
	34	15																											

Red rectangle represents the parallel substitution site of the PBR in allele pairs which have same *m* value. Yellow rectangle represents the segregating site of the PBR in allele pairs which have same *m* value.

Table S3 The specific pathogens bound by HLA-DRB1 allelic lineages only with fast or slow PBR substitution rate

HLA-DRB1 molecule	Source Organism ID	Source Organism Name	PBR substitution rate
HLA-DRB1*03	10580	Human papillomavirus type 11	Fast
HLA-DRB1*04	236	*Brucella ovis*	Slow
	1358	*Lactococcus lactis*	
	10254	Vaccinia virus WR	
	11309	Herpes simplex virus (type 1 / strain SC16)	
	211044	Influenza A virus (A/Puerto Rico/8/1934(H1N1))	
	243160	*Burkholderia mallei* ATCC 23344	
	381512	Influenza A virus (A/New Caledonia/20/1999(H1N1))	
	381513	Influenza A virus (A/Panama/2007/1999(H3N2))	
	641501	Influenza A virus (A/California/04/2009(H1N1))	
HLA-DRB1*15	1313	*Streptococcus pneumoniae*	
	1423	*Bacillus subtilis*	
	1764	*Mycobacterium avium*	

The dataset of source organism bound to HLA-DRB1 molecules was acquired from the MHC binding assay in the IEDB database (*http://www.iedb.org/*).
