# Supplementary material for: Nonsynonymous Substitution Rate Heterogeneity in the Peptide-Binding Region Among Different HLA-DRB1 Lineages in Humans
Source: G3 (Bethesda). 2014 May 2;4(7):1217–26. doi: 10.1534/g3.114.011726 (PMC4455771; doi:10.1534/g3.114.011726)
Supplement: Supporting Information [file supp_g3.114.011726_FigureS1.pdf]

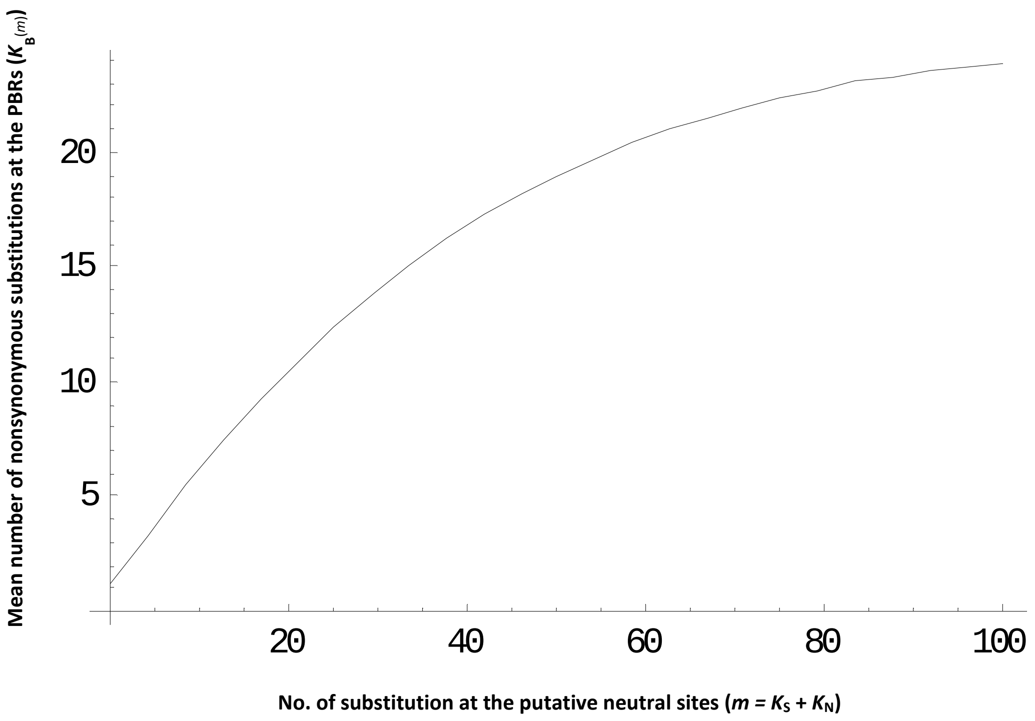

Figure S1 Expected distribution of $K_{B(m)}$ values. The pairwise mean number of nonsynonymous substitutions $K_{B(m)}$ in the PBR conditioned on the number of putative synonymous substitutions *m*. This conditional mean number is calculated on the basis of Equation 12 described in Takahata *et al*. (1992).
