# Supplementary material for: Nonsynonymous Substitution Rate Heterogeneity in the Peptide-Binding Region Among Different HLA-DRB1 Lineages in Humans
Source: G3 (Bethesda). 2014 May 2;4(7):1217–26. doi: 10.1534/g3.114.011726 (PMC4455771; doi:10.1534/g3.114.011726)
Supplement: Supporting Information [file supp_g3.114.011726_FigureS2.pdf]

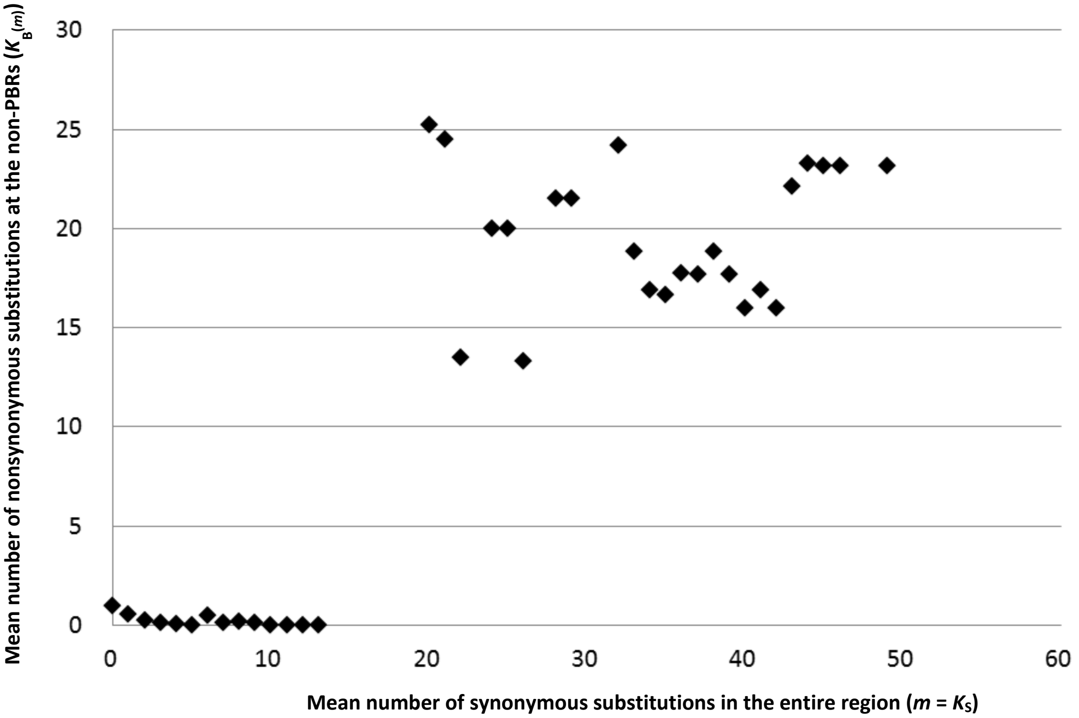


**Figure S2 The relationship of level of amino acid substitutions at the PBR ($K_{B(m)}$) and coalescence time ($m = K_S$) of alleles for 24 rat *RT1-Db1* (*HLA-DRB1* ortholog) alleles.** The ordinate axis represents the mean number of nonsynonymous substitutions at the PBR among allele pairs ($K_{B(m)}$). The abscissa axis represents the number of synonymous substitutions over the entire region ($m = K_S$).
