# Supplementary material for: Nonsynonymous Substitution Rate Heterogeneity in the Peptide-Binding Region Among Different HLA-DRB1 Lineages in Humans
Source: G3 (Bethesda). 2014 May 2;4(7):1217–26. doi: 10.1534/g3.114.011726 (PMC4455771; doi:10.1534/g3.114.011726)
Supplement: Supporting Information [file supp_g3.114.011726_FigureS3.pdf]

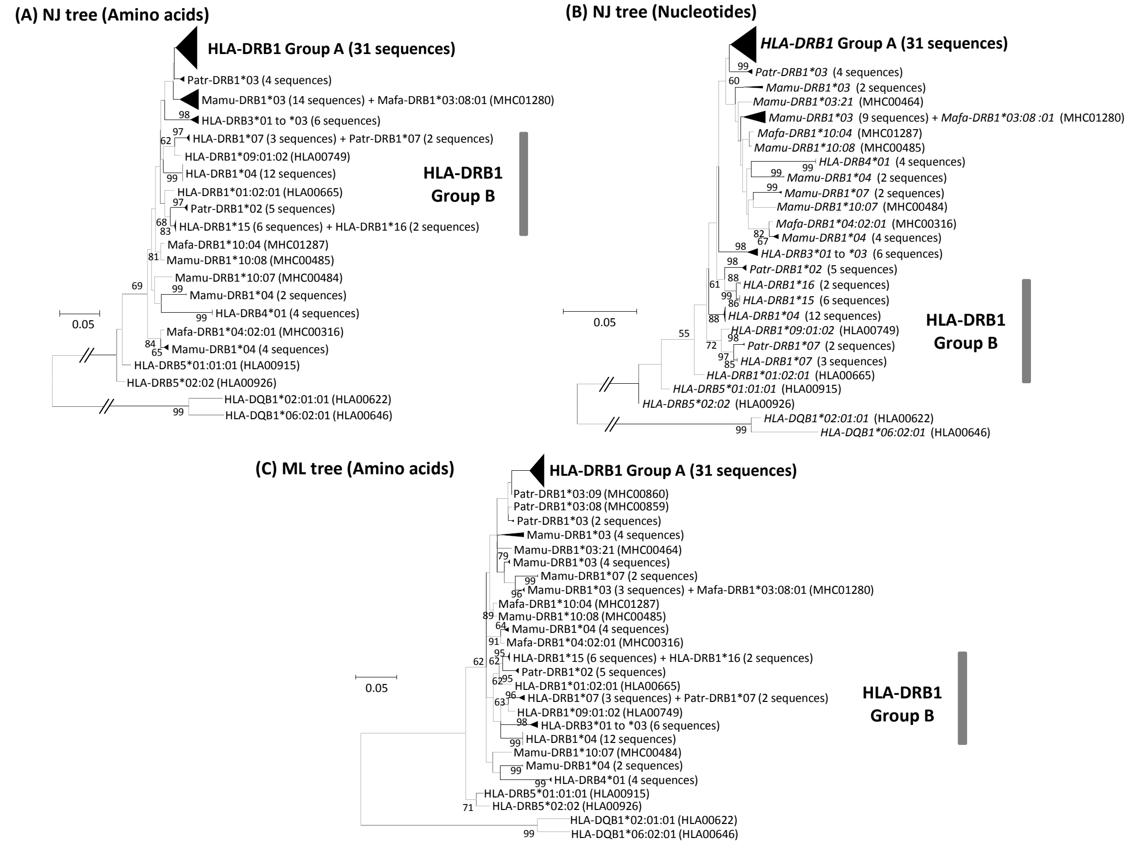

Figure S3 Neighbor-joining (NJ) and maximum likelihood (ML) trees based on nucleotide and amino acid sequences in the non-PBRs of *HLA-DRB1* alleles. (A) NJ tree of amino acid sequences of *DRB1* alleles on the basis of the JTT model with gamma distribution, (B) NJ tree of nucleotide sequences on the basis of the Tamura 3 parameters model with gamma distribution, (C) ML tree of amino acid sequences on the basis of the JTT model. The tree construction and estimation of the best fit substitution model were performed by using the MEGA v5.10 software. Only bootstrap values over 50% are shown in this figure. Two *HLA-DQB1* sequences are used as the outgroup. *HLA*, humans; *Patr*, chimpanzees; *Mamu*, rhesus monkeys; *Mafa*, crab-eating macaques. IMGT/HLA and IPD Accession Numbers are in parentheses.
