# Supplementary material for: Nonsynonymous Substitution Rate Heterogeneity in the Peptide-Binding Region Among Different HLA-DRB1 Lineages in Humans
Source: G3 (Bethesda). 2014 May 2;4(7):1217–26. doi: 10.1534/g3.114.011726 (PMC4455771; doi:10.1534/g3.114.011726)
Supplement: Supporting Information [file supp_g3.114.011726_FigureS4.pdf]

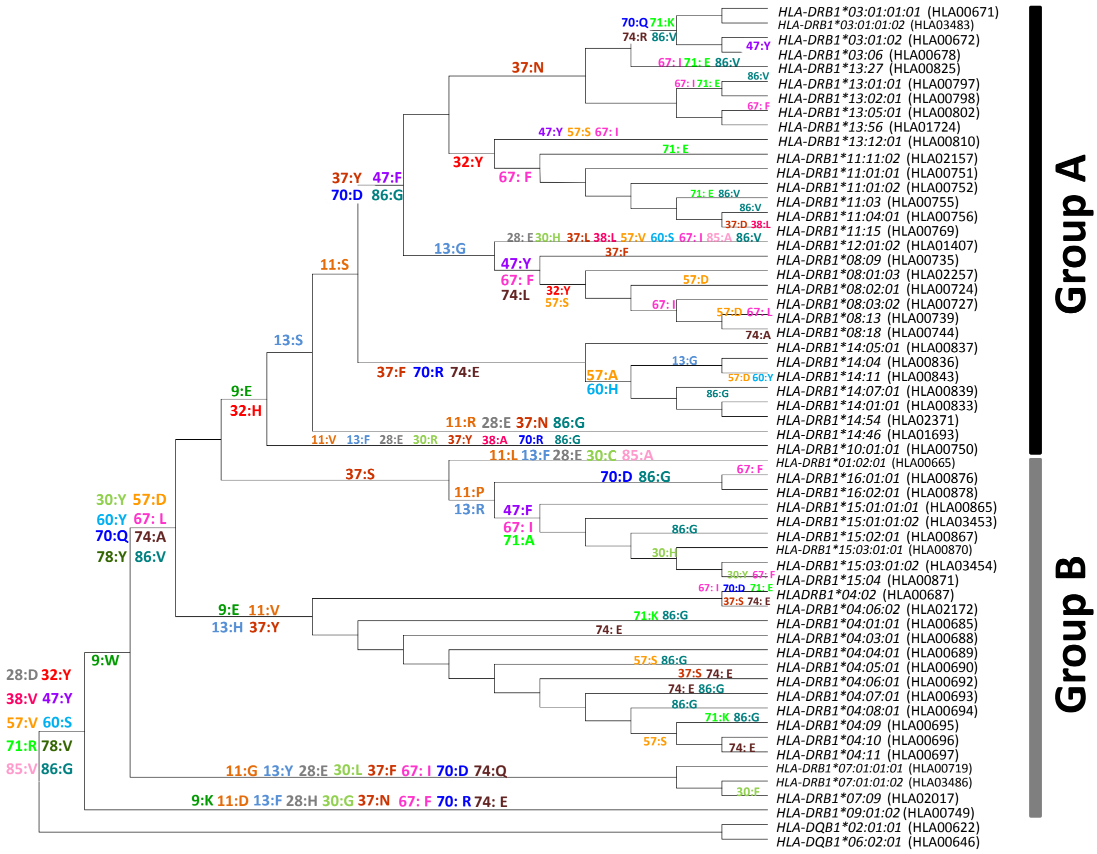

Figure S4 Maximum likelihood tree with the HKY model based on nucleotide sequences (690 bp) in the non-PBRs of *HLA-DRB1* alleles. Characters on branches indicate amino acid at each PBR position. Arrows represent the shared same amino acids from the node to tips. IMGT/HLA Accession Numbers are in parentheses.
