# Supplementary material for: Nonsynonymous Substitution Rate Heterogeneity in the Peptide-Binding Region Among Different HLA-DRB1 Lineages in Humans
Source: G3 (Bethesda). 2014 May 2;4(7):1217–26. doi: 10.1534/g3.114.011726 (PMC4455771; doi:10.1534/g3.114.011726)
Supplement: Supporting Information [file supp_g3.114.011726_FigureS5.pdf]

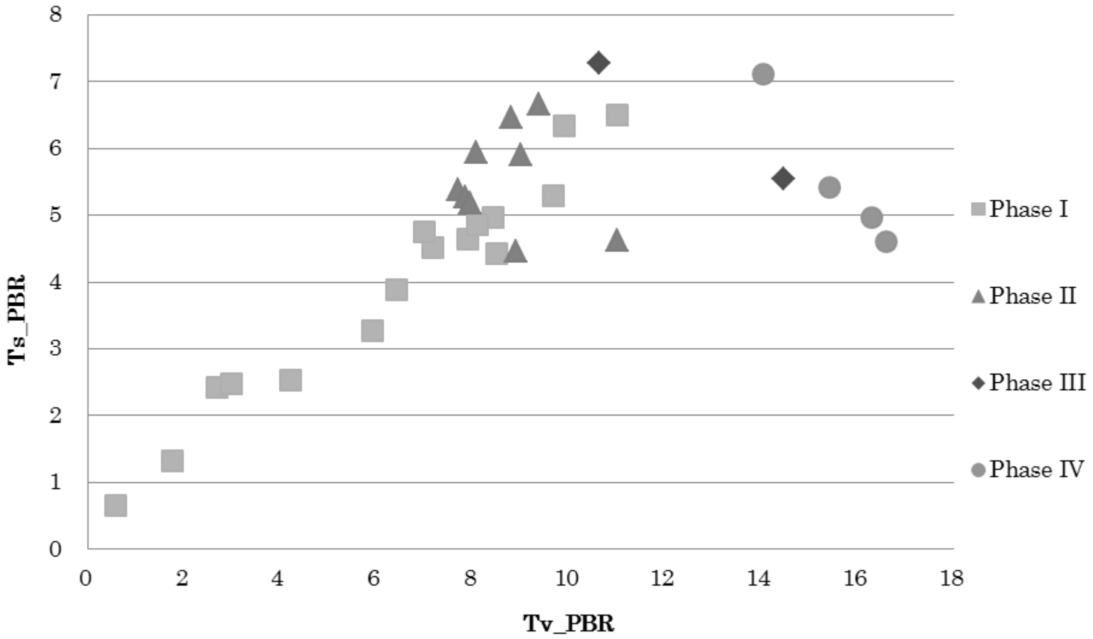

Figure S5 The number of transition and transversion substitutions at the PBR in *HLA-DRB1* alleles. The abscissa axis represents the mean number of transition substitutions at the PBR among allele pairs. The ordinate axis represents the mean number of transversion substitutions at the PBR.
