# Supplementary material for: Nonsynonymous Substitution Rate Heterogeneity in the Peptide-Binding Region Among Different HLA-DRB1 Lineages in Humans
Source: G3 (Bethesda). 2014 May 2;4(7):1217–26. doi: 10.1534/g3.114.011726 (PMC4455771; doi:10.1534/g3.114.011726)
Supplement: Supporting Information [file supp_g3.114.011726_FigureS8.pdf]

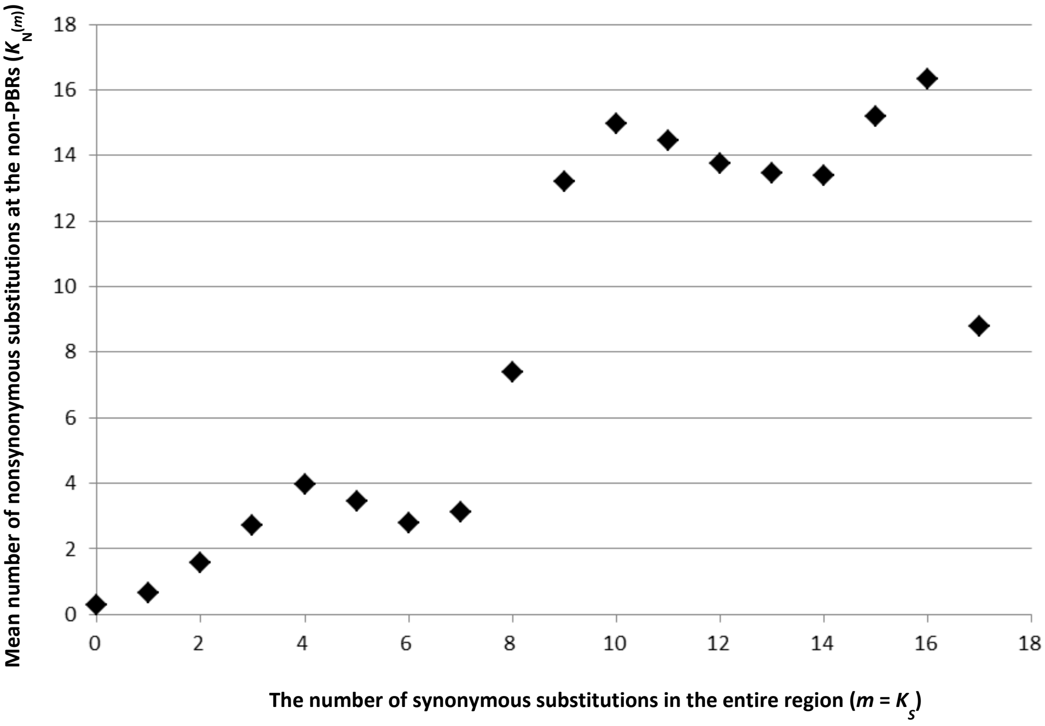

Figure S8 The level of amino acid substitutions at the non-PBR ($K_{N(m)}$) among *HLA-DRB1* allele pairs that share the same coalescence time ($m = K_S$). The ordinate axis represents the mean number of nonsynonymous substitutions at the non-PBR among allele pairs ($K_{N(m)}$). The abscissa axis represents the number of synonymous substitutions over the entire region ($m = K_S$).
