# Supplementary material for: Nonsynonymous Substitution Rate Heterogeneity in the Peptide-Binding Region Among Different HLA-DRB1 Lineages in Humans
Source: G3 (Bethesda). 2014 May 2;4(7):1217–26. doi: 10.1534/g3.114.011726 (PMC4455771; doi:10.1534/g3.114.011726)
Supplement: Supporting Information [file supp_g3.114.011726_FigureS9.pdf]

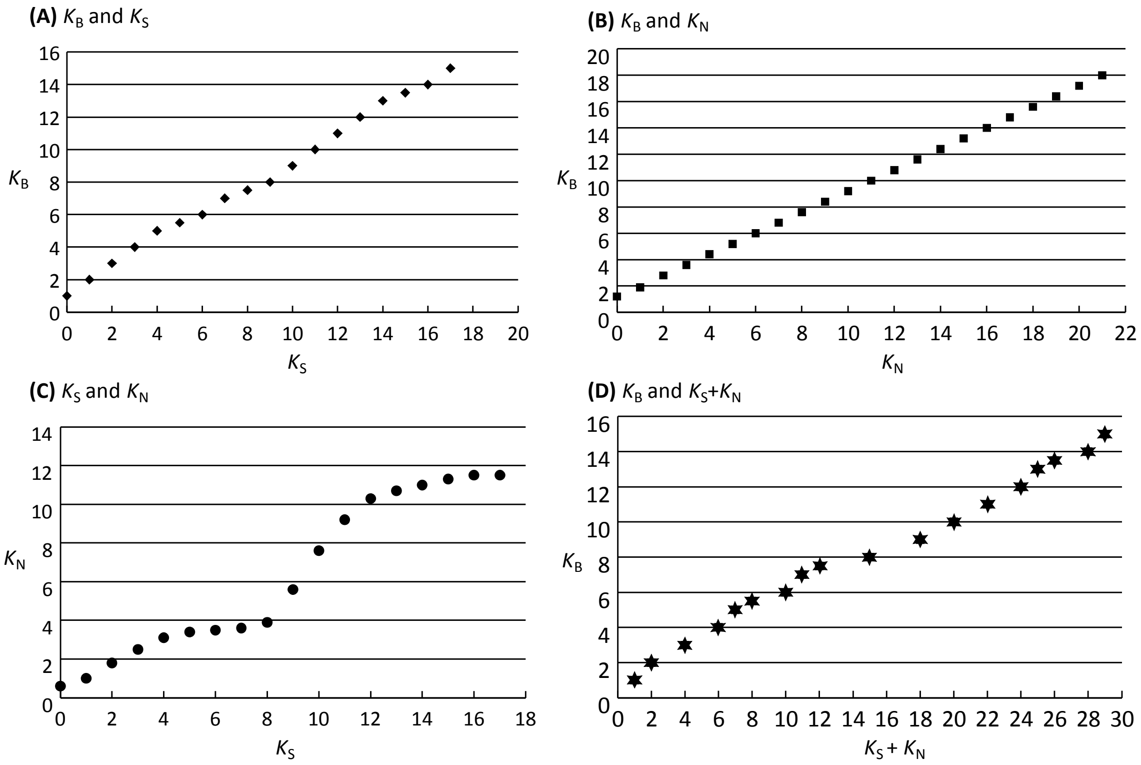

Figure S9 Relationships among K_S, K_N and K_B (A) Relationship between K_B and K_S, (B) Relationship between K_B and K_N, (C) Relationship between K_S and K_N, (D) Relationship between K_B and $K_S + K_N$. Even though K_N does not increase constantly with K_S, a proportional relationship was observed between K_B and $K_S + K_N$.
