# Supplementary material for: Nonsynonymous Substitution Rate Heterogeneity in the Peptide-Binding Region Among Different HLA-DRB1 Lineages in Humans
Source: G3 (Bethesda). 2014 May 2;4(7):1217–26. doi: 10.1534/g3.114.011726 (PMC4455771; doi:10.1534/g3.114.011726)
Supplement: Supporting Information [file supp_g3.114.011726_FigureS10.pdf]

(A)

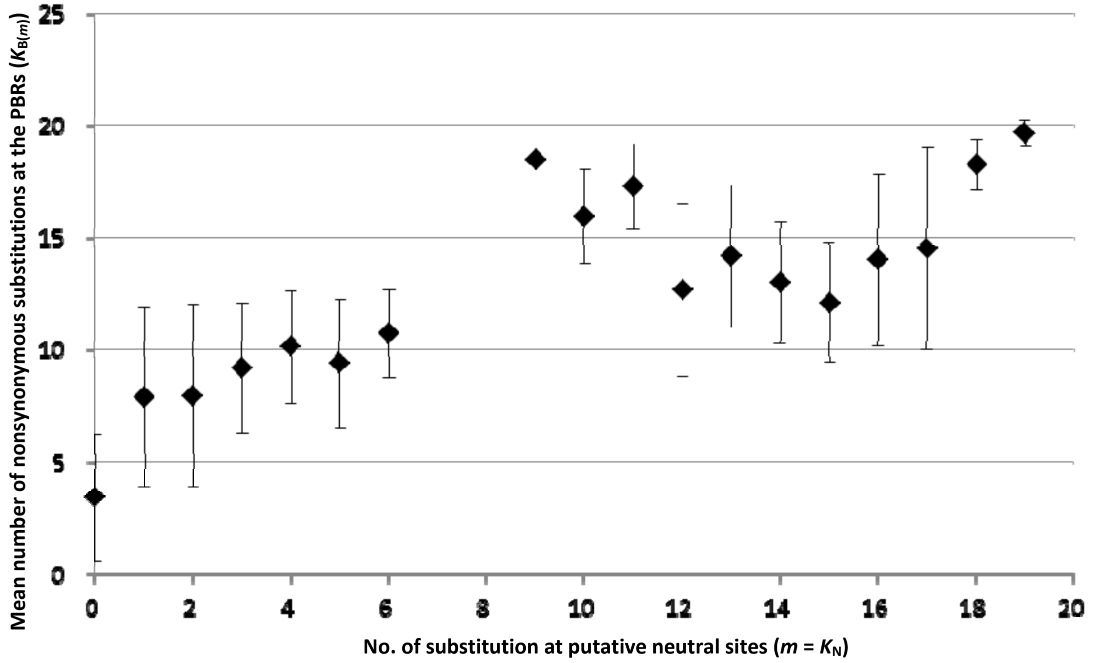

(B)

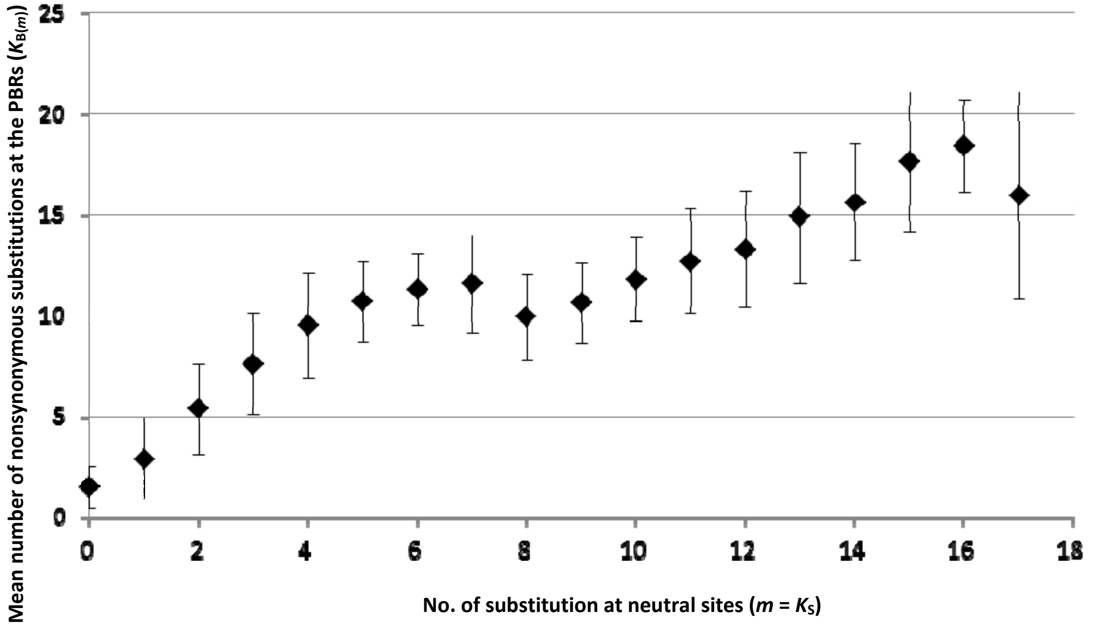

Figure S10 The mean number of nonsynonymous substitutions at the PBR ($K_{B(m)}$) among *HLA-DRB1* allele pairs that share the same K_N and K_S values. The ordinate axis represents the mean number of nonsynonymous substitutions at the PBR ($K_{B(m)}$). Abscissa axes represent K_N values (A) and K_S values (B). Error bars indicate the standard deviation from the mean.
