# Supplementary material for: Nonsynonymous Substitution Rate Heterogeneity in the Peptide-Binding Region Among Different HLA-DRB1 Lineages in Humans
Source: G3 (Bethesda). 2014 May 2;4(7):1217–26. doi: 10.1534/g3.114.011726 (PMC4455771; doi:10.1534/g3.114.011726)
Supplement: Supporting Information [file supp_g3.114.011726_TableS1.pdf]

Table S1 The allelic pairs consisting of phase II in the *HLA-DRB1* locus

Allele 1	Allele 2	m	K_B
HLA:HLA00685 DRB1*04:01:01	HLA:HLA00749 DRB1*09:01:02	20	17
HLA:HLA00693 DRB1*04:07:01	HLA:HLA00749 DRB1*09:01:02	20	15
HLA:HLA00694 DRB1*04:08:01	HLA:HLA00749 DRB1*09:01:02	20	16
HLA:HLA00696 DRB1*04:10	HLA:HLA00749 DRB1*09:01:02	20	18
HLA:HLA00697 DRB1*04:11	HLA:HLA00749 DRB1*09:01:02	20	17
HLA:HLA00665 DRB1*01:02:01	HLA:HLA00688 DRB1*04:03:01	20	10
HLA:HLA00665 DRB1*01:02:01	HLA:HLA00689 DRB1*04:04:01	20	9
HLA:HLA00665 DRB1*01:02:01	HLA:HLA00692 DRB1*04:06:01	20	9
HLA:HLA00685 DRB1*04:01:01	HLA:HLA01693 DRB1*14:46	20	8
HLA:HLA00693 DRB1*04:07:01	HLA:HLA01693 DRB1*14:46	20	8
HLA:HLA00694 DRB1*04:08:01	HLA:HLA01693 DRB1*14:46	20	7
HLA:HLA00665 DRB1*01:02:01	HLA:HLA00865 DRB1*15:01:01:01	20	11
HLA:HLA00665 DRB1*01:02:01	HLA:HLA03453 DRB1*15:01:01:02	20	11
HLA:HLA00665 DRB1*01:02:01	HLA:HLA00870 DRB1*15:03:01:01	20	12
HLA:HLA00665 DRB1*01:02:01	HLA:HLA03454 DRB1*15:03:01:02	20	12
HLA:HLA00665 DRB1*01:02:01	HLA:HLA00871 DRB1*15:04	20	11
HLA:HLA00688 DRB1*04:03:01	HLA:HLA00749 DRB1*09:01:02	21	16
HLA:HLA00689 DRB1*04:04:01	HLA:HLA00749 DRB1*09:01:02	21	17
HLA:HLA00692 DRB1*04:06:01	HLA:HLA00749 DRB1*09:01:02	21	17
HLA:HLA01693 DRB1*14:46	HLA:HLA00867 DRB1*15:02:01	21	14
HLA:HLA00665 DRB1*01:02:01	HLA:HLA00685 DRB1*04:01:01	21	11
HLA:HLA00665 DRB1*01:02:01	HLA:HLA00687 DRB1*04:02	21	14
HLA:HLA00665 DRB1*01:02:01	HLA:HLA02172 DRB1*04:06:02	21	9
HLA:HLA00665 DRB1*01:02:01	HLA:HLA00693 DRB1*04:07:01	21	11
HLA:HLA00665 DRB1*01:02:01	HLA:HLA00694 DRB1*04:08:01	21	10
HLA:HLA00665 DRB1*01:02:01	HLA:HLA00696 DRB1*04:10	21	11
HLA:HLA00665 DRB1*01:02:01	HLA:HLA00697 DRB1*04:11	21	12
HLA:HLA00688 DRB1*04:03:01	HLA:HLA01693 DRB1*14:46	21	9
HLA:HLA00689 DRB1*04:04:01	HLA:HLA01693 DRB1*14:46	21	8
HLA:HLA00690 DRB1*04:05:01	HLA:HLA01693 DRB1*14:46	21	9
HLA:HLA00692 DRB1*04:06:01	HLA:HLA01693 DRB1*14:46	21	10
HLA:HLA00695 DRB1*04:09	HLA:HLA01693 DRB1*14:46	21	10
HLA:HLA00750 DRB1*10:01:01	HLA:HLA01693 DRB1*14:46	21	8
HLA:HLA00665 DRB1*01:02:01	HLA:HLA00867 DRB1*15:02:01	21	12
HLA:HLA00690 DRB1*04:05:01	HLA:HLA00719 DRB1*07:01:01:01	22	19
HLA:HLA00695 DRB1*04:09	HLA:HLA00719 DRB1*07:01:01:01	22	20
HLA:HLA00690 DRB1*04:05:01	HLA:HLA03486 DRB1*07:01:01:02	22	19
HLA:HLA00695 DRB1*04:09	HLA:HLA03486 DRB1*07:01:01:02	22	20
HLA:HLA00690 DRB1*04:05:01	HLA:HLA02017 DRB1*07:09	22	18
HLA:HLA00695 DRB1*04:09	HLA:HLA02017 DRB1*07:09	22	19
HLA:HLA00665 DRB1*01:02:01	HLA:HLA00749 DRB1*09:01:02	22	19
HLA:HLA02172 DRB1*04:06:02	HLA:HLA00749 DRB1*09:01:02	22	17
HLA:HLA01693 DRB1*14:46	HLA:HLA00865 DRB1*15:01:01:01	22	15
HLA:HLA01693 DRB1*14:46	HLA:HLA03453 DRB1*15:01:01:02	22	15
HLA:HLA01693 DRB1*14:46	HLA:HLA00870 DRB1*15:03:01:01	22	16
HLA:HLA01693 DRB1*14:46	HLA:HLA03454 DRB1*15:03:01:02	22	16
HLA:HLA01693 DRB1*14:46	HLA:HLA00871 DRB1*15:04	22	15
HLA:HLA01693 DRB1*14:46	HLA:HLA00876 DRB1*16:01:01	22	13
HLA:HLA01693 DRB1*14:46	HLA:HLA00878 DRB1*16:02:01	22	12
HLA:HLA00665 DRB1*01:02:01	HLA:HLA00690 DRB1*04:05:01	22	12
HLA:HLA00665 DRB1*01:02:01	HLA:HLA00695 DRB1*04:09	22	13
HLA:HLA00687 DRB1*04:02	HLA:HLA00749 DRB1*09:01:02	22	20
HLA:HLA00687 DRB1*04:02	HLA:HLA00797 DRB1*13:01:01	22	7
HLA:HLA00690 DRB1*04:05:01	HLA:HLA00810 DRB1*13:12:01	22	7
HLA:HLA00695 DRB1*04:09	HLA:HLA00810 DRB1*13:12:01	22	8
HLA:HLA00687 DRB1*04:02	HLA:HLA01693 DRB1*14:46	22	13
HLA:HLA02172 DRB1*04:06:02	HLA:HLA01693 DRB1*14:46	22	10
HLA:HLA00696 DRB1*04:10	HLA:HLA01693 DRB1*14:46	22	10
HLA:HLA00697 DRB1*04:11	HLA:HLA01693 DRB1*14:46	22	11
HLA:HLA00665 DRB1*01:02:01	HLA:HLA00876 DRB1*16:01:01	22	11
HLA:HLA00665 DRB1*01:02:01	HLA:HLA00878 DRB1*16:02:01	22	10
HLA:HLA00685 DRB1*04:01:01	HLA:HLA00719 DRB1*07:01:01:01	23	19
HLA:HLA00693 DRB1*04:07:01	HLA:HLA00719 DRB1*07:01:01:01	23	17
HLA:HLA00694 DRB1*04:08:01	HLA:HLA00719 DRB1*07:01:01:01	23	18
HLA:HLA00696 DRB1*04:10	HLA:HLA00719 DRB1*07:01:01:01	23	20
HLA:HLA00697 DRB1*04:11	HLA:HLA00719 DRB1*07:01:01:01	23	19
HLA:HLA00685 DRB1*04:01:01	HLA:HLA03486 DRB1*07:01:01:02	23	19
HLA:HLA00693 DRB1*04:07:01	HLA:HLA03486 DRB1*07:01:01:02	23	17
HLA:HLA00694 DRB1*04:08:01	HLA:HLA03486 DRB1*07:01:01:02	23	18
HLA:HLA00696 DRB1*04:10	HLA:HLA03486 DRB1*07:01:01:02	23	20
HLA:HLA00697 DRB1*04:11	HLA:HLA03486 DRB1*07:01:01:02	23	19
HLA:HLA00685 DRB1*04:01:01	HLA:HLA02017 DRB1*07:09	23	18
HLA:HLA00693 DRB1*04:07:01	HLA:HLA02017 DRB1*07:09	23	16
HLA:HLA00694 DRB1*04:08:01	HLA:HLA02017 DRB1*07:09	23	17
HLA:HLA00696 DRB1*04:10	HLA:HLA02017 DRB1*07:09	23	19
HLA:HLA00697 DRB1*04:11	HLA:HLA02017 DRB1*07:09	23	18
HLA:HLA00750 DRB1*10:01:01	HLA:HLA00798 DRB1*13:02:01	23	15
HLA:HLA00750 DRB1*10:01:01	HLA:HLA00802 DRB1*13:05:01	23	13
HLA:HLA00750 DRB1*10:01:01	HLA:HLA00810 DRB1*13:12:01	23	14
HLA:HLA00750 DRB1*10:01:01	HLA:HLA01724 DRB1*13:56	23	12
HLA:HLA00798 DRB1*13:02:01	HLA:HLA00876 DRB1*16:01:01	23	13
HLA:HLA00802 DRB1*13:05:01	HLA:HLA00876 DRB1*16:01:01	23	10
HLA:HLA00810 DRB1*13:12:01	HLA:HLA00876 DRB1*16:01:01	23	10
HLA:HLA01724 DRB1*13:56	HLA:HLA00876 DRB1*16:01:01	23	11
HLA:HLA00798 DRB1*13:02:01	HLA:HLA00878 DRB1*16:02:01	23	13
HLA:HLA00802 DRB1*13:05:01	HLA:HLA00878 DRB1*16:02:01	23	11
HLA:HLA00810 DRB1*13:12:01	HLA:HLA00878 DRB1*16:02:01	23	10
HLA:HLA01724 DRB1*13:56	HLA:HLA00878 DRB1*16:02:01	23	10
HLA:HLA00687 DRB1*04:02	HLA:HLA00755 DRB1*11:03	23	6
HLA:HLA00687 DRB1*04:02	HLA:HLA00756 DRB1*11:04:01	23	8
HLA:HLA00685 DRB1*04:01:01	HLA:HLA02157 DRB1*11:11:02	23	9
HLA:HLA00687 DRB1*04:02	HLA:HLA02157 DRB1*11:11:02	23	7
HLA:HLA00693 DRB1*04:07:01	HLA:HLA02157 DRB1*11:11:02	23	11
HLA:HLA00694 DRB1*04:08:01	HLA:HLA02157 DRB1*11:11:02	23	10
HLA:HLA00688 DRB1*04:03:01	HLA:HLA00797 DRB1*13:01:01	23	13
HLA:HLA00689 DRB1*04:04:01	HLA:HLA00797 DRB1*13:01:01	23	12
HLA:HLA00692 DRB1*04:06:01	HLA:HLA00797 DRB1*13:01:01	23	14
HLA:HLA00685 DRB1*04:01:01	HLA:HLA00798 DRB1*13:02:01	23	11
HLA:HLA00687 DRB1*04:02	HLA:HLA00798 DRB1*13:02:01	23	8
HLA:HLA00693 DRB1*04:07:01	HLA:HLA00798 DRB1*13:02:01	23	13
HLA:HLA00694 DRB1*04:08:01	HLA:HLA00798 DRB1*13:02:01	23	12
HLA:HLA00685 DRB1*04:01:01	HLA:HLA00802 DRB1*13:05:01	23	11
HLA:HLA00687 DRB1*04:02	HLA:HLA00802 DRB1*13:05:01	23	11
HLA:HLA00693 DRB1*04:07:01	HLA:HLA00802 DRB1*13:05:01	23	11
HLA:HLA00694 DRB1*04:08:01	HLA:HLA00802 DRB1*13:05:01	23	10
HLA:HLA00685 DRB1*04:01:01	HLA:HLA00810 DRB1*13:12:01	23	10
HLA:HLA00687 DRB1*04:02	HLA:HLA00810 DRB1*13:12:01	23	9
HLA:HLA00693 DRB1*04:07:01	HLA:HLA00810 DRB1*13:12:01	23	10
HLA:HLA00694 DRB1*04:08:01	HLA:HLA00810 DRB1*13:12:01	23	9
HLA:HLA00696 DRB1*04:10	HLA:HLA00810 DRB1*13:12:01	23	8
HLA:HLA00697 DRB1*04:11	HLA:HLA00810 DRB1*13:12:01	23	9
HLA:HLA00685 DRB1*04:01:01	HLA:HLA01724 DRB1*13:56	23	10
HLA:HLA00687 DRB1*04:02	HLA:HLA01724 DRB1*13:56	23	11
HLA:HLA00693 DRB1*04:07:01	HLA:HLA01724 DRB1*13:56	23	10
HLA:HLA00694 DRB1*04:08:01	HLA:HLA01724 DRB1*13:56	23	9
HLA:HLA00688 DRB1*04:03:01	HLA:HLA00719 DRB1*07:01:01:01	24	18
HLA:HLA00689 DRB1*04:04:01	HLA:HLA00719 DRB1*07:01:01:01	24	19
HLA:HLA00692 DRB1*04:06:01	HLA:HLA00719 DRB1*07:01:01:01	24	18
HLA:HLA00688 DRB1*04:03:01	HLA:HLA03486 DRB1*07:01:01:02	24	18
HLA:HLA00689 DRB1*04:04:01	HLA:HLA03486 DRB1*07:01:01:02	24	19
HLA:HLA00692 DRB1*04:06:01	HLA:HLA03486 DRB1*07:01:01:02	24	18
HLA:HLA00688 DRB1*04:03:01	HLA:HLA02017 DRB1*07:09	24	17
HLA:HLA00689 DRB1*04:04:01	HLA:HLA02017 DRB1*07:09	24	18
HLA:HLA00692 DRB1*04:06:01	HLA:HLA02017 DRB1*07:09	24	17
HLA:HLA00724 DRB1*08:02:01	HLA:HLA00750 DRB1*10:01:01	24	15
HLA:HLA00739 DRB1*08:13	HLA:HLA00750 DRB1*10:01:01	24	14
HLA:HLA00750 DRB1*10:01:01	HLA:HLA00751 DRB1*11:01:01	24	13
HLA:HLA00750 DRB1*10:01:01	HLA:HLA00769 DRB1*11:15	24	15
HLA:HLA00750 DRB1*10:01:01	HLA:HLA00797 DRB1*13:01:01	24	16
HLA:HLA00696 DRB1*04:10	HLA:HLA00865 DRB1*15:01:01:01	24	14
HLA:HLA00697 DRB1*04:11	HLA:HLA00865 DRB1*15:01:01:01	24	15
HLA:HLA00797 DRB1*13:01:01	HLA:HLA00865 DRB1*15:01:01:01	24	12
HLA:HLA00696 DRB1*04:10	HLA:HLA03453 DRB1*15:01:01:02	24	14
HLA:HLA00697 DRB1*04:11	HLA:HLA03453 DRB1*15:01:01:02	24	15
HLA:HLA00797 DRB1*13:01:01	HLA:HLA03453 DRB1*15:01:01:02	24	12
HLA:HLA00690 DRB1*04:05:01	HLA:HLA00867 DRB1*15:02:01	24	14
HLA:HLA00695 DRB1*04:09	HLA:HLA00867 DRB1*15:02:01	24	14
HLA:HLA00798 DRB1*13:02:01	HLA:HLA00867 DRB1*15:02:01	24	12
HLA:HLA00802 DRB1*13:05:01	HLA:HLA00867 DRB1*15:02:01	24	14
HLA:HLA00810 DRB1*13:12:01	HLA:HLA00867 DRB1*15:02:01	24	14
HLA:HLA01724 DRB1*13:56	HLA:HLA00867 DRB1*15:02:01	24	14
HLA:HLA00696 DRB1*04:10	HLA:HLA00870 DRB1*15:03:01:01	24	15
HLA:HLA00697 DRB1*04:11	HLA:HLA00870 DRB1*15:03:01:01	24	16
HLA:HLA00797 DRB1*13:01:01	HLA:HLA00870 DRB1*15:03:01:01	24	13
HLA:HLA00696 DRB1*04:10	HLA:HLA03454 DRB1*15:03:01:02	24	15
HLA:HLA00697 DRB1*04:11	HLA:HLA03454 DRB1*15:03:01:02	24	16
HLA:HLA00797 DRB1*13:01:01	HLA:HLA03454 DRB1*15:03:01:02	24	13
HLA:HLA00696 DRB1*04:10	HLA:HLA00871 DRB1*15:04	24	14
HLA:HLA00697 DRB1*04:11	HLA:HLA00871 DRB1*15:04	24	15
HLA:HLA00797 DRB1*13:01:01	HLA:HLA00871 DRB1*15:04	24	13
HLA:HLA02157 DRB1*11:11:02	HLA:HLA00876 DRB1*16:01:01	24	10
HLA:HLA00797 DRB1*13:01:01	HLA:HLA00876 DRB1*16:01:01	24	14
HLA:HLA02157 DRB1*11:11:02	HLA:HLA00878 DRB1*16:02:01	24	11
HLA:HLA00797 DRB1*13:01:01	HLA:HLA00878 DRB1*16:02:01	24	14
HLA:HLA00690 DRB1*04:05:01	HLA:HLA02257 DRB1*08:01:03	24	9
HLA:HLA00695 DRB1*04:09	HLA:HLA02257 DRB1*08:01:03	24	10
HLA:HLA00685 DRB1*04:01:01	HLA:HLA00724 DRB1*08:02:01	24	10
HLA:HLA00687 DRB1*04:02	HLA:HLA00724 DRB1*08:02:01	24	10
HLA:HLA00693 DRB1*04:07:01	HLA:HLA00724 DRB1*08:02:01	24	9
HLA:HLA00694 DRB1*04:08:01	HLA:HLA00724 DRB1*08:02:01	24	9
HLA:HLA00690 DRB1*04:05:01	HLA:HLA00727 DRB1*08:03:02	24	9
HLA:HLA00695 DRB1*04:09	HLA:HLA00727 DRB1*08:03:02	24	10
HLA:HLA00685 DRB1*04:01:01	HLA:HLA00739 DRB1*08:13	24	9
HLA:HLA00687 DRB1*04:02	HLA:HLA00739 DRB1*08:13	24	10
HLA:HLA00693 DRB1*04:07:01	HLA:HLA00739 DRB1*08:13	24	8
HLA:HLA00694 DRB1*04:08:01	HLA:HLA00739 DRB1*08:13	24	8
HLA:HLA00690 DRB1*04:05:01	HLA:HLA00744 DRB1*08:18	24	7
HLA:HLA00695 DRB1*04:09	HLA:HLA00744 DRB1*08:18	24	8
HLA:HLA00665 DRB1*01:02:01	HLA:HLA00750 DRB1*10:01:01	24	10
HLA:HLA00685 DRB1*04:01:01	HLA:HLA00750 DRB1*10:01:01	24	9
HLA:HLA00693 DRB1*04:07:01	HLA:HLA00750 DRB1*10:01:01	24	9
HLA:HLA00694 DRB1*04:08:01	HLA:HLA00750 DRB1*10:01:01	24	8
HLA:HLA00685 DRB1*04:01:01	HLA:HLA00751 DRB1*11:01:01	24	9
HLA:HLA00687 DRB1*04:02	HLA:HLA00751 DRB1*11:01:01	24	9
HLA:HLA00693 DRB1*04:07:01	HLA:HLA00751 DRB1*11:01:01	24	9
HLA:HLA00694 DRB1*04:08:01	HLA:HLA00751 DRB1*11:01:01	24	8
HLA:HLA00688 DRB1*04:03:01	HLA:HLA00755 DRB1*11:03	24	11
HLA:HLA00689 DRB1*04:04:01	HLA:HLA00755 DRB1*11:03	24	10
HLA:HLA00692 DRB1*04:06:01	HLA:HLA00755 DRB1*11:03	24	12
HLA:HLA00688 DRB1*04:03:01	HLA:HLA00756 DRB1*11:04:01	24	9
HLA:HLA00689 DRB1*04:04:01	HLA:HLA00756 DRB1*11:04:01	24	8
HLA:HLA00692 DRB1*04:06:01	HLA:HLA00756 DRB1*11:04:01	24	10
HLA:HLA00688 DRB1*04:03:01	HLA:HLA02157 DRB1*11:11:02	24	12
HLA:HLA00689 DRB1*04:04:01	HLA:HLA02157 DRB1*11:11:02	24	11
HLA:HLA00690 DRB1*04:05:01	HLA:HLA02157 DRB1*11:11:02	24	12
HLA:HLA00692 DRB1*04:06:01	HLA:HLA02157 DRB1*11:11:02	24	13
HLA:HLA00695 DRB1*04:09	HLA:HLA02157 DRB1*11:11:02	24	11
HLA:HLA00685 DRB1*04:01:01	HLA:HLA00769 DRB1*11:15	24	11
HLA:HLA00687 DRB1*04:02	HLA:HLA00769 DRB1*11:15	24	11
HLA:HLA00693 DRB1*04:07:01	HLA:HLA00769 DRB1*11:15	24	11
HLA:HLA00694 DRB1*04:08:01	HLA:HLA00769 DRB1*11:15	24	10
HLA:HLA00685 DRB1*04:01:01	HLA:HLA00797 DRB1*13:01:01	24	12
HLA:HLA02172 DRB1*04:06:02	HLA:HLA00797 DRB1*13:01:01	24	14
HLA:HLA00693 DRB1*04:07:01	HLA:HLA00797 DRB1*13:01:01	24	14

Allele 1	Allele 2	m	K_B	Allele 1	Allele 2	m	K_B
HLA:HLA00694 DRB1*04:08:01	HLA:HLA00797 DRB1*13:01:01	24	13	HLA:HLA00802 DRB1*13:05:01	HLA:HLA00871 DRB1*15:04	25	14
HLA:HLA00696 DRB1*04:10	HLA:HLA00797 DRB1*13:01:01	24	14	HLA:HLA00810 DRB1*13:12:01	HLA:HLA00871 DRB1*15:04	25	16
HLA:HLA00697 DRB1*04:11	HLA:HLA00797 DRB1*13:01:01	24	15	HLA:HLA01724 DRB1*13:56	HLA:HLA00871 DRB1*15:04	25	15
HLA:HLA00688 DRB1*04:03:01	HLA:HLA00798 DRB1*13:02:01	24	14	HLA:HLA00837 DRB1*14:05:01	HLA:HLA00871 DRB1*15:04	25	14
HLA:HLA00689 DRB1*04:04:01	HLA:HLA00798 DRB1*13:02:01	24	13	HLA:HLA02371 DRB1*14:54	HLA:HLA00871 DRB1*15:04	25	16
HLA:HLA00690 DRB1*04:05:01	HLA:HLA00798 DRB1*13:02:01	24	14	HLA:HLA00690 DRB1*04:05:01	HLA:HLA00876 DRB1*16:01:01	25	13
HLA:HLA00692 DRB1*04:06:01	HLA:HLA00798 DRB1*13:02:01	24	15	HLA:HLA00695 DRB1*04:09	HLA:HLA00876 DRB1*16:01:01	25	14
HLA:HLA00695 DRB1*04:09	HLA:HLA00798 DRB1*13:02:01	24	13	HLA:HLA02257 DRB1*08:01:03	HLA:HLA00876 DRB1*16:01:01	25	10
HLA:HLA00688 DRB1*04:03:01	HLA:HLA00802 DRB1*13:05:01	24	12	HLA:HLA00727 DRB1*08:03:02	HLA:HLA00876 DRB1*16:01:01	25	11
HLA:HLA00689 DRB1*04:04:01	HLA:HLA00802 DRB1*13:05:01	24	11	HLA:HLA00735 DRB1*08:09	HLA:HLA00876 DRB1*16:01:01	25	9
HLA:HLA00690 DRB1*04:05:01	HLA:HLA00802 DRB1*13:05:01	24	12	HLA:HLA00744 DRB1*08:18	HLA:HLA00876 DRB1*16:01:01	25	9
HLA:HLA00692 DRB1*04:06:01	HLA:HLA00802 DRB1*13:05:01	24	13	HLA:HLA00751 DRB1*11:01:01	HLA:HLA00876 DRB1*16:01:01	25	8
HLA:HLA00695 DRB1*04:09	HLA:HLA00802 DRB1*13:05:01	24	13	HLA:HLA00769 DRB1*11:15	HLA:HLA00876 DRB1*16:01:01	25	10
HLA:HLA00688 DRB1*04:03:01	HLA:HLA00810 DRB1*13:12:01	24	11	HLA:HLA00690 DRB1*04:05:01	HLA:HLA00878 DRB1*16:02:01	25	12
HLA:HLA00689 DRB1*04:04:01	HLA:HLA00810 DRB1*13:12:01	24	10	HLA:HLA00695 DRB1*04:09	HLA:HLA00878 DRB1*16:02:01	25	13
HLA:HLA00692 DRB1*04:06:01	HLA:HLA00810 DRB1*13:12:01	24	12	HLA:HLA02257 DRB1*08:01:03	HLA:HLA00878 DRB1*16:02:01	25	11
HLA:HLA00687 DRB1*04:02	HLA:HLA00825 DRB1*13:27	24	7	HLA:HLA00727 DRB1*08:03:02	HLA:HLA00878 DRB1*16:02:01	25	11
HLA:HLA00688 DRB1*04:03:01	HLA:HLA01724 DRB1*13:56	24	11	HLA:HLA00735 DRB1*08:09	HLA:HLA00878 DRB1*16:02:01	25	10
HLA:HLA00689 DRB1*04:04:01	HLA:HLA01724 DRB1*13:56	24	10	HLA:HLA00744 DRB1*08:18	HLA:HLA00878 DRB1*16:02:01	25	9
HLA:HLA00690 DRB1*04:05:01	HLA:HLA01724 DRB1*13:56	24	11	HLA:HLA00751 DRB1*11:01:01	HLA:HLA00878 DRB1*16:02:01	25	9
HLA:HLA00692 DRB1*04:06:01	HLA:HLA01724 DRB1*13:56	24	12	HLA:HLA00769 DRB1*11:15	HLA:HLA00878 DRB1*16:02:01	25	11
HLA:HLA00695 DRB1*04:09	HLA:HLA01724 DRB1*13:56	24	12	HLA:HLA00685 DRB1*04:01:01	HLA:HLA02257 DRB1*08:01:03	25	12
HLA:HLA00750 DRB1*10:01:01	HLA:HLA00839 DRB1*14:07:01	24	11	HLA:HLA00687 DRB1*04:02	HLA:HLA02257 DRB1*08:01:03	25	12
HLA:HLA00687 DRB1*04:02	HLA:HLA00719 DRB1*07:01:01:01	25	18	HLA:HLA00693 DRB1*04:07:01	HLA:HLA02257 DRB1*08:01:03	25	11
HLA:HLA02172 DRB1*04:06:02	HLA:HLA00719 DRB1*07:01:01:01	25	18	HLA:HLA00694 DRB1*04:08:01	HLA:HLA02257 DRB1*08:01:03	25	11
HLA:HLA00687 DRB1*04:02	HLA:HLA03486 DRB1*07:01:01:02	25	18	HLA:HLA00696 DRB1*04:10	HLA:HLA02257 DRB1*08:01:03	25	10
HLA:HLA02172 DRB1*04:06:02	HLA:HLA03486 DRB1*07:01:01:02	25	18	HLA:HLA00697 DRB1*04:11	HLA:HLA02257 DRB1*08:01:03	25	10
HLA:HLA00687 DRB1*04:02	HLA:HLA02017 DRB1*07:09	25	17	HLA:HLA00688 DRB1*04:03:01	HLA:HLA00724 DRB1*08:02:01	25	10
HLA:HLA02172 DRB1*04:06:02	HLA:HLA02017 DRB1*07:09	25	17	HLA:HLA00689 DRB1*04:04:01	HLA:HLA00724 DRB1*08:02:01	25	10
HLA:HLA02257 DRB1*08:01:03	HLA:HLA00750 DRB1*10:01:01	25	17	HLA:HLA00690 DRB1*04:05:01	HLA:HLA00724 DRB1*08:02:01	25	11
HLA:HLA00727 DRB1*08:03:02	HLA:HLA00750 DRB1*10:01:01	25	17	HLA:HLA00692 DRB1*04:06:01	HLA:HLA00724 DRB1*08:02:01	25	11
HLA:HLA00735 DRB1*08:09	HLA:HLA00750 DRB1*10:01:01	25	15	HLA:HLA00695 DRB1*04:09	HLA:HLA00724 DRB1*08:02:01	25	12
HLA:HLA00744 DRB1*08:18	HLA:HLA00750 DRB1*10:01:01	25	15	HLA:HLA00685 DRB1*04:01:01	HLA:HLA00727 DRB1*08:03:02	25	12
HLA:HLA00750 DRB1*10:01:01	HLA:HLA00752 DRB1*11:01:02	25	13	HLA:HLA00687 DRB1*04:02	HLA:HLA00727 DRB1*08:03:02	25	11
HLA:HLA00750 DRB1*10:01:01	HLA:HLA00755 DRB1*11:03	25	16	HLA:HLA00693 DRB1*04:07:01	HLA:HLA00727 DRB1*08:03:02	25	11
HLA:HLA00750 DRB1*10:01:01	HLA:HLA00756 DRB1*11:04:01	25	14	HLA:HLA00694 DRB1*04:08:01	HLA:HLA00727 DRB1*08:03:02	25	11
HLA:HLA00750 DRB1*10:01:01	HLA:HLA02157 DRB1*11:11:02	25	15	HLA:HLA00696 DRB1*04:10	HLA:HLA00727 DRB1*08:03:02	25	10
HLA:HLA00688 DRB1*04:03:01	HLA:HLA00865 DRB1*15:01:01:01	25	13	HLA:HLA00697 DRB1*04:11	HLA:HLA00727 DRB1*08:03:02	25	10
HLA:HLA00689 DRB1*04:04:01	HLA:HLA00865 DRB1*15:01:01:01	25	12	HLA:HLA00685 DRB1*04:01:01	HLA:HLA00735 DRB1*08:09	25	12
HLA:HLA00690 DRB1*04:05:01	HLA:HLA00865 DRB1*15:01:01:01	25	15	HLA:HLA00687 DRB1*04:02	HLA:HLA00735 DRB1*08:09	25	12
HLA:HLA00692 DRB1*04:06:01	HLA:HLA00865 DRB1*15:01:01:01	25	12	HLA:HLA00693 DRB1*04:07:01	HLA:HLA00735 DRB1*08:09	25	11
HLA:HLA00695 DRB1*04:09	HLA:HLA00865 DRB1*15:01:01:01	25	15	HLA:HLA00694 DRB1*04:08:01	HLA:HLA00735 DRB1*08:09	25	11
HLA:HLA00798 DRB1*13:02:01	HLA:HLA00865 DRB1*15:01:01:01	25	13	HLA:HLA00688 DRB1*04:03:01	HLA:HLA00739 DRB1*08:13	25	9
HLA:HLA00802 DRB1*13:05:01	HLA:HLA00865 DRB1*15:01:01:01	25	15	HLA:HLA00689 DRB1*04:04:01	HLA:HLA00739 DRB1*08:13	25	9
HLA:HLA00810 DRB1*13:12:01	HLA:HLA00865 DRB1*15:01:01:01	25	15	HLA:HLA00690 DRB1*04:05:01	HLA:HLA00739 DRB1*08:13	25	10
HLA:HLA01724 DRB1*13:56	HLA:HLA00865 DRB1*15:01:01:01	25	15	HLA:HLA00692 DRB1*04:06:01	HLA:HLA00739 DRB1*08:13	25	10
HLA:HLA00837 DRB1*14:05:01	HLA:HLA00865 DRB1*15:01:01:01	25	14	HLA:HLA00695 DRB1*04:09	HLA:HLA00739 DRB1*08:13	25	11
HLA:HLA02371 DRB1*14:54	HLA:HLA00865 DRB1*15:01:01:01	25	16	HLA:HLA00685 DRB1*04:01:01	HLA:HLA00744 DRB1*08:18	25	10
HLA:HLA00688 DRB1*04:03:01	HLA:HLA03453 DRB1*15:01:01:02	25	13	HLA:HLA00687 DRB1*04:02	HLA:HLA00744 DRB1*08:18	25	9
HLA:HLA00689 DRB1*04:04:01	HLA:HLA03453 DRB1*15:01:01:02	25	12	HLA:HLA00693 DRB1*04:07:01	HLA:HLA00744 DRB1*08:18	25	10
HLA:HLA00690 DRB1*04:05:01	HLA:HLA03453 DRB1*15:01:01:02	25	15	HLA:HLA00694 DRB1*04:08:01	HLA:HLA00744 DRB1*08:18	25	9
HLA:HLA00692 DRB1*04:06:01	HLA:HLA03453 DRB1*15:01:01:02	25	12	HLA:HLA00696 DRB1*04:10	HLA:HLA00744 DRB1*08:18	25	8
HLA:HLA00695 DRB1*04:09	HLA:HLA03453 DRB1*15:01:01:02	25	15	HLA:HLA00697 DRB1*04:11	HLA:HLA00744 DRB1*08:18	25	9
HLA:HLA00798 DRB1*13:02:01	HLA:HLA03453 DRB1*15:01:01:02	25	13	HLA:HLA00688 DRB1*04:03:01	HLA:HLA00750 DRB1*10:01:01	25	10
HLA:HLA00802 DRB1*13:05:01	HLA:HLA03453 DRB1*15:01:01:02	25	15	HLA:HLA00689 DRB1*04:04:01	HLA:HLA00750 DRB1*10:01:01	25	9
HLA:HLA00810 DRB1*13:12:01	HLA:HLA03453 DRB1*15:01:01:02	25	15	HLA:HLA00690 DRB1*04:05:01	HLA:HLA00750 DRB1*10:01:01	25	10
HLA:HLA01724 DRB1*13:56	HLA:HLA03453 DRB1*15:01:01:02	25	15	HLA:HLA00692 DRB1*04:06:01	HLA:HLA00750 DRB1*10:01:01	25	11
HLA:HLA00837 DRB1*14:05:01	HLA:HLA03453 DRB1*15:01:01:02	25	14	HLA:HLA00695 DRB1*04:09	HLA:HLA00750 DRB1*10:01:01	25	11
HLA:HLA02371 DRB1*14:54	HLA:HLA03453 DRB1*15:01:01:02	25	16	HLA:HLA00688 DRB1*04:03:01	HLA:HLA00751 DRB1*11:01:01	25	10
HLA:HLA00685 DRB1*04:01:01	HLA:HLA00867 DRB1*15:02:01	25	12	HLA:HLA00689 DRB1*04:04:01	HLA:HLA00751 DRB1*11:01:01	25	9
HLA:HLA00693 DRB1*04:07:01	HLA:HLA00867 DRB1*15:02:01	25	13	HLA:HLA00690 DRB1*04:05:01	HLA:HLA00751 DRB1*11:01:01	25	10
HLA:HLA00694 DRB1*04:08:01	HLA:HLA00867 DRB1*15:02:01	25	12	HLA:HLA00692 DRB1*04:06:01	HLA:HLA00751 DRB1*11:01:01	25	11
HLA:HLA00696 DRB1*04:10	HLA:HLA00867 DRB1*15:02:01	25	15	HLA:HLA00695 DRB1*04:09	HLA:HLA00751 DRB1*11:01:01	25	11
HLA:HLA00697 DRB1*04:11	HLA:HLA00867 DRB1*15:02:01	25	16	HLA:HLA00685 DRB1*04:01:01	HLA:HLA00752 DRB1*11:01:02	25	9
HLA:HLA00719 DRB1*07:01:01:01	HLA:HLA00867 DRB1*15:02:01	25	21	HLA:HLA00687 DRB1*04:02	HLA:HLA00752 DRB1*11:01:02	25	9
HLA:HLA03486 DRB1*07:01:01:02	HLA:HLA00867 DRB1*15:02:01	25	21	HLA:HLA00693 DRB1*04:07:01	HLA:HLA00752 DRB1*11:01:02	25	9
HLA:HLA02017 DRB1*07:09	HLA:HLA00867 DRB1*15:02:01	25	20	HLA:HLA00694 DRB1*04:08:01	HLA:HLA00752 DRB1*11:01:02	25	8
HLA:HLA02157 DRB1*11:11:02	HLA:HLA00867 DRB1*15:02:01	25	11	HLA:HLA00685 DRB1*04:01:01	HLA:HLA00755 DRB1*11:03	25	10
HLA:HLA00797 DRB1*13:01:01	HLA:HLA00867 DRB1*15:02:01	25	13	HLA:HLA02172 DRB1*04:06:02	HLA:HLA00755 DRB1*11:03	25	12
HLA:HLA00839 DRB1*14:07:01	HLA:HLA00867 DRB1*15:02:01	25	16	HLA:HLA00693 DRB1*04:07:01	HLA:HLA00755 DRB1*11:03	25	12
HLA:HLA00688 DRB1*04:03:01	HLA:HLA00870 DRB1*15:03:01:01	25	14	HLA:HLA00694 DRB1*04:08:01	HLA:HLA00755 DRB1*11:03	25	11
HLA:HLA00689 DRB1*04:04:01	HLA:HLA00870 DRB1*15:03:01:01	25	13	HLA:HLA00696 DRB1*04:10	HLA:HLA00755 DRB1*11:03	25	12
HLA:HLA00690 DRB1*04:05:01	HLA:HLA00870 DRB1*15:03:01:01	25	16	HLA:HLA00697 DRB1*04:11	HLA:HLA00755 DRB1*11:03	25	13
HLA:HLA00692 DRB1*04:06:01	HLA:HLA00870 DRB1*15:03:01:01	25	13	HLA:HLA00685 DRB1*04:01:01	HLA:HLA00756 DRB1*11:04:01	25	10
HLA:HLA00695 DRB1*04:09	HLA:HLA00870 DRB1*15:03:01:01	25	16	HLA:HLA02172 DRB1*04:06:02	HLA:HLA00756 DRB1*11:04:01	25	10
HLA:HLA00798 DRB1*13:02:01	HLA:HLA00870 DRB1*15:03:01:01	25	14	HLA:HLA00693 DRB1*04:07:01	HLA:HLA00756 DRB1*11:04:01	25	10
HLA:HLA00802 DRB1*13:05:01	HLA:HLA00870 DRB1*15:03:01:01	25	16	HLA:HLA00694 DRB1*04:08:01	HLA:HLA00756 DRB1*11:04:01	25	9
HLA:HLA00810 DRB1*13:12:01	HLA:HLA00870 DRB1*15:03:01:01	25	16	HLA:HLA00696 DRB1*04:10	HLA:HLA00756 DRB1*11:04:01	25	10
HLA:HLA01724 DRB1*13:56	HLA:HLA00870 DRB1*15:03:01:01	25	16	HLA:HLA00697 DRB1*04:11	HLA:HLA00756 DRB1*11:04:01	25	11
HLA:HLA00837 DRB1*14:05:01	HLA:HLA00870 DRB1*15:03:01:01	25	15	HLA:HLA02172 DRB1*04:06:02	HLA:HLA02157 DRB1*11:11:02	25	13
HLA:HLA02371 DRB1*14:54	HLA:HLA00870 DRB1*15:03:01:01	25	17	HLA:HLA00696 DRB1*04:10	HLA:HLA02157 DRB1*11:11:02	25	13
HLA:HLA00688 DRB1*04:03:01	HLA:HLA03454 DRB1*15:03:01:02	25	14	HLA:HLA00697 DRB1*04:11	HLA:HLA02157 DRB1*11:11:02	25	14
HLA:HLA00689 DRB1*04:04:01	HLA:HLA03454 DRB1*15:03:01:02	25	13	HLA:HLA00688 DRB1*04:03:01	HLA:HLA00769 DRB1*11:15	25	12
HLA:HLA00690 DRB1*04:05:01	HLA:HLA03454 DRB1*15:03:01:02	25	16	HLA:HLA00689 DRB1*04:04:01	HLA:HLA00769 DRB1*11:15	25	11
HLA:HLA00692 DRB1*04:06:01	HLA:HLA03454 DRB1*15:03:01:02	25	13	HLA:HLA00690 DRB1*04:05:01	HLA:HLA00769 DRB1*11:15	25	12
HLA:HLA00695 DRB1*04:09	HLA:HLA03454 DRB1*15:03:01:02	25	16	HLA:HLA00692 DRB1*04:06:01	HLA:HLA00769 DRB1*11:15	25	13
HLA:HLA00798 DRB1*13:02:01	HLA:HLA03454 DRB1*15:03:01:02	25	14	HLA:HLA00695 DRB1*04:09	HLA:HLA00769 DRB1*11:15	25	13
HLA:HLA00802 DRB1*13:05:01	HLA:HLA03454 DRB1*15:03:01:02	25	16	HLA:HLA00690 DRB1*04:05:01	HLA:HLA00797 DRB1*13:01:01	25	15
HLA:HLA00810 DRB1*13:12:01	HLA:HLA03454 DRB1*15:03:01:02	25	16	HLA:HLA00695 DRB1*04:09	HLA:HLA00797 DRB1*13:01:01	25	14
HLA:HLA01724 DRB1*13:56	HLA:HLA03454 DRB1*15:03:01:02	25	16	HLA:HLA02172 DRB1*04:06:02	HLA:HLA00798 DRB1*13:02:01	25	15
HLA:HLA00837 DRB1*14:05:01	HLA:HLA03454 DRB1*15:03:01:02	25	15	HLA:HLA00696 DRB1*04:10	HLA:HLA00798 DRB1*13:02:01	25	15
HLA:HLA02371 DRB1*14:54	HLA:HLA03454 DRB1*15:03:01:02	25	17	HLA:HLA00697 DRB1*04:11	HLA:HLA00798 DRB1*13:02:01	25	16
HLA:HLA00688 DRB1*04:03:01	HLA:HLA00871 DRB1*15:04	25	13	HLA:HLA02172 DRB1*04:06:02	HLA:HLA00802 DRB1*13:05:01	25	13
HLA:HLA00689 DRB1*04:04:01	HLA:HLA00871 DRB1*15:04	25	12	HLA:HLA00696 DRB1*04:10	HLA:HLA00802 DRB1*13:05:01	25	13
HLA:HLA00690 DRB1*04:05:01	HLA:HLA00871 DRB1*15:04	25	15	HLA:HLA00697 DRB1*04:11	HLA:HLA00802 DRB1*13:05:01	25	14
HLA:HLA00692 DRB1*04:06:01	HLA:HLA00871 DRB1*15:04	25	12	HLA:HLA02172 DRB1*04:06:02	HLA:HLA00810 DRB1*13:12:01	25	12
HLA:HLA00695 DRB1*04:09	HLA:HLA00871 DRB1*15:04	25	15	HLA:HLA00688 DRB1*04:03:01	HLA:HLA00825 DRB1*13:27	25	13
HLA:HLA00798 DRB1*13:02:01	HLA:HLA00871 DRB1*15:04	25	14	HLA:HLA00689 DRB1*04:04:01	HLA:HLA00825 DRB1*13:27	25	12

Allele 1	Allele 2	m	K_B
HLA:HLA00692 DRB1*04:06:01	HLA:HLA00825 DRB1*13:27	25	14
HLA:HLA02172 DRB1*04:06:02	HLA:HLA01724 DRB1*13:56	25	12
HLA:HLA00696 DRB1*04:10	HLA:HLA01724 DRB1*13:56	25	12
HLA:HLA00697 DRB1*04:11	HLA:HLA01724 DRB1*13:56	25	13
HLA:HLA00685 DRB1*04:01:01	HLA:HLA00839 DRB1*14:07:01	25	11
HLA:HLA00693 DRB1*04:07:01	HLA:HLA00839 DRB1*14:07:01	25	9
HLA:HLA00694 DRB1*04:08:01	HLA:HLA00839 DRB1*14:07:01	25	10
HLA:HLA00665 DRB1*01:02:01	HLA:HLA01693 DRB1*14:46	25	10
HLA:HLA00688 DRB1*04:03:01	HLA:HLA02371 DRB1*14:54	25	9
HLA:HLA00689 DRB1*04:04:01	HLA:HLA02371 DRB1*14:54	25	10
HLA:HLA00692 DRB1*04:06:01	HLA:HLA02371 DRB1*14:54	25	9
HLA:HLA00750 DRB1*10:01:01	HLA:HLA02371 DRB1*14:54	25	12
HLA:HLA00749 DRB1*09:01:02	HLA:HLA00750 DRB1*10:01:01	26	15
HLA:HLA00750 DRB1*10:01:01	HLA:HLA01407 DRB1*12:01:02	26	18
HLA:HLA00750 DRB1*10:01:01	HLA:HLA00825 DRB1*13:27	26	16
HLA:HLA00749 DRB1*09:01:02	HLA:HLA01693 DRB1*14:46	26	17
HLA:HLA00685 DRB1*04:01:01	HLA:HLA00865 DRB1*15:01:01:01	26	13
HLA:HLA00687 DRB1*04:02	HLA:HLA00865 DRB1*15:01:01:01	26	12
HLA:HLA02172 DRB1*04:06:02	HLA:HLA00865 DRB1*15:01:01:01	26	12
HLA:HLA00693 DRB1*04:07:01	HLA:HLA00865 DRB1*15:01:01:01	26	14
HLA:HLA00694 DRB1*04:08:01	HLA:HLA00865 DRB1*15:01:01:01	26	13
HLA:HLA00719 DRB1*07:01:01:01	HLA:HLA00865 DRB1*15:01:01:01	26	22
HLA:HLA03486 DRB1*07:01:01:02	HLA:HLA00865 DRB1*15:01:01:01	26	22
HLA:HLA02017 DRB1*07:09	HLA:HLA00865 DRB1*15:01:01:01	26	21
HLA:HLA00755 DRB1*11:03	HLA:HLA00865 DRB1*15:01:01:01	26	11
HLA:HLA00756 DRB1*11:04:01	HLA:HLA00865 DRB1*15:01:01:01	26	12
HLA:HLA02157 DRB1*11:11:02	HLA:HLA00865 DRB1*15:01:01:01	26	12
HLA:HLA01407 DRB1*12:01:02	HLA:HLA00865 DRB1*15:01:01:01	26	18
HLA:HLA00825 DRB1*13:27	HLA:HLA00865 DRB1*15:01:01:01	26	12
HLA:HLA00833 DRB1*14:01:01	HLA:HLA00865 DRB1*15:01:01:01	26	16
HLA:HLA00836 DRB1*14:04	HLA:HLA00865 DRB1*15:01:01:01	26	15
HLA:HLA00839 DRB1*14:07:01	HLA:HLA00865 DRB1*15:01:01:01	26	17
HLA:HLA00685 DRB1*04:01:01	HLA:HLA03453 DRB1*15:01:01:02	26	13
HLA:HLA00687 DRB1*04:02	HLA:HLA03453 DRB1*15:01:01:02	26	12
HLA:HLA02172 DRB1*04:06:02	HLA:HLA03453 DRB1*15:01:01:02	26	12
HLA:HLA00693 DRB1*04:07:01	HLA:HLA03453 DRB1*15:01:01:02	26	14
HLA:HLA00694 DRB1*04:08:01	HLA:HLA03453 DRB1*15:01:01:02	26	13
HLA:HLA00719 DRB1*07:01:01:01	HLA:HLA03453 DRB1*15:01:01:02	26	22
HLA:HLA03486 DRB1*07:01:01:02	HLA:HLA03453 DRB1*15:01:01:02	26	22
HLA:HLA02017 DRB1*07:09	HLA:HLA03453 DRB1*15:01:01:02	26	21
HLA:HLA00755 DRB1*11:03	HLA:HLA03453 DRB1*15:01:01:02	26	11
HLA:HLA00756 DRB1*11:04:01	HLA:HLA03453 DRB1*15:01:01:02	26	12
HLA:HLA02157 DRB1*11:11:02	HLA:HLA03453 DRB1*15:01:01:02	26	12
HLA:HLA01407 DRB1*12:01:02	HLA:HLA03453 DRB1*15:01:01:02	26	18
HLA:HLA00825 DRB1*13:27	HLA:HLA03453 DRB1*15:01:01:02	26	12
HLA:HLA00833 DRB1*14:01:01	HLA:HLA03453 DRB1*15:01:01:02	26	16
HLA:HLA00836 DRB1*14:04	HLA:HLA03453 DRB1*15:01:01:02	26	15
HLA:HLA00839 DRB1*14:07:01	HLA:HLA03453 DRB1*15:01:01:02	26	17
HLA:HLA00688 DRB1*04:03:01	HLA:HLA00867 DRB1*15:02:01	26	14
HLA:HLA00689 DRB1*04:04:01	HLA:HLA00867 DRB1*15:02:01	26	13
HLA:HLA00692 DRB1*04:06:01	HLA:HLA00867 DRB1*15:02:01	26	13
HLA:HLA02257 DRB1*08:01:03	HLA:HLA00867 DRB1*15:02:01	26	16
HLA:HLA00727 DRB1*08:03:02	HLA:HLA00867 DRB1*15:02:01	26	15
HLA:HLA00735 DRB1*08:09	HLA:HLA00867 DRB1*15:02:01	26	15
HLA:HLA00744 DRB1*08:18	HLA:HLA00867 DRB1*15:02:01	26	13
HLA:HLA00749 DRB1*09:01:02	HLA:HLA00867 DRB1*15:02:01	26	23
HLA:HLA00751 DRB1*11:01:01	HLA:HLA00867 DRB1*15:02:01	26	12
HLA:HLA00769 DRB1*11:15	HLA:HLA00867 DRB1*15:02:01	26	14
HLA:HLA00837 DRB1*14:05:01	HLA:HLA00867 DRB1*15:02:01	26	15
HLA:HLA02371 DRB1*14:54	HLA:HLA00867 DRB1*15:02:01	26	17
HLA:HLA00685 DRB1*04:01:01	HLA:HLA00870 DRB1*15:03:01:01	26	14
HLA:HLA00687 DRB1*04:02	HLA:HLA00870 DRB1*15:03:01:01	26	13
HLA:HLA02172 DRB1*04:06:02	HLA:HLA00870 DRB1*15:03:01:01	26	13
HLA:HLA00693 DRB1*04:07:01	HLA:HLA00870 DRB1*15:03:01:01	26	15
HLA:HLA00694 DRB1*04:08:01	HLA:HLA00870 DRB1*15:03:01:01	26	14
HLA:HLA00719 DRB1*07:01:01:01	HLA:HLA00870 DRB1*15:03:01:01	26	21
HLA:HLA03486 DRB1*07:01:01:02	HLA:HLA00870 DRB1*15:03:01:01	26	21
HLA:HLA02017 DRB1*07:09	HLA:HLA00870 DRB1*15:03:01:01	26	22
HLA:HLA00755 DRB1*11:03	HLA:HLA00870 DRB1*15:03:01:01	26	12
HLA:HLA00756 DRB1*11:04:01	HLA:HLA00870 DRB1*15:03:01:01	26	13
HLA:HLA02157 DRB1*11:11:02	HLA:HLA00870 DRB1*15:03:01:01	26	13
HLA:HLA01407 DRB1*12:01:02	HLA:HLA00870 DRB1*15:03:01:01	26	17
HLA:HLA00825 DRB1*13:27	HLA:HLA00870 DRB1*15:03:01:01	26	13
HLA:HLA00833 DRB1*14:01:01	HLA:HLA00870 DRB1*15:03:01:01	26	17
HLA:HLA00836 DRB1*14:04	HLA:HLA00870 DRB1*15:03:01:01	26	16
HLA:HLA00839 DRB1*14:07:01	HLA:HLA00870 DRB1*15:03:01:01	26	18
HLA:HLA00685 DRB1*04:01:01	HLA:HLA03454 DRB1*15:03:01:02	26	14
HLA:HLA00687 DRB1*04:02	HLA:HLA03454 DRB1*15:03:01:02	26	13
HLA:HLA02172 DRB1*04:06:02	HLA:HLA03454 DRB1*15:03:01:02	26	13
HLA:HLA00693 DRB1*04:07:01	HLA:HLA03454 DRB1*15:03:01:02	26	15
HLA:HLA00694 DRB1*04:08:01	HLA:HLA03454 DRB1*15:03:01:02	26	14
HLA:HLA00719 DRB1*07:01:01:01	HLA:HLA03454 DRB1*15:03:01:02	26	21
HLA:HLA03486 DRB1*07:01:01:02	HLA:HLA03454 DRB1*15:03:01:02	26	21
HLA:HLA02017 DRB1*07:09	HLA:HLA03454 DRB1*15:03:01:02	26	22
HLA:HLA00755 DRB1*11:03	HLA:HLA03454 DRB1*15:03:01:02	26	12
HLA:HLA00756 DRB1*11:04:01	HLA:HLA03454 DRB1*15:03:01:02	26	13
HLA:HLA02157 DRB1*11:11:02	HLA:HLA03454 DRB1*15:03:01:02	26	13
HLA:HLA01407 DRB1*12:01:02	HLA:HLA03454 DRB1*15:03:01:02	26	17
HLA:HLA00825 DRB1*13:27	HLA:HLA03454 DRB1*15:03:01:02	26	13
HLA:HLA00833 DRB1*14:01:01	HLA:HLA03454 DRB1*15:03:01:02	26	17
HLA:HLA00836 DRB1*14:04	HLA:HLA03454 DRB1*15:03:01:02	26	16
HLA:HLA00839 DRB1*14:07:01	HLA:HLA03454 DRB1*15:03:01:02	26	18
HLA:HLA00685 DRB1*04:01:01	HLA:HLA00871 DRB1*15:04	26	13
HLA:HLA00687 DRB1*04:02	HLA:HLA00871 DRB1*15:04	26	13
HLA:HLA02172 DRB1*04:06:02	HLA:HLA00871 DRB1*15:04	26	12
HLA:HLA00693 DRB1*04:07:01	HLA:HLA00871 DRB1*15:04	26	14
HLA:HLA00694 DRB1*04:08:01	HLA:HLA00871 DRB1*15:04	26	13
HLA:HLA00719 DRB1*07:01:01:01	HLA:HLA00871 DRB1*15:04	26	23
HLA:HLA03486 DRB1*07:01:01:02	HLA:HLA00871 DRB1*15:04	26	23
HLA:HLA02017 DRB1*07:09	HLA:HLA00871 DRB1*15:04	26	22
HLA:HLA00755 DRB1*11:03	HLA:HLA00871 DRB1*15:04	26	10
HLA:HLA00756 DRB1*11:04:01	HLA:HLA00871 DRB1*15:04	26	11
HLA:HLA02157 DRB1*11:11:02	HLA:HLA00871 DRB1*15:04	26	11
HLA:HLA01407 DRB1*12:01:02	HLA:HLA00871 DRB1*15:04	26	19
HLA:HLA00825 DRB1*13:27	HLA:HLA00871 DRB1*15:04	26	13
HLA:HLA00833 DRB1*14:01:01	HLA:HLA00871 DRB1*15:04	26	16
HLA:HLA00836 DRB1*14:04	HLA:HLA00871 DRB1*15:04	26	15
HLA:HLA00839 DRB1*14:07:01	HLA:HLA00871 DRB1*15:04	26	17
HLA:HLA00685 DRB1*04:01:01	HLA:HLA00876 DRB1*16:01:01	26	12
HLA:HLA00687 DRB1*04:02	HLA:HLA00876 DRB1*16:01:01	26	12
HLA:HLA00693 DRB1*04:07:01	HLA:HLA00876 DRB1*16:01:01	26	12
HLA:HLA00694 DRB1*04:08:01	HLA:HLA00876 DRB1*16:01:01	26	11
HLA:HLA00696 DRB1*04:10	HLA:HLA00876 DRB1*16:01:01	26	14
HLA:HLA00697 DRB1*04:11	HLA:HLA00876 DRB1*16:01:01	26	15
HLA:HLA00719 DRB1*07:01:01:01	HLA:HLA00876 DRB1*16:01:01	26	17
HLA:HLA03486 DRB1*07:01:01:02	HLA:HLA00876 DRB1*16:01:01	26	17
HLA:HLA02017 DRB1*07:09	HLA:HLA00876 DRB1*16:01:01	26	16
HLA:HLA00724 DRB1*08:02:01	HLA:HLA00876 DRB1*16:01:01	26	8
HLA:HLA00739 DRB1*08:13	HLA:HLA00876 DRB1*16:01:01	26	9
HLA:HLA00752 DRB1*11:01:02	HLA:HLA00876 DRB1*16:01:01	26	8
HLA:HLA00755 DRB1*11:03	HLA:HLA00876 DRB1*16:01:01	26	11
HLA:HLA00756 DRB1*11:04:01	HLA:HLA00876 DRB1*16:01:01	26	9
HLA:HLA01407 DRB1*12:01:02	HLA:HLA00876 DRB1*16:01:01	26	17
HLA:HLA00825 DRB1*13:27	HLA:HLA00876 DRB1*16:01:01	26	14
HLA:HLA00685 DRB1*04:01:01	HLA:HLA00878 DRB1*16:02:01	26	11
HLA:HLA00687 DRB1*04:02	HLA:HLA00878 DRB1*16:02:01	26	12
HLA:HLA00693 DRB1*04:07:01	HLA:HLA00878 DRB1*16:02:01	26	11
HLA:HLA00694 DRB1*04:08:01	HLA:HLA00878 DRB1*16:02:01	26	10
HLA:HLA00696 DRB1*04:10	HLA:HLA00878 DRB1*16:02:01	26	13
HLA:HLA00697 DRB1*04:11	HLA:HLA00878 DRB1*16:02:01	26	14
HLA:HLA00719 DRB1*07:01:01:01	HLA:HLA00878 DRB1*16:02:01	26	17
HLA:HLA03486 DRB1*07:01:01:02	HLA:HLA00878 DRB1*16:02:01	26	17
HLA:HLA02017 DRB1*07:09	HLA:HLA00878 DRB1*16:02:01	26	16
HLA:HLA00724 DRB1*08:02:01	HLA:HLA00878 DRB1*16:02:01	26	9
HLA:HLA00739 DRB1*08:13	HLA:HLA00878 DRB1*16:02:01	26	8
HLA:HLA00752 DRB1*11:01:02	HLA:HLA00878 DRB1*16:02:01	26	9
HLA:HLA00755 DRB1*11:03	HLA:HLA00878 DRB1*16:02:01	26	12
HLA:HLA00756 DRB1*11:04:01	HLA:HLA00878 DRB1*16:02:01	26	10
HLA:HLA01407 DRB1*12:01:02	HLA:HLA00878 DRB1*16:02:01	26	17
HLA:HLA00825 DRB1*13:27	HLA:HLA00878 DRB1*16:02:01	26	14
HLA:HLA00671 DRB1*03:01:01:01	HLA:HLA00688 DRB1*04:03:01	26	10
HLA:HLA03483 DRB1*03:01:01:02	HLA:HLA00688 DRB1*04:03:01	26	10
HLA:HLA00678 DRB1*03:06	HLA:HLA00688 DRB1*04:03:01	26	9
HLA:HLA00671 DRB1*03:01:01:01	HLA:HLA00689 DRB1*04:04:01	26	10
HLA:HLA03483 DRB1*03:01:01:02	HLA:HLA00689 DRB1*04:04:01	26	10
HLA:HLA00678 DRB1*03:06	HLA:HLA00689 DRB1*04:04:01	26	9
HLA:HLA00671 DRB1*03:01:01:01	HLA:HLA00692 DRB1*04:06:01	26	11
HLA:HLA03483 DRB1*03:01:01:02	HLA:HLA00692 DRB1*04:06:01	26	11
HLA:HLA00678 DRB1*03:06	HLA:HLA00692 DRB1*04:06:01	26	10
HLA:HLA00688 DRB1*04:03:01	HLA:HLA02257 DRB1*08:01:03	26	12
HLA:HLA00689 DRB1*04:04:01	HLA:HLA02257 DRB1*08:01:03	26	12
HLA:HLA00692 DRB1*04:06:01	HLA:HLA02257 DRB1*08:01:03	26	13
HLA:HLA02172 DRB1*04:06:02	HLA:HLA00724 DRB1*08:02:01	26	11
HLA:HLA00696 DRB1*04:10	HLA:HLA00724 DRB1*08:02:01	26	12
HLA:HLA00697 DRB1*04:11	HLA:HLA00724 DRB1*08:02:01	26	12
HLA:HLA00688 DRB1*04:03:01	HLA:HLA00727 DRB1*08:03:02	26	12
HLA:HLA00689 DRB1*04:04:01	HLA:HLA00727 DRB1*08:03:02	26	12
HLA:HLA00692 DRB1*04:06:01	HLA:HLA00727 DRB1*08:03:02	26	13
HLA:HLA00688 DRB1*04:03:01	HLA:HLA00735 DRB1*08:09	26	12
HLA:HLA00689 DRB1*04:04:01	HLA:HLA00735 DRB1*08:09	26	12
HLA:HLA00690 DRB1*04:05:01	HLA:HLA00735 DRB1*08:09	26	13
HLA:HLA00692 DRB1*04:06:01	HLA:HLA00735 DRB1*08:09	26	12
HLA:HLA00695 DRB1*04:09	HLA:HLA00735 DRB1*08:09	26	14
HLA:HLA02172 DRB1*04:06:02	HLA:HLA00739 DRB1*08:13	26	10
HLA:HLA00696 DRB1*04:10	HLA:HLA00739 DRB1*08:13	26	11
HLA:HLA00697 DRB1*04:11	HLA:HLA00739 DRB1*08:13	26	11
HLA:HLA00688 DRB1*04:03:01	HLA:HLA00744 DRB1*08:18	26	11
HLA:HLA00689 DRB1*04:04:01	HLA:HLA00744 DRB1*08:18	26	10
HLA:HLA00692 DRB1*04:06:01	HLA:HLA00744 DRB1*08:18	26	12
HLA:HLA00671 DRB1*03:01:01:01	HLA:HLA00750 DRB1*10:01:01	26	14
HLA:HLA03483 DRB1*03:01:01:02	HLA:HLA00750 DRB1*10:01:01	26	14
HLA:HLA00678 DRB1*03:06	HLA:HLA00750 DRB1*10:01:01	26	13
HLA:HLA02172 DRB1*04:06:02	HLA:HLA00750 DRB1*10:01:01	26	11
HLA:HLA00696 DRB1*04:10	HLA:HLA00750 DRB1*10:01:01	26	11
HLA:HLA00697 DRB1*04:11	HLA:HLA00750 DRB1*10:01:01	26	12
HLA:HLA02172 DRB1*04:06:02	HLA:HLA00751 DRB1*11:01:01	26	11
HLA:HLA00696 DRB1*04:10	HLA:HLA00751 DRB1*11:01:01	26	11
HLA:HLA00697 DRB1*04:11	HLA:HLA00751 DRB1*11:01:01	26	12
HLA:HLA00688 DRB1*04:03:01	HLA:HLA00752 DRB1*11:01:02	26	10
HLA:HLA00689 DRB1*04:04:01	HLA:HLA00752 DRB1*11:01:02	26	9
HLA:HLA00690 DRB1*04:05:01	HLA:HLA00752 DRB1*11:01:02	26	10
HLA:HLA00692 DRB1*04:06:01	HLA:HLA00752 DRB1*11:01:02	26	11
HLA:HLA00695 DRB1*04:09	HLA:HLA00752 DRB1*11:01:02	26	11
HLA:HLA00690 DRB1*04:05:01	HLA:HLA00755 DRB1*11:03	26	13
HLA:HLA00695 DRB1*04:09	HLA:HLA00755 DRB1*11:03	26	12
HLA:HLA00690 DRB1*04:05:01	HLA:HLA00756 DRB1*11:04:01	26	11
HLA:HLA00695 DRB1*04:09	HLA:HLA00756 DRB1*11:04:01	26	12
HLA:HLA02172 DRB1*04:06:02	HLA:HLA00769 DRB1*11:15	26	13
HLA:HLA00696 DRB1*04:10	HLA:HLA00769 DRB1*11:15	26	13
HLA:HLA00697 DRB1*04:11	HLA:HLA00769 DRB1*11:15	26	14
HLA:HLA00687 DRB1*04:02	HLA:HLA01407 DRB1*12:01:02	26	16
HLA:HLA00696 DRB1*04:10	HLA:HLA01407 DRB1*12:01:02	26	18
HLA:HLA00697 DRB1*04:11	HLA:HLA01407 DRB1*12:01:02	26	19
HLA:HLA00665 DRB1*01:02:01	HLA:HLA00797 DRB1*13:01:01	26	17
HLA:HLA00685 DRB1*04:01:01	HLA:HLA00825 DRB1*13:27	26	12
HLA:HLA02172 DRB1*04:06:02	HLA:HLA00825 DRB1*13:27	26	14

Allele 1	Allele 2	*m*	K_B
HLA:HLA00693 DRB1*04:07:01	HLA:HLA00825 DRB1*13:27	26	14
HLA:HLA00694 DRB1*04:08:01	HLA:HLA00825 DRB1*13:27	26	13
HLA:HLA00696 DRB1*04:10	HLA:HLA00825 DRB1*13:27	26	14
HLA:HLA00697 DRB1*04:11	HLA:HLA00825 DRB1*13:27	26	15
HLA:HLA00688 DRB1*04:03:01	HLA:HLA00833 DRB1*14:01:01	26	9
HLA:HLA00689 DRB1*04:04:01	HLA:HLA00833 DRB1*14:01:01	26	10
HLA:HLA00692 DRB1*04:06:01	HLA:HLA00833 DRB1*14:01:01	26	9
HLA:HLA00750 DRB1*10:01:01	HLA:HLA00833 DRB1*14:01:01	26	12
HLA:HLA00688 DRB1*04:03:01	HLA:HLA00836 DRB1*14:04	26	9
HLA:HLA00689 DRB1*04:04:01	HLA:HLA00836 DRB1*14:04	26	10
HLA:HLA00692 DRB1*04:06:01	HLA:HLA00836 DRB1*14:04	26	9
HLA:HLA00750 DRB1*10:01:01	HLA:HLA00836 DRB1*14:04	26	13
HLA:HLA00688 DRB1*04:03:01	HLA:HLA00837 DRB1*14:05:01	26	7
HLA:HLA00689 DRB1*04:04:01	HLA:HLA00837 DRB1*14:05:01	26	8
HLA:HLA00692 DRB1*04:06:01	HLA:HLA00837 DRB1*14:05:01	26	7
HLA:HLA00750 DRB1*10:01:01	HLA:HLA00837 DRB1*14:05:01	26	10
HLA:HLA00688 DRB1*04:03:01	HLA:HLA00839 DRB1*14:07:01	26	10
HLA:HLA00689 DRB1*04:04:01	HLA:HLA00839 DRB1*14:07:01	26	11
HLA:HLA00690 DRB1*04:05:01	HLA:HLA00839 DRB1*14:07:01	26	11
HLA:HLA00692 DRB1*04:06:01	HLA:HLA00839 DRB1*14:07:01	26	10
HLA:HLA00695 DRB1*04:09	HLA:HLA00839 DRB1*14:07:01	26	12
HLA:HLA00665 DRB1*01:02:01	HLA:HLA02371 DRB1*14:54	26	14
HLA:HLA00685 DRB1*04:01:01	HLA:HLA02371 DRB1*14:54	26	12
HLA:HLA02172 DRB1*04:06:02	HLA:HLA02371 DRB1*14:54	26	9
HLA:HLA00693 DRB1*04:07:01	HLA:HLA02371 DRB1*14:54	26	10
HLA:HLA00694 DRB1*04:08:01	HLA:HLA02371 DRB1*14:54	26	11
HLA:HLA00696 DRB1*04:10	HLA:HLA02371 DRB1*14:54	26	11
HLA:HLA00697 DRB1*04:11	HLA:HLA02371 DRB1*14:54	26	10
HLA:HLA00839 DRB1*14:07:01	HLA:HLA00876 DRB1*16:01:01	26	14
HLA:HLA00839 DRB1*14:07:01	HLA:HLA00878 DRB1*16:02:01	26	13
HLA:HLA00687 DRB1*04:02	HLA:HLA00750 DRB1*10:01:01	27	14
HLA:HLA00687 DRB1*04:02	HLA:HLA02371 DRB1*14:54	27	15
HLA:HLA00671 DRB1*03:01:01:01	HLA:HLA00865 DRB1*15:01:01:01	27	14
HLA:HLA03483 DRB1*03:01:01:02	HLA:HLA00865 DRB1*15:01:01:01	27	14
HLA:HLA00678 DRB1*03:06	HLA:HLA00865 DRB1*15:01:01:01	27	15
HLA:HLA02257 DRB1*08:01:03	HLA:HLA00865 DRB1*15:01:01:01	27	17
HLA:HLA00727 DRB1*08:03:02	HLA:HLA00865 DRB1*15:01:01:01	27	16
HLA:HLA00735 DRB1*08:09	HLA:HLA00865 DRB1*15:01:01:01	27	16
HLA:HLA00744 DRB1*08:18	HLA:HLA00865 DRB1*15:01:01:01	27	14
HLA:HLA00749 DRB1*09:01:02	HLA:HLA00865 DRB1*15:01:01:01	27	24
HLA:HLA00751 DRB1*11:01:01	HLA:HLA00865 DRB1*15:01:01:01	27	13
HLA:HLA00769 DRB1*11:15	HLA:HLA00865 DRB1*15:01:01:01	27	15
HLA:HLA00843 DRB1*14:11	HLA:HLA00865 DRB1*15:01:01:01	27	13
HLA:HLA00671 DRB1*03:01:01:01	HLA:HLA03453 DRB1*15:01:01:02	27	14
HLA:HLA03483 DRB1*03:01:01:02	HLA:HLA03453 DRB1*15:01:01:02	27	14
HLA:HLA00678 DRB1*03:06	HLA:HLA03453 DRB1*15:01:01:02	27	15
HLA:HLA02257 DRB1*08:01:03	HLA:HLA03453 DRB1*15:01:01:02	27	17
HLA:HLA00727 DRB1*08:03:02	HLA:HLA03453 DRB1*15:01:01:02	27	16
HLA:HLA00735 DRB1*08:09	HLA:HLA03453 DRB1*15:01:01:02	27	16
HLA:HLA00744 DRB1*08:18	HLA:HLA03453 DRB1*15:01:01:02	27	14
HLA:HLA00749 DRB1*09:01:02	HLA:HLA03453 DRB1*15:01:01:02	27	24
HLA:HLA00751 DRB1*11:01:01	HLA:HLA03453 DRB1*15:01:01:02	27	13
HLA:HLA00769 DRB1*11:15	HLA:HLA03453 DRB1*15:01:01:02	27	15
HLA:HLA00843 DRB1*14:11	HLA:HLA03453 DRB1*15:01:01:02	27	13
HLA:HLA00687 DRB1*04:02	HLA:HLA00867 DRB1*15:02:01	27	13
HLA:HLA02172 DRB1*04:06:02	HLA:HLA00867 DRB1*15:02:01	27	13
HLA:HLA00724 DRB1*08:02:01	HLA:HLA00867 DRB1*15:02:01	27	14
HLA:HLA00739 DRB1*08:13	HLA:HLA00867 DRB1*15:02:01	27	14
HLA:HLA00752 DRB1*11:01:02	HLA:HLA00867 DRB1*15:02:01	27	12
HLA:HLA00755 DRB1*11:03	HLA:HLA00867 DRB1*15:02:01	27	12
HLA:HLA00756 DRB1*11:04:01	HLA:HLA00867 DRB1*15:02:01	27	13
HLA:HLA01407 DRB1*12:01:02	HLA:HLA00867 DRB1*15:02:01	27	19
HLA:HLA00825 DRB1*13:27	HLA:HLA00867 DRB1*15:02:01	27	13
HLA:HLA00833 DRB1*14:01:01	HLA:HLA00867 DRB1*15:02:01	27	17
HLA:HLA00836 DRB1*14:04	HLA:HLA00867 DRB1*15:02:01	27	16
HLA:HLA00671 DRB1*03:01:01:01	HLA:HLA00870 DRB1*15:03:01:01	27	15
HLA:HLA03483 DRB1*03:01:01:02	HLA:HLA00870 DRB1*15:03:01:01	27	15
HLA:HLA00678 DRB1*03:06	HLA:HLA00870 DRB1*15:03:01:01	27	16
HLA:HLA02257 DRB1*08:01:03	HLA:HLA00870 DRB1*15:03:01:01	27	18
HLA:HLA00727 DRB1*08:03:02	HLA:HLA00870 DRB1*15:03:01:01	27	17
HLA:HLA00735 DRB1*08:09	HLA:HLA00870 DRB1*15:03:01:01	27	17
HLA:HLA00744 DRB1*08:18	HLA:HLA00870 DRB1*15:03:01:01	27	15
HLA:HLA00749 DRB1*09:01:02	HLA:HLA00870 DRB1*15:03:01:01	27	24
HLA:HLA00751 DRB1*11:01:01	HLA:HLA00870 DRB1*15:03:01:01	27	14
HLA:HLA00769 DRB1*11:15	HLA:HLA00870 DRB1*15:03:01:01	27	16
HLA:HLA00843 DRB1*14:11	HLA:HLA00870 DRB1*15:03:01:01	27	14
HLA:HLA00671 DRB1*03:01:01:01	HLA:HLA03454 DRB1*15:03:01:02	27	15
HLA:HLA03483 DRB1*03:01:01:02	HLA:HLA03454 DRB1*15:03:01:02	27	15
HLA:HLA00678 DRB1*03:06	HLA:HLA03454 DRB1*15:03:01:02	27	16
HLA:HLA02257 DRB1*08:01:03	HLA:HLA03454 DRB1*15:03:01:02	27	18
HLA:HLA00727 DRB1*08:03:02	HLA:HLA03454 DRB1*15:03:01:02	27	17
HLA:HLA00735 DRB1*08:09	HLA:HLA03454 DRB1*15:03:01:02	27	17
HLA:HLA00744 DRB1*08:18	HLA:HLA03454 DRB1*15:03:01:02	27	15
HLA:HLA00749 DRB1*09:01:02	HLA:HLA03454 DRB1*15:03:01:02	27	24
HLA:HLA00751 DRB1*11:01:01	HLA:HLA03454 DRB1*15:03:01:02	27	14
HLA:HLA00769 DRB1*11:15	HLA:HLA03454 DRB1*15:03:01:02	27	16
HLA:HLA00843 DRB1*14:11	HLA:HLA03454 DRB1*15:03:01:02	27	14
HLA:HLA00671 DRB1*03:01:01:01	HLA:HLA00871 DRB1*15:04	27	14
HLA:HLA03483 DRB1*03:01:01:02	HLA:HLA00871 DRB1*15:04	27	14
HLA:HLA00678 DRB1*03:06	HLA:HLA00871 DRB1*15:04	27	15
HLA:HLA02257 DRB1*08:01:03	HLA:HLA00871 DRB1*15:04	27	16
HLA:HLA00727 DRB1*08:03:02	HLA:HLA00871 DRB1*15:04	27	17
HLA:HLA00735 DRB1*08:09	HLA:HLA00871 DRB1*15:04	27	15
HLA:HLA00744 DRB1*08:18	HLA:HLA00871 DRB1*15:04	27	15
HLA:HLA00749 DRB1*09:01:02	HLA:HLA00871 DRB1*15:04	27	23
HLA:HLA00751 DRB1*11:01:01	HLA:HLA00871 DRB1*15:04	27	12
HLA:HLA00769 DRB1*11:15	HLA:HLA00871 DRB1*15:04	27	14
HLA:HLA00843 DRB1*14:11	HLA:HLA00871 DRB1*15:04	27	13
HLA:HLA00688 DRB1*04:03:01	HLA:HLA00876 DRB1*16:01:01	27	13
HLA:HLA00689 DRB1*04:04:01	HLA:HLA00876 DRB1*16:01:01	27	12
HLA:HLA00692 DRB1*04:06:01	HLA:HLA00876 DRB1*16:01:01	27	12
HLA:HLA00688 DRB1*04:03:01	HLA:HLA00878 DRB1*16:02:01	27	12
HLA:HLA00689 DRB1*04:04:01	HLA:HLA00878 DRB1*16:02:01	27	11
HLA:HLA00692 DRB1*04:06:01	HLA:HLA00878 DRB1*16:02:01	27	11
HLA:HLA00671 DRB1*03:01:01:01	HLA:HLA00685 DRB1*04:01:01	27	10
HLA:HLA03483 DRB1*03:01:01:02	HLA:HLA00685 DRB1*04:01:01	27	10
HLA:HLA00678 DRB1*03:06	HLA:HLA00685 DRB1*04:01:01	27	9
HLA:HLA00671 DRB1*03:01:01:01	HLA:HLA00687 DRB1*04:02	27	13
HLA:HLA03483 DRB1*03:01:01:02	HLA:HLA00687 DRB1*04:02	27	13
HLA:HLA00678 DRB1*03:06	HLA:HLA00687 DRB1*04:02	27	12
HLA:HLA00672 DRB1*03:01:02	HLA:HLA00688 DRB1*04:03:01	27	10
HLA:HLA00672 DRB1*03:01:02	HLA:HLA00689 DRB1*04:04:01	27	10
HLA:HLA00672 DRB1*03:01:02	HLA:HLA00692 DRB1*04:06:01	27	11
HLA:HLA00671 DRB1*03:01:01:01	HLA:HLA02172 DRB1*04:06:02	27	11
HLA:HLA03483 DRB1*03:01:01:02	HLA:HLA02172 DRB1*04:06:02	27	11
HLA:HLA00678 DRB1*03:06	HLA:HLA02172 DRB1*04:06:02	27	10
HLA:HLA00671 DRB1*03:01:01:01	HLA:HLA00693 DRB1*04:07:01	27	11
HLA:HLA03483 DRB1*03:01:01:02	HLA:HLA00693 DRB1*04:07:01	27	11
HLA:HLA00678 DRB1*03:06	HLA:HLA00693 DRB1*04:07:01	27	10
HLA:HLA00671 DRB1*03:01:01:01	HLA:HLA00694 DRB1*04:08:01	27	11
HLA:HLA03483 DRB1*03:01:01:02	HLA:HLA00694 DRB1*04:08:01	27	11
HLA:HLA00678 DRB1*03:06	HLA:HLA00694 DRB1*04:08:01	27	10
HLA:HLA00671 DRB1*03:01:01:01	HLA:HLA00696 DRB1*04:10	27	12
HLA:HLA03483 DRB1*03:01:01:02	HLA:HLA00696 DRB1*04:10	27	12
HLA:HLA00678 DRB1*03:06	HLA:HLA00696 DRB1*04:10	27	11
HLA:HLA00671 DRB1*03:01:01:01	HLA:HLA00697 DRB1*04:11	27	12
HLA:HLA03483 DRB1*03:01:01:02	HLA:HLA00697 DRB1*04:11	27	12
HLA:HLA00678 DRB1*03:06	HLA:HLA00697 DRB1*04:11	27	11
HLA:HLA02172 DRB1*04:06:02	HLA:HLA02257 DRB1*08:01:03	27	13
HLA:HLA02172 DRB1*04:06:02	HLA:HLA00727 DRB1*08:03:02	27	13
HLA:HLA02172 DRB1*04:06:02	HLA:HLA00735 DRB1*08:09	27	12
HLA:HLA00696 DRB1*04:10	HLA:HLA00735 DRB1*08:09	27	14
HLA:HLA00697 DRB1*04:11	HLA:HLA00735 DRB1*08:09	27	14
HLA:HLA02172 DRB1*04:06:02	HLA:HLA00744 DRB1*08:18	27	12
HLA:HLA00672 DRB1*03:01:02	HLA:HLA00750 DRB1*10:01:01	27	14
HLA:HLA02172 DRB1*04:06:02	HLA:HLA00752 DRB1*11:01:02	27	11
HLA:HLA00696 DRB1*04:10	HLA:HLA00752 DRB1*11:01:02	27	11
HLA:HLA00697 DRB1*04:11	HLA:HLA00752 DRB1*11:01:02	27	12
HLA:HLA00665 DRB1*01:02:01	HLA:HLA00755 DRB1*11:03	27	15
HLA:HLA00665 DRB1*01:02:01	HLA:HLA00756 DRB1*11:04:01	27	13
HLA:HLA00665 DRB1*01:02:01	HLA:HLA02157 DRB1*11:11:02	27	16
HLA:HLA00688 DRB1*04:03:01	HLA:HLA01407 DRB1*12:01:02	27	18
HLA:HLA00689 DRB1*04:04:01	HLA:HLA01407 DRB1*12:01:02	27	17
HLA:HLA00690 DRB1*04:05:01	HLA:HLA01407 DRB1*12:01:02	27	19
HLA:HLA00692 DRB1*04:06:01	HLA:HLA01407 DRB1*12:01:02	27	18
HLA:HLA00695 DRB1*04:09	HLA:HLA01407 DRB1*12:01:02	27	20
HLA:HLA00665 DRB1*01:02:01	HLA:HLA00798 DRB1*13:02:01	27	18
HLA:HLA00665 DRB1*01:02:01	HLA:HLA00802 DRB1*13:05:01	27	16
HLA:HLA00665 DRB1*01:02:01	HLA:HLA00810 DRB1*13:12:01	27	15
HLA:HLA00749 DRB1*09:01:02	HLA:HLA00810 DRB1*13:12:01	27	18
HLA:HLA00690 DRB1*04:05:01	HLA:HLA00825 DRB1*13:27	27	15
HLA:HLA00695 DRB1*04:09	HLA:HLA00825 DRB1*13:27	27	14
HLA:HLA00665 DRB1*01:02:01	HLA:HLA01724 DRB1*13:56	27	15
HLA:HLA00665 DRB1*01:02:01	HLA:HLA00833 DRB1*14:01:01	27	14
HLA:HLA00685 DRB1*04:01:01	HLA:HLA00833 DRB1*14:01:01	27	12
HLA:HLA02172 DRB1*04:06:02	HLA:HLA00833 DRB1*14:01:01	27	9
HLA:HLA00693 DRB1*04:07:01	HLA:HLA00833 DRB1*14:01:01	27	10
HLA:HLA00694 DRB1*04:08:01	HLA:HLA00833 DRB1*14:01:01	27	11
HLA:HLA00696 DRB1*04:10	HLA:HLA00833 DRB1*14:01:01	27	11
HLA:HLA00697 DRB1*04:11	HLA:HLA00833 DRB1*14:01:01	27	10
HLA:HLA00665 DRB1*01:02:01	HLA:HLA00836 DRB1*14:04	27	15
HLA:HLA00685 DRB1*04:01:01	HLA:HLA00836 DRB1*14:04	27	12
HLA:HLA02172 DRB1*04:06:02	HLA:HLA00836 DRB1*14:04	27	9
HLA:HLA00693 DRB1*04:07:01	HLA:HLA00836 DRB1*14:04	27	10
HLA:HLA00694 DRB1*04:08:01	HLA:HLA00836 DRB1*14:04	27	11
HLA:HLA00696 DRB1*04:10	HLA:HLA00836 DRB1*14:04	27	11
HLA:HLA00697 DRB1*04:11	HLA:HLA00836 DRB1*14:04	27	10
HLA:HLA00665 DRB1*01:02:01	HLA:HLA00837 DRB1*14:05:01	27	12
HLA:HLA00685 DRB1*04:01:01	HLA:HLA00837 DRB1*14:05:01	27	10
HLA:HLA02172 DRB1*04:06:02	HLA:HLA00837 DRB1*14:05:01	27	7
HLA:HLA00693 DRB1*04:07:01	HLA:HLA00837 DRB1*14:05:01	27	8
HLA:HLA00694 DRB1*04:08:01	HLA:HLA00837 DRB1*14:05:01	27	9
HLA:HLA00696 DRB1*04:10	HLA:HLA00837 DRB1*14:05:01	27	10
HLA:HLA00697 DRB1*04:11	HLA:HLA00837 DRB1*14:05:01	27	9
HLA:HLA00665 DRB1*01:02:01	HLA:HLA00839 DRB1*14:07:01	27	15
HLA:HLA02172 DRB1*04:06:02	HLA:HLA00839 DRB1*14:07:01	27	10
HLA:HLA00696 DRB1*04:10	HLA:HLA00839 DRB1*14:07:01	27	12
HLA:HLA00697 DRB1*04:11	HLA:HLA00839 DRB1*14:07:01	27	11
HLA:HLA00688 DRB1*04:03:01	HLA:HLA00843 DRB1*14:11	27	7
HLA:HLA00689 DRB1*04:04:01	HLA:HLA00843 DRB1*14:11	27	8
HLA:HLA00692 DRB1*04:06:01	HLA:HLA00843 DRB1*14:11	27	7
HLA:HLA00750 DRB1*10:01:01	HLA:HLA00843 DRB1*14:11	27	11
HLA:HLA00690 DRB1*04:05:01	HLA:HLA02371 DRB1*14:54	27	12
HLA:HLA00695 DRB1*04:09	HLA:HLA02371 DRB1*14:54	27	13
HLA:HLA00749 DRB1*09:01:02	HLA:HLA00876 DRB1*16:01:01	27	20
HLA:HLA00837 DRB1*14:05:01	HLA:HLA00876 DRB1*16:01:01	27	13
HLA:HLA02371 DRB1*14:54	HLA:HLA00876 DRB1*16:01:01	27	15
HLA:HLA00749 DRB1*09:01:02	HLA:HLA00878 DRB1*16:02:01	27	21
HLA:HLA00837 DRB1*14:05:01	HLA:HLA00878 DRB1*16:02:01	27	12
HLA:HLA02371 DRB1*14:54	HLA:HLA00878 DRB1*16:02:01	27	14
HLA:HLA00665 DRB1*01:02:01	HLA:HLA00719 DRB1*07:01:01:01	28	18
HLA:HLA00665 DRB1*01:02:01	HLA:HLA03486 DRB1*07:01:01:02	28	18
HLA:HLA00665 DRB1*01:02:01	HLA:HLA02017 DRB1*07:09	28	17
HLA:HLA00687 DRB1*04:02	HLA:HLA00833 DRB1*14:01:01	28	15
HLA:HLA00687 DRB1*04:02	HLA:HLA00836 DRB1*14:04	28	15
HLA:HLA00687 DRB1*04:02	HLA:HLA00837 DRB1*14:05:01	28	13

Allele 1	Allele 2	m	K_B
HLA:HLA00687 DRB1*04:02	HLA:HLA00839 DRB1*14:07:01	28	16
HLA:HLA00719 DRB1*07:01:01:01	HLA:HLA01693 DRB1*14:46	28	19
HLA:HLA03486 DRB1*07:01:01:02	HLA:HLA01693 DRB1*14:46	28	19
HLA:HLA02017 DRB1*07:09	HLA:HLA01693 DRB1*14:46	28	18
HLA:HLA00672 DRB1*03:01:02	HLA:HLA00865 DRB1*15:01:01:01	28	14
HLA:HLA00724 DRB1*08:02:01	HLA:HLA00865 DRB1*15:01:01:01	28	15
HLA:HLA00739 DRB1*08:13	HLA:HLA00865 DRB1*15:01:01:01	28	15
HLA:HLA00752 DRB1*11:01:02	HLA:HLA00865 DRB1*15:01:01:01	28	13
HLA:HLA00672 DRB1*03:01:02	HLA:HLA03453 DRB1*15:01:01:02	28	14
HLA:HLA00724 DRB1*08:02:01	HLA:HLA03453 DRB1*15:01:01:02	28	15
HLA:HLA00739 DRB1*08:13	HLA:HLA03453 DRB1*15:01:01:02	28	15
HLA:HLA00752 DRB1*11:01:02	HLA:HLA03453 DRB1*15:01:01:02	28	13
HLA:HLA00671 DRB1*03:01:01:01	HLA:HLA00867 DRB1*15:02:01	28	15
HLA:HLA03483 DRB1*03:01:01:02	HLA:HLA00867 DRB1*15:02:01	28	15
HLA:HLA00678 DRB1*03:06	HLA:HLA00867 DRB1*15:02:01	28	16
HLA:HLA00843 DRB1*14:11	HLA:HLA00867 DRB1*15:02:01	28	14
HLA:HLA00672 DRB1*03:01:02	HLA:HLA00870 DRB1*15:03:01:01	28	15
HLA:HLA00724 DRB1*08:02:01	HLA:HLA00870 DRB1*15:03:01:01	28	16
HLA:HLA00739 DRB1*08:13	HLA:HLA00870 DRB1*15:03:01:01	28	16
HLA:HLA00752 DRB1*11:01:02	HLA:HLA00870 DRB1*15:03:01:01	28	14
HLA:HLA00672 DRB1*03:01:02	HLA:HLA03454 DRB1*15:03:01:02	28	15
HLA:HLA00724 DRB1*08:02:01	HLA:HLA03454 DRB1*15:03:01:02	28	16
HLA:HLA00739 DRB1*08:13	HLA:HLA03454 DRB1*15:03:01:02	28	16
HLA:HLA00752 DRB1*11:01:02	HLA:HLA03454 DRB1*15:03:01:02	28	14
HLA:HLA00672 DRB1*03:01:02	HLA:HLA00871 DRB1*15:04	28	14
HLA:HLA00724 DRB1*08:02:01	HLA:HLA00871 DRB1*15:04	28	14
HLA:HLA00739 DRB1*08:13	HLA:HLA00871 DRB1*15:04	28	15
HLA:HLA00752 DRB1*11:01:02	HLA:HLA00871 DRB1*15:04	28	12
HLA:HLA02172 DRB1*04:06:02	HLA:HLA00876 DRB1*16:01:01	28	12
HLA:HLA02172 DRB1*04:06:02	HLA:HLA00878 DRB1*16:02:01	28	11
HLA:HLA00672 DRB1*03:01:02	HLA:HLA00685 DRB1*04:01:01	28	10
HLA:HLA00672 DRB1*03:01:02	HLA:HLA00687 DRB1*04:02	28	13
HLA:HLA00671 DRB1*03:01:01:01	HLA:HLA00690 DRB1*04:05:01	28	13
HLA:HLA03483 DRB1*03:01:01:02	HLA:HLA00690 DRB1*04:05:01	28	13
HLA:HLA00678 DRB1*03:06	HLA:HLA00690 DRB1*04:05:01	28	12
HLA:HLA00672 DRB1*03:01:02	HLA:HLA02172 DRB1*04:06:02	28	11
HLA:HLA00672 DRB1*03:01:02	HLA:HLA00693 DRB1*04:07:01	28	11
HLA:HLA00672 DRB1*03:01:02	HLA:HLA00694 DRB1*04:08:01	28	11
HLA:HLA00671 DRB1*03:01:01:01	HLA:HLA00695 DRB1*04:09	28	12
HLA:HLA03483 DRB1*03:01:01:02	HLA:HLA00695 DRB1*04:09	28	12
HLA:HLA00678 DRB1*03:06	HLA:HLA00695 DRB1*04:09	28	11
HLA:HLA00672 DRB1*03:01:02	HLA:HLA00696 DRB1*04:10	28	12
HLA:HLA00672 DRB1*03:01:02	HLA:HLA00697 DRB1*04:11	28	12
HLA:HLA00665 DRB1*01:02:01	HLA:HLA00724 DRB1*08:02:01	28	16
HLA:HLA00665 DRB1*01:02:01	HLA:HLA00739 DRB1*08:13	28	15
HLA:HLA00665 DRB1*01:02:01	HLA:HLA00751 DRB1*11:01:01	28	14
HLA:HLA00665 DRB1*01:02:01	HLA:HLA00769 DRB1*11:15	28	16
HLA:HLA00685 DRB1*04:01:01	HLA:HLA01407 DRB1*12:01:02	28	19
HLA:HLA02172 DRB1*04:06:02	HLA:HLA01407 DRB1*12:01:02	28	18
HLA:HLA00693 DRB1*04:07:01	HLA:HLA01407 DRB1*12:01:02	28	19
HLA:HLA00694 DRB1*04:08:01	HLA:HLA01407 DRB1*12:01:02	28	18
HLA:HLA00665 DRB1*01:02:01	HLA:HLA00825 DRB1*13:27	28	17
HLA:HLA00690 DRB1*04:05:01	HLA:HLA00833 DRB1*14:01:01	28	12
HLA:HLA00695 DRB1*04:09	HLA:HLA00833 DRB1*14:01:01	28	13
HLA:HLA00690 DRB1*04:05:01	HLA:HLA00836 DRB1*14:04	28	12
HLA:HLA00695 DRB1*04:09	HLA:HLA00836 DRB1*14:04	28	13
HLA:HLA00690 DRB1*04:05:01	HLA:HLA00837 DRB1*14:05:01	28	11
HLA:HLA00695 DRB1*04:09	HLA:HLA00837 DRB1*14:05:01	28	12
HLA:HLA00665 DRB1*01:02:01	HLA:HLA00843 DRB1*14:11	28	13
HLA:HLA00685 DRB1*04:01:01	HLA:HLA00843 DRB1*14:11	28	10
HLA:HLA02172 DRB1*04:06:02	HLA:HLA00843 DRB1*14:11	28	7
HLA:HLA00693 DRB1*04:07:01	HLA:HLA00843 DRB1*14:11	28	8
HLA:HLA00694 DRB1*04:08:01	HLA:HLA00843 DRB1*14:11	28	9
HLA:HLA00696 DRB1*04:10	HLA:HLA00843 DRB1*14:11	28	10
HLA:HLA00697 DRB1*04:11	HLA:HLA00843 DRB1*14:11	28	9
HLA:HLA00833 DRB1*14:01:01	HLA:HLA00876 DRB1*16:01:01	28	15
HLA:HLA00836 DRB1*14:04	HLA:HLA00876 DRB1*16:01:01	28	14
HLA:HLA00833 DRB1*14:01:01	HLA:HLA00878 DRB1*16:02:01	28	14
HLA:HLA00836 DRB1*14:04	HLA:HLA00878 DRB1*16:02:01	28	13
