# Supplementary material for: Nonsynonymous Substitution Rate Heterogeneity in the Peptide-Binding Region Among Different HLA-DRB1 Lineages in Humans
Source: G3 (Bethesda). 2014 May 2;4(7):1217–26. doi: 10.1534/g3.114.011726 (PMC4455771; doi:10.1534/g3.114.011726)
Supplement: Supporting Information [file supp_g3.114.011726_TableS2.pdf]

Table S2 Parallel substitution sites in the PBR among allele pairs over all phases

			24 sites in the PBR (Brown *et al*. 1993) and 3 sites in peptide-binding pockets (Stern *et al*. 1994)																										
	m	No. of allele pairs	9	11	13	28	30	32	37	38	47	56	57	60	61	65	67	68	70	71	74	78	81	82	85	86	88	89	90
Phase I	0	32																											
	1	58																											
	2	40																											
	3	32																											
	4	39																											
	5	30																											
	6	45																											
	7	58																											
	8	64																											
	9	50																											
	10	30																											
	11	24																											
	12	24																											
	13	9																											
	14																												
	15																												
	16																												
	17																												
	18																												
	19	2																											
Phase II	20	16																											
	21	18																											
	22	27																											
	23	54																											
	24	102																											
	25	185																											
	26	214																											
	27	160																											
	28	75																											
Phase III	29	29																											
	30	24																											
Phase IV	31	25																											
	32	31																											
	33	28																											
	34	15																											

Red rectangle represents the parallel substitution site of the PBR in allele pairs which have same *m* value. Yellow rectangle represents the segregating site of the PBR in allele pairs which have same *m* value.
