# Supplementary material for: Nonsynonymous Substitution Rate Heterogeneity in the Peptide-Binding Region Among Different HLA-DRB1 Lineages in Humans
Source: G3 (Bethesda). 2014 May 2;4(7):1217–26. doi: 10.1534/g3.114.011726 (PMC4455771; doi:10.1534/g3.114.011726)
Supplement: Supporting Information [file supp_g3.114.011726_TableS3.pdf]

Table S3 The specific pathogens bound by HLA-DRB1 allelic lineages only with fast or slow PBR substitution rate

<table>
<tr><th>HLA-DRB1 molecule</th><th>Source Organism ID</th><th>Source Organism Name</th><th>PBR substitution rate</th></tr>
<tr><td>HLA-DRB1*03</td><td>10580</td><td>Human papillomavirus type 11</td><td>Fast</td></tr>
<tr><td rowspan="9">HLA-DRB1*04</td><td>236</td><td>Brucella ovis</td><td rowspan="12">Slow</td></tr>
<tr><td>1358</td><td>Lactococcus lactis</td></tr>
<tr><td>10254</td><td>Vaccinia virus WR</td></tr>
<tr><td>11309</td><td>Herpes simplex virus (type 1 / strain SC16)</td></tr>
<tr><td>211044</td><td>Influenza A virus (A/Puerto Rico/8/1934(H1N1))</td></tr>
<tr><td>243160</td><td>Burkholderia mallei ATCC 23344</td></tr>
<tr><td>381512</td><td>Influenza A virus (A/New Caledonia/20/1999(H1N1))</td></tr>
<tr><td>381513</td><td>Influenza A virus (A/Panama/2007/1999(H3N2))</td></tr>
<tr><td>641501</td><td>Influenza A virus (A/California/04/2009(H1N1))</td></tr>
<tr><td rowspan="3">HLA-DRB1*15</td><td>1313</td><td>Streptococcus pneumoniae</td></tr>
<tr><td>1423</td><td>Bacillus subtilis</td></tr>
<tr><td>1764</td><td>Mycobacterium avium</td></tr>
</table>

The dataset of source organism bound to HLA-DRB1 molecules was acquired from the MHC binding assay in the IEDB database (*http://www.iedb.org/*).
